# EcoTILLING-Based Association Mapping Efficiently Delineates Functionally Relevant Natural Allelic Variants of Candidate Genes Governing Agronomic Traits in Chickpea

**DOI:** 10.3389/fpls.2016.00450

**Published:** 2016-04-19

**Authors:** Deepak Bajaj, Rishi Srivastava, Manoj Nath, Shailesh Tripathi, Chellapilla Bharadwaj, Hari D. Upadhyaya, Akhilesh K. Tyagi, Swarup K. Parida

**Affiliations:** ^1^Govt. of India, Plant Genomics and Molecular Breeding Lab, Department of Biotechnology, National Institute of Plant Genome ResearchNew Delhi, India; ^2^National Research Centre on Plant BiotechnologyNew Delhi, India; ^3^Division of Genetics, Indian Agricultural Research InstituteNew Delhi, India; ^4^International Crops Research Institute for the Semi-Arid TropicsPatancheru, India

**Keywords:** allele, association mapping, chickpea, EcoTILLING, seed weight, SNP, transcription factor

## Abstract

The large-scale mining and high-throughput genotyping of novel gene-based allelic variants in natural mapping population are essential for association mapping to identify functionally relevant molecular tags governing useful agronomic traits in chickpea. The present study employs an alternative time-saving, non-laborious and economical pool-based EcoTILLING approach coupled with agarose gel detection assay to discover 1133 novel SNP allelic variants from diverse coding and regulatory sequence components of 1133 transcription factor (TF) genes by genotyping in 192 diverse *desi* and *kabuli* chickpea accessions constituting a seed weight association panel. Integrating these SNP genotyping data with seed weight field phenotypic information of 192 structured association panel identified eight SNP alleles in the eight TF genes regulating seed weight of chickpea. The associated individual and combination of all SNPs explained 10–15 and 31% phenotypic variation for seed weight, respectively. The EcoTILLING-based large-scale allele mining and genotyping strategy implemented for association mapping is found much effective for a diploid genome crop species like chickpea with narrow genetic base and low genetic polymorphism. This optimized approach thus can be deployed for various genomics-assisted breeding applications with optimal expense of resources in domesticated chickpea. The seed weight-associated natural allelic variants and candidate TF genes delineated have potential to accelerate marker-assisted genetic improvement of chickpea.

## Introduction

Allele mining is an efficient strategy to unlock a wealth of largely untapped natural and functional allelic variation/diversity existing within wild and cultivated genetic resources for crop genetic enhancement, thereby improving the productivity and sustainability of global agriculture. The vast available germplasm (core and mini-core) repositories and different recently developed high-throughput array-based next-generation sequencing (NGS) and sequence-based marker genotyping strategies are found expedient in large-scale mining and genotyping of genome/gene-based SNP (single nucleotide polymorphism) alleles among these germplasm accessions for driving genomics-assisted crop improvement through genetic and association mapping. The allele mining strategies commonly adopted in laboratories equipped with advanced infrastructural facilities (like high-throughput genotyping platforms and modern computational genomics tools), require prior information of SNP alleles (nature/types and flanking sequences) for their discovery, validation and genotyping in the targeted gene/genomic regions of multiple crop accessions. Conversely, EcoTILLING (Ecotype Targeting Induced Local Lesions IN Genomes), a rapid, inexpensive and well-established allele mining approach is found much proficient in large-scale mining and high-throughput genotyping of novel natural and functional allelic variants (without prior knowledge of SNP alleles) of known and candidate genes related to useful agronomic traits in diverse crop germplasm accessions (McCallum et al., 2000; Comai et al., 2004; Till et al., 2006, 2007, 2010; Raghavan et al., 2007; Wang et al., 2010; Xia et al., 2012). The implication of EcoTILLING to identify potential novel functional alleles in the known and candidate genes/transcription factors (TFs) regulating qualitative and quantitative agronomic traits by association/genetic mapping is well-documented for expediting the genetic enhancement of crop plants (Mejlhede et al., 2006; Barkley and Wang, 2008; Ibiza et al., 2010; Negrao et al., 2011; Yu et al., 2012; Frerichmann et al., 2013).

EcoTILLING usually employs a mismatch-specific CEL-I nuclease to cleave the PCR amplified fragments at the site of heteroduplex formation involving nucleotide (SNP-allelic) polymorphism. Most of the EcoTILLING studies utilize the advanced genotyping platforms (LICOR NEN Model 4300 DNA Analyzer, Transgenomic WAVE-HS denaturing high performance liquid chromatography, ABI 377 sequencer and eGene capillary electrophoresis systems) for efficient resolution of fluorescent dye (IRDye 700/800 and SYBR green)-labeled CEL-I cleaved heteroduplex PCR amplified fragments. Consequently, these efforts led to the discovery and genotyping of novel potential alleles specifically derived from the trait-associated known and candidate genes in natural population of diverse crop plants (Perry et al., 2003; Caldwell et al., 2004; Comai et al., 2004; Henikoff et al., 2004; Yang et al., 2004; Suzuki et al., 2005). The added-advantage of agarose gel-based EcoTILLING vis-à-vis the commonly utilized LICOR genotyper for large-scale mining and genotyping of allelic variants in accessions exhibiting low level polymorphism, is well-demonstrated in many crop plants (Raghavan et al., 2007; Negrao et al., 2011; Yu et al., 2012). This is merely because efficacy of an agarose gel-based EcoTILLING approach in precise resolution of unlabeled CEL I-cleaved heteroduplex PCR amplified fragments by a simpler, economical and time-saving agarose gel-based detection assay as compared to a standard EcoTILLING method that requires labeled CEL I-cleaved heteroduplex PCR amplicons for resolution in a LICOR genotyper. The broader utility and deployment of this agarose gel-based EcoTILLING approach in manifold large-scale genotyping applications is well-documented by the research laboratories with minimal resources (Raghavan et al., 2007; Negrao et al., 2011; Yu et al., 2012). This includes understanding the natural allelic diversity, population genetic structure and domestication pattern among accessions, molecular mapping and genetic association analysis for identification of potential molecular tags like alleles and genes/QTLs (quantitative trait loci) governing vital agronomic traits and marker-assisted breeding for selecting desirable accessions for crop genetic improvement.

Chickpea, a member of genus *Cicer*, is rich in cultivated and wild germplasm resource (core/mini-core collections) with a wealth of trait diversity (Upadhyaya and Ortiz, 2001; Upadhyaya et al., 2001, 2002). More in-depth characterization of these core/mini-core germplasm resources at both genotypic and phenotypic level for diverse important abiotic/biotic stress tolerance and yield/quality component traits is essential to discover and deploy valuable alleles and allelic combinations scanned from these germplasm accessions, more effectively for genetic improvement of chickpea (Upadhyaya et al., 2008, 2011; Varshney et al., 2013a; Saxena et al., 2014a,b; Bajaj et al., 2015). The existing diverse germplasm collections are thus “gold mines” for analysis of functional as well as natural allelic variation/diversity in the known and candidate genes controlling important agronomic traits of chickpea. Considering the importance of allele mining in crop genetic enhancement, EcoTILLING can be employed in multiple cultivated (*desi* and *kabuli*) and wild chickpea accessions for identifying novel functional/natural allelic variants in the candidate and known genes associated with multiple traits of agricultural importance in chickpea.

The agarose gel-based EcoTILLING strategy mostly utilizes pooling of genomic DNA isolated from two diverse accessions rather than multiple accessions for robust mining and genotyping of alleles in the view of anticipating more allelic variations between distant accessions of crop plants (Raghavan et al., 2007). However, the level of allelic variation and diversity captured specifically from different sequence components of genes/genomes among germplasm accessions of chickpea is known to be very low due to its narrow genetic base and extensive domestication bottlenecks as compared to other crop plants (Abbo et al., 2003, 2005; Berger et al., 2003, 2005; Singh et al., 2008; Toker, 2009; Jain et al., 2013; Varshney et al., 2013b; Saxena et al., 2014a). Therefore, in a diploid self-pollinated crop species like chickpea with a lower occurrence of SNP-allelic variations, the agarose gel-based EcoTILLING approach can easily be expanded to multiple accessions regardless of selecting only two accessions for DNA pooling in allele mining. Consequently, the efficient resolution and estimation of allelic variants scanned from the pooled DNA of multiple chickpea accessions will be relatively convenient, even in a low-resolution agarose gel than that of other crop species exhibiting higher allelic polymorphism. Such a strategy of multiple accessions pooling-based EcoTILLING coupled with agarose gel detection approach has been found beneficial for various high-throughput allele mining and large-scale genotyping applications, including genetic and association mapping of alleles/genes (TFs) regulating drought and salinity stress tolerance traits in rice (Negrao et al., 2011; Yu et al., 2012). Henceforth, the utilization of this multiple accessions-pooling agarose gel-based EcoTILLING approach can certainly accelerate the process of rapid selection of informative SNP alleles/markers as well as identification of accessions exhibiting higher allelic variations for their robust genotyping at a genome-wide scale. This strategy will be thus useful for various high-throughput genetic analysis in chickpea with sub-optimal use of resources. A large-scale novel as well as functional allelic genotyping information cataloged from diverse germplasm (core/mini-core) accessions and bi-parental mapping populations by use of agarose gel-based EcoTILLING assay can serve as a vital resource for trait association and genetic mapping. This will be helpful to identify favorable natural allelic variants undergoing selection during the course of domestication in *desi, kabuli* and wild accessions that are adapted to diverse agro-climatic conditions for genomics-assisted crop improvement of chickpea.

**Figure 1:**
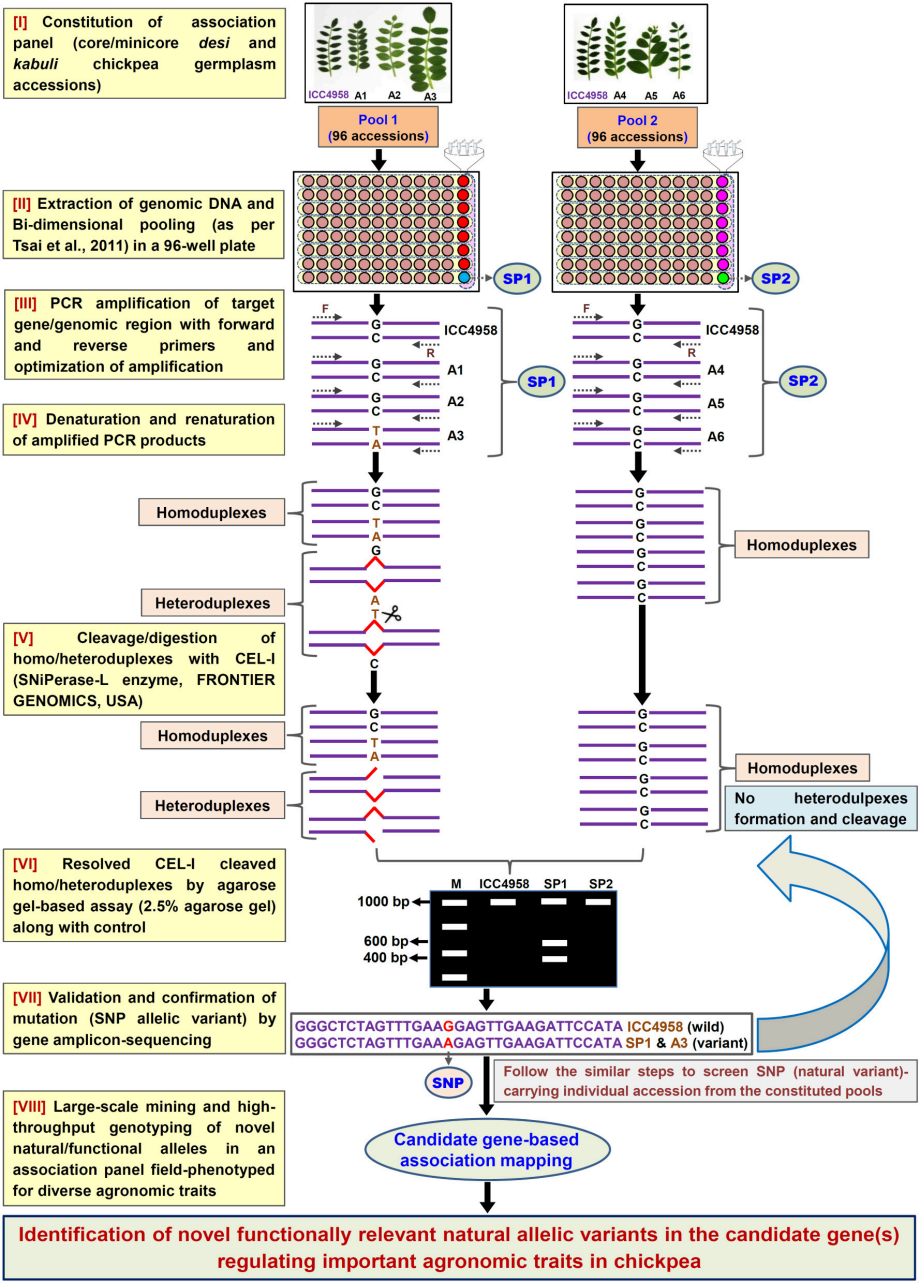
**Schematic depicting the major steps followed in an agarose gel-based EcoTILLING assay for efficient identification of functionally relevant molecular tags governing useful agronomic traits in chickpea**. This strategy is optimized for successful large-scale mining of novel SNP allelic variants from the target genomic regions (genes) by genotyping in a constituted field-phenotyped association panel (*desi* and *kabuli* core/mini-core germplasm lines). A, Accessions; SP, Superpool; F, (Forward); and R, (Reverse) primers.

In light of the above, the present study employed a simpler non-laborious and rapid yet cost-effective agarose gel-based EcoTILLING assay (Figure 1) for high-throughput mining of natural allelic variants derived from diverse coding and non-coding regulatory sequence components of 1133 TF genes by genotyping in 192 core/mini-core germplasm accessions constituting a seed weight association panel. As a proof of concept, the high-throughput genotyping data of 1133 TF gene-derived SNPs was correlated with seed weight field phenotypic information of the 192 accessions to delineate functionally relevant natural allelic variants in the candidate TF genes regulating 100-seed weight in chickpea.

## Agarose gel-based EcoTILLING aids in mining of novel natural and functional allelic variants in chickpea

**Figure 2:**
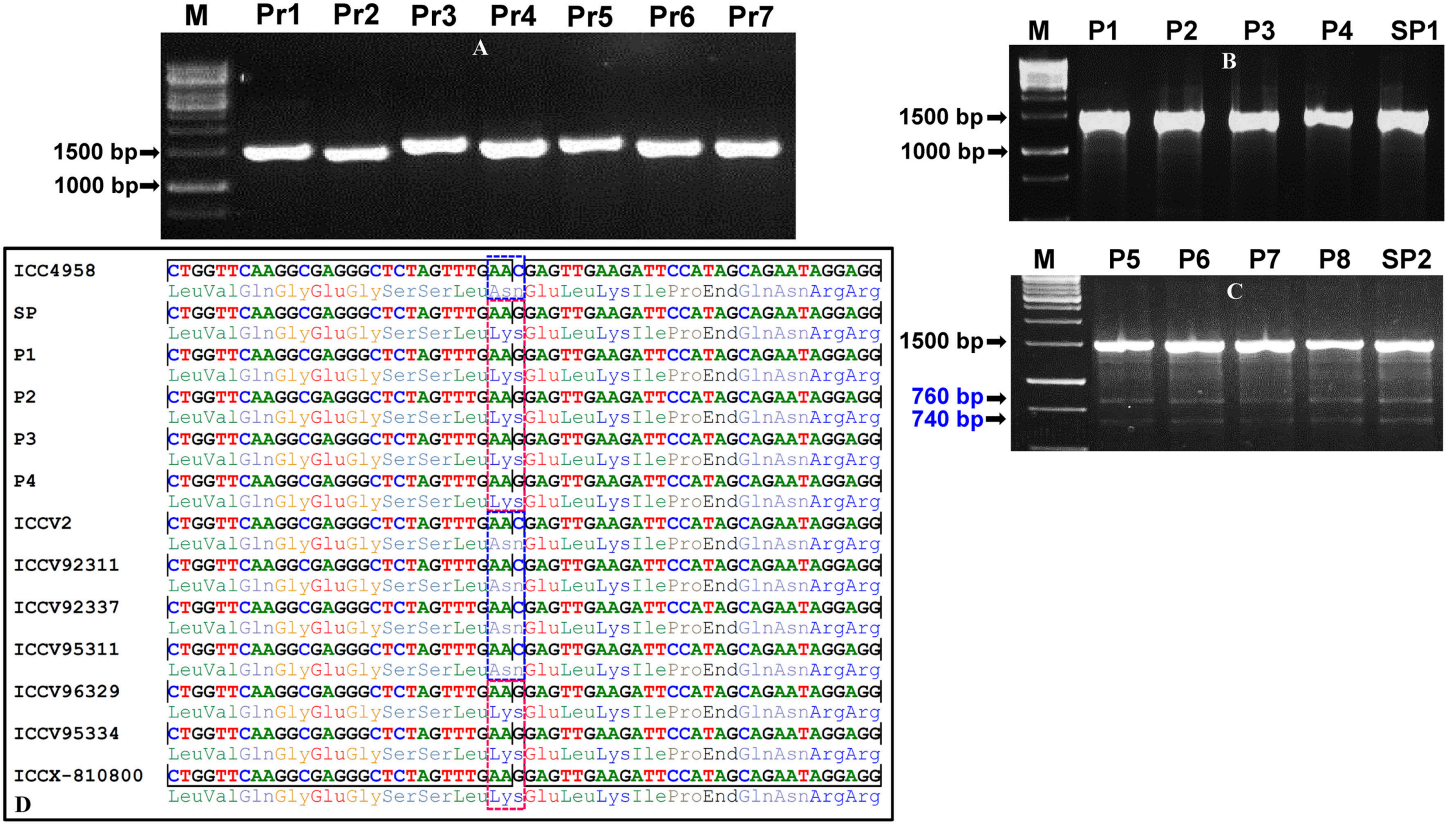
**Optimization and validation of pool-based EcoTILLING approach coupled with agarose gel detection assay for large-scale mining of novel allelic variants from diverse coding and regulatory sequence components of TF genes by genotyping in a 192 ***desi*** and ***kabuli*** germplasm lines belonging to a seed weight association panel. (A)** A representative gel illustrating the optimization followed by PCR amplification of seven primer-pairs (Pr1-Pr7) designed targeting various coding and regulatory regions of seven TF genes in the genomic DNA of a *desi* chickpea accession (ICC 4958) to produce single reproducible amplicons of each primer for EcoTILLING analysis. **(B,C)** The representative gels depicting the screening of allelic variants from the eight representative micropools (P1–P8) and two superpools (SP1 and SP2) made from the genomic DNA of 192 *desi* and *kabuli* germplasm lines (including ICC 4958 as control) employing an agarose gel-based EcoTILLING assay as defined in the Figure 1. The absence **(B)** and presence **(C)** of one non-synonymous SNP allelic variant in the pools and superpools based on cleavage/digestion patterns of 1500 bp fragments amplified from the target CDS region of a mTERF TF gene was apparent in 2.5% agarose gel. The occurrence of 1500 bp homoduplex uncut PCR amplicons as well as mismatch-specific CEL I cleavage of 1500 bp heteroduplex PCR amplified fragments into two varied amplicons of 760 and 740 bp fragment sizes due to the effect of single nucleotide polymorphism (SNP-allele) was observed in the four pools (P5–P8) and one superpool (SP2). M: 1 kb DNA ladder size standard. **(D)** The sequencing of 1500 bp amplified PCR product of an mTERF TF gene followed by multiple alignment of their high-quality sequences ascertained the presence of one coding SNP (C to G) exhibiting missense non-synonymous amino acid substitution [aspargine (AAC) to lysine (AAG)] in the four pools (P5–P8), one superpool (SP2) and seven individual *desi* and *kabuli* accessions as per expectation based on agarose gel-based EcoTILLING assay. The sequenced region carrying the non-synonymous SNP is indicated with a dotted box. The detail information of genes used for validation is mentioned in the Table S2.

For large-scale mining and genotyping of gene-based SNP alleles by EcoTILLING in chickpea, a set of 1248 TF genes annotated from *desi* and *kabuli* genomes were acquired. The selected TFs include 819 *desi* and *kabuli* TF genes and 429 TF-encoding transcripts of *desi* accession (ICC 4958), which were specifically selected from the previous studies of Jhanwar et al. (2012) and Kujur et al. (2015), respectively based on the presence of at least one SNP in the CDS (coding sequence) and regulatory sequences of these genes. The multiple forward and reverse primer combination (at least two primer-pairs per TF gene) with expected amplification product size of 1000–1500 bp (per primer) targeting the diverse CDS and 2000-bp upstream/downstream regulatory regions (URRs/DRRs) of 1248 TF genes were designed. The amplification of each target gene regions was optimized (specifically the annealing temperature) with different combination of primer-pairs using the genomic DNA of one *desi* chickpea accession (ICC 4958) as per the detail PCR protocol described by Jhanwar et al. (2012) and Kujur et al. (2013). Based on these analyses, 1890 (75.7% of 2496 primer-pairs designed in total) primers designed from the 1133 TF genes exhibited reproducible single amplicons (by eliminating the non-specific amplified fragments and duplicate loci) in ICC 4958 using 2.5% agarose gel (Figure 2A). The fragments amplified specific to diverse coding (CDS) and non-coding URR/DRR sequence components of TF genes using the optimized primers were further assayed through agarose gel-based EcoTILLING approach for allele mining.

To access the potential of EcoTILLING in large-scale mining and high-throughput genotyping of TF gene-derived SNP alleles, 192 *desi* and *kabuli* chickpea core/minicore germplasm accessions were selected (Table S1) from a 100-seed weight (SW) specific association panel (244 accessions) as constituted previously by Kujur et al. (2014). The high-quality genomic DNA isolated from these 192 accessions was quantified to equal concentration of 1 ng/μl. The bi-dimensional pooling of the uniformly quantified genomic DNA of 192 accessions was performed in two of each 96-well PCR plate to constitute eight micropools and one superpool (per plate) according to Tsai et al. (2011) (Figure 1). The genomic DNA of each of these pools was contrasted with that of ICC 4958 individually with a 1:1 ratio and further PCR amplified with the 1890 optimized primer-pairs designed from the 1133 TF genes (as per aforementioned methods). The amplified PCR product from each pool was denatured and renatured for homoduplex/heteroduplex formation and digested with CEL I-based SNiPerase-L enzyme (FRONTIER GENOMICS, Alaska, USA) following the detail instructions of manufacturer (FRONTIER GENOMICS; Figure 1). The purified CEL I cleaved homo/heteroduplex PCR products of each TF gene amplified from the pools were resolved in 2.5% agarose gel as per the EcoTILLING approach documented by Raghavan et al. (2007) (Figure 1). The individual accession exhibiting putative mutations (SNP allelic variants) was screened from the pools by accessing the digestion pattern of all 1133 TF genes in the row and/or column-wise de-multiplexed genomic DNA following the aforesaid agarose gel-based EcoTILLING method (Figures 2B,C). To ascertain the putative mutations (SNP allelic variants) discovered in the TF genes among accessions constituting the pools, the PCR products of corresponding genes amplified from the pools/accessions were sequenced by an automated 96 capillary ABI 3730xl DNA Analyzer (Applied Biosystems, USA) (Figure 2D). The SNP allelic variants were detected by aligning and comparing the multiple high-quality gene sequences among accessions following Kujur et al. (2013). The above-said analysis of allele mining and genotyping by agarose gel-based EcoTILLING led to discover 1133 SNP allelic variants from the diverse coding and non-coding regulatory sequence components of 1133 TF genes (Table S2). Of these, 406 (35.8%) and 702 (62.0%) SNP alleles exhibited synonymous and missense/nonsense non-synonymous amino acid substitutions, respectively in the CDS regions of 1108 TF genes. The remaining 25 (2.2%) SNP alleles were derived from the regulatory (URR/DRR) sequence components of 25 TF genes (Table S2). To determine the physical localization (bp) of SNPs on the chickpea genome, the 100-bp TF gene sequences flanking the 1133 SNP loci were BLAST searched (≥95% query coverage and percent identity) against the draft genome sequences of *desi* (Jain et al., 2013) and *kabuli* (Varshney et al., 2013a) chickpea. Notably, 1042 (92%) and 91 (8%) SNPs of the total discovered 1133 TF gene-derived SNP alleles were physically mapped on the eight chromosomes and unanchored scaffolds of *desi* and *kabuli* chickpea genomes, respectively (Table S2). These observations overall infer the efficacy of agarose gel-based EcoTILLING assay in large-scale mining and high-throughput genotyping of natural as well as functional allelic variants among diverse *desi* and *kabuli* chickpea germplasm accessions by the optimal expense of time, labor and cost in the research laboratories equipped with limited infrastructural facilities. Notably, this approach seems quite convenient and straightforward for screening the allelic variants more efficiently from the constituted pools containing DNA of numerous germplasm accessions (whole association panel) in a diploid crop species like chickpea with narrow genetic base and low intra-/inter-specific genetic polymorphism. Henceforth, this agarose-based detection assay has potential utility not only for the analysis of EcoTILLING using the pools of natural germplasm accessions but also for TILLING involving the pools of available EMS (ethyl methanesulfonate)-induced mutant lines (~10,000) of *desi* accession (ICC 4958; Varshney et al., 2009, 2013b) to identify the functionally relevant novel SNP allelic variants (mutations) influencing vital agronomic traits. Therefore, this optimized strategy has utility in accelerating the genomics-assisted crop improvement of chickpea through genetic/association mapping. In the present study, large-scale genotyping data of novel TF gene-based SNP alleles discovered from a seed weight association panel (192 accessions) using an optimized pool-based agarose gel-EcoTILLING strategy were assessed for trait association mapping potential to identify functional and natural allelic variants of the candidate TF genes regulating seed weight in chickpea.

## EcoTILLING-based association mapping delineates naturally occurring functional allelic variants of candidate genes regulating quantitative traits in chickpea

To perform candidate gene-based association analysis, the genotyping information of 1133 TF gene-derived SNP alleles (≥5% minor allele frequency) mined by EcoTILLING was integrated with multi-location replicated SW field phenotyping (100 seed weight: 6–63 g), principal component analysis (P), population genetic structure (Q), and kinship (K) matrix of 192 *desi* and *kabuli* accessions (association panel) of chickpea. At the most, we could expect clustering of 192 accessions into two distinct population groups at *K* = 2, in accordance with our preliminary genetic distance-based phylogenetic tree analysis. Using population genetic structure, the average likelihood value [Ln P(D)] against each K across 20 independent replications was estimated and plotted. The optimal value of K was determined following *ad hoc* and *delta K* procedures of Pritchard et al. (2000) and Evanno et al. (2005), respectively. At the optimum value of *K* = 2, the population structure model representing expected phylogenetic relationships among 192 accessions was constructed. The principal component analysis (PCA) among accessions was performed using GAPIT (Lipka et al., 2012). The kinship matrix (K) was estimated using SPAGeDi 1.2 (Hardy and Vekemans, 2002). For candidate gene-based association analysis, the CMLM (compressed mixed linear model) (P + K, K and Q + K) along with P3D (population parameters previously determined, Kang et al., 2010; Zhang et al., 2010) interfaces of GAPIT were employed following Kujur et al. (2013, 2014), Thudi et al. (2014) and Kumar et al. (2015). To ensure the accuracy and robustness of each SNP marker-trait association, the quantile-quantile plot-based false discovery rate (FDR cut-off ≤ 0.05) corrections (Benjamini and Hochberg, 1995) for multiple comparisons between observed/expected -log_10_(P)-values and adjusted *P*-value threshold of significance were performed in accordance with Kujur et al. (2015). The degree of association of SNP loci with SW trait was measured by the R^2^ (model with the SNP and adjusted *P*-value following FDR-controlling method). The TF gene-derived SNP loci exhibiting significant association with SW trait at lowest FDR adjusted *P*-values (threshold *P* < 10^−4^) and highest R^2^ were identified in chickpea.

The CMLM and P3D/EMMAX-based association analysis at a FDR cut-off ≤ 0.05 detected eight TF gene-derived SNPs exhibiting significant association with 100-seed weight at a *P* ≤ 10^−4^ (Table 1, Figure S1). Seven and one of these eight SW-associated SNPs were derived from the diverse coding (six non-synonymous and one synonymous SNP loci) and regulatory (URR) sequence components of eight TF genes, respectively. Seven SW-associated TF gene-based SNPs were physically mapped on four *desi* and *kabuli* chickpea chromosomes (1, 2, 3, and 4), whereas one SNP mapped on the unanchored scaffold of *desi* genome (Table 1, Figure S1). The proportion of SW phenotypic variation explained by eight SNP loci derived from eight TF genes [encoding *bZIP* (Basic-leucine zipper), *SBP* (Squamosa promoter binding protein) protein, Zinc finger-domain containing protein, NAC (No apical meristem arabidopsis transcription activation factor-cup shaped cotyledon), *bHLH* (Basic helix-loop-helix) protein, *AP2-EREBP* (APETALA-2/ethylene response element binding protein), *ARF* (auxin response factor), and *mTERF* (mitochondrial transcription termination factor)] among 192 *desi* and *kabuli* accessions belonging to an association panel varied from R^2^: 10 to 15% (Table 1, Figure S1). All significant eight SNP loci in combination explained 31% SW phenotypic variation. Strong association of one non-synonymous SNP in a *bZIP* TF gene (R^2^: 15% with P: 1.3 × 10^−5^) with SW was observed in *desi* and *kabuli* chickpea (Table 1, Figure S1). The SW-associated eight TF genes delineated by EcoTILLING-based allele mining, genotyping and trait association mapping in chickpea probably regulate seed growth and development, including determination of seed size/weight in many crop plants (Manning et al., 2006; Agarwal et al., 2007, 2011; Nijhawan et al., 2008; Libault et al., 2009; Wang et al., 2011, 2015; Heang and Sassa, 2012; Martínez-Andújar et al., 2012; Jones and Vodkin, 2013; Ha et al., 2014; Hudson and Hudson, 2015; Liu et al., 2015; Singh and Jain, 2015; Zhang et al., 2015). Especially, the seed weight trait association potential of four TFs (*bZIP, SBP, NAC*, and *bHLH*)-derived SNPs mapped on chromosomes 1 and 2, has been ascertained by recent studies in chickpea through identification of similar gene models-containing TFs, integrating seed weight trait-specific association analysis with QTL mapping, differential expression profiling and LD (linkage disequilibrium)-based marker haplotyping (Kujur et al., 2013, 2014). The validation of these TF gene-based SNPs in two of our independent studies suggests the potential significance and robustness of these identified novel functional molecular tags (natural allelic variants and genes) in controlling seed weight, which can essentially be deployed for marker-assisted genetic enhancement of chickpea.

**Table 1:** **Eight seed weight-associated SNP allelic variants of transcription factor genes delineated by EcoTILLING-based trait association mapping**.

**SNP IDs**	***Desi* and *Kabuli* chromosomes/scaffolds**	**SNP physical positions (bp)**	**SNPs**	**Gene accession IDs**	**Structural annotation**	**Putative functions**	**Association analysis**	
							**P**	**R^2^ (%)**
SNP0018^*^	*Ca_Kabuli_Chr01*	12794147	[G/T]	Ca_02472	CDS-Non Syn	Basic-leucine zipper (bZIP) protein	1.3 × 10^−5^	15
SNP0361^*^	*Ca_Kabuli_Chr01*	26026817	[C/T]	Ca_18591	CDS-Syn	Squamosa promoter binding protein (SBP)	2.1 × 10^−4^	10
SNP0748	*Ca_Desi_Scaffold_8872*	3778	[C/T]	Ca_20116	URR	Zinc finger-domain containing protein	2.3 × 10^−4^	10
SNP0777^*^	*Ca_Desi_Chr01*	16680465	[T/C]	Ca_TC09197	CDS-Non Syn	No apical meristem Arabidopsis transcription activation factor-cup shaped cotyledon (NAC)	1.7 × 10^−4^	12
SNP0790	*Ca_Desi_Chr01*	46718906	[C/G]	Ca_TC09680	CDS-Non Syn	mitochondrial transcription termination factor (mTERF)	1.9 × 10^−4^	11
SNP0804^*^	*Ca_Desi_Chr02*	10330226	[T/C]	Ca_TC11796	CDS-Non Syn	Basic helix-loop-helix (bHLH) protein	1.0 × 10^−5^	14
SNP0875	*Ca_Desi_Chr03*	39546588	[G/A]	Ca_TC17366	CDS-Non Syn	APETALA-2/ethylene response element binding protein (AP2-EREBP)	1.5 × 10^−4^	13
SNP0952	*Ca_Desi_Chr04*	48384848	[C/T]	Ca_TC03708	CDS-Non Syn	ARF (auxin response factor)	2.5 × 10^−4^	10

*CDS, coding DNA sequence; Syn, synonymous; and NonSyn, non-synonymous*.

* *Validated previously by seed weight QTL mapping*.

Collectively, the present study demonstrated the efficacy of an optimized pool-based agarose gel-EcoTILLING strategy (Figure 1) for high-throughput allele mining and genotyping as well as trait association analysis in a natural association panel to delineate novel functional allelic variants of the TF genes governing seed weight in chickpea. Therefore, this approach has potential utility to expedite various genomics-assisted breeding applications, including genetic enhancement targeting diverse qualitative and quantitative stress tolerance and yield component traits by optimal resource expenses in chickpea.

## Author contributions

DB, RS, and MN conducted experiments and drafted the manuscript. ST, CB, and HU helped in constitution of association panel and performed phenotyping. SP and AT conceived and designed the study, guided data analysis and interpretation, participated in drafting and correcting the manuscript critically and gave the final approval of the version to be published. All authors have read and approved the final manuscript.

### Conflict of interest statement

The authors declare that the research was conducted in the absence of any commercial or financial relationships that could be construed as a potential conflict of interest.
