# Supplementary material for: EcoTILLING-Based Association Mapping Efficiently Delineates Functionally Relevant Natural Allelic Variants of Candidate Genes Governing Agronomic Traits in Chickpea
Source: Front Plant Sci. 2016 Apr 19;7:450. doi: 10.3389/fpls.2016.00450 (PMC4835497; doi:10.3389/fpls.2016.00450)
Supplement: Supplementary file 1 [file Table1.PDF]

Table S1. 192 *desi* and *kabuli* *Cicer* accessions selected for large-scale SNP genotyping and seed weight trait association mapping using the agarose gel-based EcoTILLING assay in chickpea.

**Sl. No.**	**Accession No.**	**Cultivars**	**Geographical origin**	**Inferred population ancestry**
1	ICC3421	*Kabuli*	Israel	*Kabuli* (Population Group I)
2	ICC9402	*Kabuli*	Iran	*Kabuli* (Population Group I)
3	ICC13764	*Kabuli*	Iran	*Kabuli* (Population Group I)
4	ICC12037	*Kabuli*	Mexico	*Kabuli* (Population Group I)
5	ICC12492	*Kabuli*	India	*Kabuli* (Population Group I)
6	ICC13441	*Kabuli*	Iran	*Kabuli* (Population Group I)
7	ICC8855	*Kabuli*	Afghanistan	*Kabuli* (Population Group I)
8	ICC9862	*Kabuli*	Afghanistan	*Kabuli* (Population Group I)
9	ICC13461	*Kabuli*	Iran	*Kabuli* (Population Group I)
10	ICC13628	*Kabuli*	Iran	*Kabuli* (Population Group I)
11	ICC13523	*Kabuli*	Iran	*Kabuli* (Population Group I)
12	ICC13187	*Kabuli*	Iran	*Kabuli* (Population Group I)
13	ICC15802	*Kabuli*	Syrian Arab Republic	*Kabuli* (Population Group I)
14	ICC13077	*Kabuli*	India	*Kabuli* (Population Group I)
15	ICC5337	*Kabuli*	India	*Kabuli* (Population Group I)
16	ICC4841	*Kabuli*	Morocco	*Kabuli* (Population Group I)
17	ICC7571	*Kabuli*	Isreal	*Kabuli* (Population Group I)
18	ICC15264	*Kabuli*	Iran	*Kabuli* (Population Group I)
19	ICC7308	*Kabuli*	Peru	*Kabuli* (Population Group I)
20	ICC2277	*Kabuli*	Iran	*Kabuli* (Population Group I)
21	ICC6263	*Kabuli*	Union of Soviet Socialist Republics	*Kabuli* (Population Group I)
22	ICC8740	*Kabuli*	Afghanistan	*Kabuli* (Population Group I)
23	ICC13357	*Kabuli*	Iran	*Kabuli* (Population Group I)
24	ICC7255	*Kabuli*	India	*Kabuli* (Population Group I)
25	ICC7272	*Kabuli*	Algeria	*Kabuli* (Population Group I)
26	ICC15435	*Kabuli*	Morocco	*Kabuli* (Population Group I)
27	ICC11764	*Kabuli*	Chile	*Kabuli* (Population Group I)
28	ICC15697	*Kabuli*	Syria	*Kabuli* (Population Group I)
29	ICC13816	*Kabuli*	Union of Soviet Socialist Republics	*Kabuli* (Population Group I)
30	ICC7668	*Kabuli*	Union of Soviet Socialist Republics	*Kabuli* (Population Group I)

Sl. No.	Accession No.	Cultivars	Geographical origin	Inferred population ancestry
31	ICC13283	*Kabuli*	Iran	*Kabuli* (Population Group I)
32	ICC7295	*Kabuli*	Tunisia	*Kabuli* (Population Group I)
33	ICC12328	*Kabuli*	Cyprus	*Kabuli* (Population Group I)
34	ICC7315	*Kabuli*	Iran	*Kabuli* (Population Group I)
35	ICC11879	*Kabuli*	Turkey	*Kabuli* (Population Group I)
36	ICC14446	*Kabuli*	Italy	*Kabuli* (Population Group I)
37	ICC10755	*Kabuli*	Turkey	*Kabuli* (Population Group I)
38	ICC14190	*Kabuli*	India	*Kabuli* (Population Group I)
39	ICC15333	*Kabuli*	Iran	*Kabuli* (Population Group I)
40	ICC8042	*Kabuli*	Iran	*Kabuli* (Population Group I)
41	ICC8261	*Kabuli*	Turkey	*Kabuli* (Population Group I)
42	ICC10885	*Kabuli*	Ethiopia	*Kabuli* (Population Group I)
43	ICC8058	*Kabuli*	Iran	*Kabuli* (Population Group I)
44	ICC15512	*Kabuli*	Morocco	*Kabuli* (Population Group I)
45	ICC9137	*Kabuli*	Iran	*Kabuli* (Population Group I)
46	ICC15406	*Kabuli*	Morocco	*Kabuli* (Population Group I)
47	ICC10884	*Kabuli*	Ethiopia	*Kabuli* (Population Group I)
48	ICC15551	*Kabuli*	Australia	*Kabuli* (Population Group I)
49	ICC15725	*Kabuli*	Syrian Arab Republic	*Kabuli* (Population Group I)
50	ICC6204	*Kabuli*	Spain	*Kabuli* (Population Group I)
51	ICC6210	*Kabuli*	Spain	*Kabuli* (Population Group I)
52	ICC11847	*Kabuli*	Chile	*Kabuli* (Population Group I)
53	ICC7654	*Kabuli*	Turkey	*Kabuli* (Population Group I)
54	ICC11749	*Kabuli*	Chile	*Kabuli* (Population Group I)
55	ICC14199	*Kabuli*	Mexico	*Kabuli* (Population Group I)
56	ICC15518	*Kabuli*	Morocco	*Kabuli* (Population Group I)
57	ICC16814	*Kabuli*	Portugal	*Kabuli* (Population Group I)
58	ICC16796	*Kabuli*	Portugal	*Kabuli* (Population Group I)
59	ICC16811	*Kabuli*	India	*Kabuli* (Population Group I)
60	ICC6253	*Kabuli*	Morocco	*Kabuli* (Population Group I)
61	ICC11301	*Kabuli*	USA	*Kabuli* (Population Group I)
62	ICC10749	*Kabuli*	Turkey	*Kabuli* (Population Group I)

Sl. No.	Accession No.	Cultivars	Geographical origin	Inferred population ancestry
63	ICC14216	*Kabuli*	Mexico	*Kabuli* (Population Group I)
64	ICC14203	*Kabuli*	Mexico	*Kabuli* (Population Group I)
65	ICC11742	*Kabuli*	Chile	*Kabuli* (Population Group I)
66	ICC13821	*Kabuli*	Ethiopia	*Kabuli* (Population Group I)
67	ICC14462	*Kabuli*	USA	*Kabuli* (Population Group I)
68	ICC14220	*Kabuli*	Kenya	*Kabuli* (Population Group I)
69	ICC15944	*Kabuli*	USA	*Kabuli* (Population Group I)
70	ICC11303	*Kabuli*	Chile	*Kabuli* (Population Group I)
71	ICC12034	*Kabuli*	Mexico	*Kabuli* (Population Group I)
72	ICC7346	*Kabuli*	Mexico	*Kabuli* (Population Group I)
73	ICC15994	*Kabuli*	Spain	*Kabuli* (Population Group I)
74	ICC18591	*Kabuli*	Mexico	*Kabuli* (Population Group I)
75	ICC8155	*Kabuli*	USA	*Kabuli* (Population Group I)
76	ICC8151	*Kabuli*	USA	*Kabuli* (Population Group I)
77	ICC20268	*Kabuli*	India	*Kabuli* (Population Group I)
78	ICC9643	*Desi*	Afghanistan	*Desi* (Population Group II)
79	ICC9755	*Desi*	Afghanistan	*Desi* (Population Group II)
80	ICC2210	*Desi*	Algeria	*Desi* (Population Group II)
81	ICC12155	*Desi*	Bangladesh	*Desi* (Population Group II)
82	ICC3325	*Desi*	Cyprus	*Desi* (Population Group II)
83	ICC12824	*Desi*	Ethiopia	*Desi* (Population Group II)
84	ICC12537	*Desi*	Ethiopia	*Desi* (Population Group II)
85	ICC12654	*Desi*	Ethiopia	*Desi* (Population Group II)
86	ICC12726	*Desi*	Ethiopia	*Desi* (Population Group II)
87	ICC12866	*Desi*	Ethiopia	*Desi* (Population Group II)
88	ICC8607	*Desi*	Ethiopia	*Desi* (Population Group II)
89	ICC13863	*Desi*	Ethiopia	*Desi* (Population Group II)
90	ICC14077	*Desi*	Ethiopia	*Desi* (Population Group II)
91	ICC13892	*Desi*	Ethiopia	*Desi* (Population Group II)
92	ICC14051	*Desi*	Ethiopia	*Desi* (Population Group II)
93	ICC12851	*Desi*	Ethiopia	*Desi* (Population Group II)
94	ICC14098	*Desi*	Ethiopia	*Desi* (Population Group II)

Sl. No.	Accession No.	Cultivars	Geographical origin	Inferred population ancestry
95	ICC8621	*Desi*	Ethiopia	*Desi* (Population Group II)
96	ICCX-810800	*Desi*	India	*Desi* (Population Group II)
97	ICC5590	*Desi*	India	*Desi* (Population Group II)
98	ICC6013	*Desi*	India	*Desi* (Population Group II)
99	ICC5002	*Desi*	India	*Desi* (Population Group II)
100	ICC4926	*Desi*	India	*Desi* (Population Group II)
101	ICC4657	*Desi*	India	*Desi* (Population Group II)
102	ICC5878	*Desi*	India	*Desi* (Population Group II)
103	ICC456	*Desi*	India	*Desi* (Population Group II)
104	ICC5135	*Desi*	India	*Desi* (Population Group II)
105	ICC12916	*Desi*	India	*Desi* (Population Group II)
106	ICC440	*Desi*	India	*Desi* (Population Group II)
107	ICC2065	*Desi*	India	*Desi* (Population Group II)
108	ICC9586	*Desi*	India	*Desi* (Population Group II)
109	ICC9737	*Desi*	India	*Desi* (Population Group II)
110	ICC15618	*Desi*	India	*Desi* (Population Group II)
111	ICC14778	*Desi*	India	*Desi* (Population Group II)
112	ICC4567	*Desi*	India	*Desi* (Population Group II)
113	ICC637	*Desi*	India	*Desi* (Population Group II)
114	ICC1882	*Desi*	India	*Desi* (Population Group II)
115	ICC5639	*Desi*	India	*Desi* (Population Group II)
116	ICC8384	*Desi*	India	*Desi* (Population Group II)
117	ICC95	*Desi*	India	*Desi* (Population Group II)
118	ICC708	*Desi*	India	*Desi* (Population Group II)
119	ICC1205	*Desi*	India	*Desi* (Population Group II)
120	ICC14831	*Desi*	India	*Desi* (Population Group II)
121	ICC5434	*Desi*	India	*Desi* (Population Group II)
122	ICC10399	*Desi*	India	*Desi* (Population Group II)
123	ICC4593	*Desi*	India	*Desi* (Population Group II)
124	ICC11121	*Desi*	India	*Desi* (Population Group II)
125	ICC867	*Desi*	India	*Desi* (Population Group II)
126	ICC16915	*Desi*	India	*Desi* (Population Group II)

Sl. No.	Accession No.	Cultivars	Geographical origin	Inferred population ancestry
127	ICC14815	*Desi*	India	*Desi* (Population Group II)
128	ICC1356	*Desi*	India	*Desi* (Population Group II)
129	ICC1422	*Desi*	India	*Desi* (Population Group II)
130	ICC5613	*Desi*	India	*Desi* (Population Group II)
131	ICC14799	*Desi*	India	*Desi* (Population Group II)
132	ICC1194	*Desi*	India	*Desi* (Population Group II)
133	ICC15567	*Desi*	India	*Desi* (Population Group II)
134	ICC506	*Desi*	India	*Desi* (Population Group II)
135	ICC4639	*Desi*	India	*Desi* (Population Group II)
136	ICC283	*Desi*	India	*Desi* (Population Group II)
137	ICC1180	*Desi*	India	*Desi* (Population Group II)
138	ICC1397	*Desi*	India	*Desi* (Population Group II)
139	ICC14669	*Desi*	India	*Desi* (Population Group II)
140	ICC1510	*Desi*	India	*Desi* (Population Group II)
141	ICC4533	*Desi*	India	*Desi* (Population Group II)
142	ICC16903	*Desi*	India	*Desi* (Population Group II)
143	ICC1230	*Desi*	India	*Desi* (Population Group II)
144	ICC8318	*Desi*	India	*Desi* (Population Group II)
145	ICC5383	*Desi*	India	*Desi* (Population Group II)
146	ICC15610	*Desi*	India	*Desi* (Population Group II)
147	ICC1398	*Desi*	India	*Desi* (Population Group II)
148	ICC14595	*Desi*	India	*Desi* (Population Group II)
149	ICC4918	*Desi*	India	*Desi* (Population Group II)
150	ICC3362	*Desi*	Iran	*Desi* (Population Group II)
151	ICC3946	*Desi*	Iran	*Desi* (Population Group II)
152	ICC3761	*Desi*	Iran	*Desi* (Population Group II)
153	ICC4814	*Desi*	Iran	*Desi* (Population Group II)
154	ICC3776	*Desi*	Iran	*Desi* (Population Group II)
155	ICC4418	*Desi*	Iran	*Desi* (Population Group II)
156	ICC4463	*Desi*	Iran	*Desi* (Population Group II)
157	ICC6802	*Desi*	Iran	*Desi* (Population Group II)
158	ICC13219	*Desi*	Iran	*Desi* (Population Group II)

Sl. No.	Accession No.	Cultivars	Geographical origin	Inferred population ancestry
159	ICC2969	*Desi*	Iran	*Desi* (Population Group II)
160	ICC2580	*Desi*	Iran	*Desi* (Population Group II)
161	ICC3512	*Desi*	Iran	*Desi* (Population Group II)
162	ICC2990	*Desi*	Iran	*Desi* (Population Group II)
163	ICC7867	*Desi*	Iran	*Desi* (Population Group II)
164	ICC2919	*Desi*	Iran	*Desi* (Population Group II)
165	ICC13599	*Desi*	Iran	*Desi* (Population Group II)
166	ICC7554	*Desi*	Iran	*Desi* (Population Group II)
167	ICC15294	*Desi*	Iran	*Desi* (Population Group II)
168	ICC7819	*Desi*	Iran	*Desi* (Population Group II)
169	ICC6877	*Desi*	Iran	*Desi* (Population Group II)
170	ICC6293	*Desi*	Italy	*Desi* (Population Group II)
171	ICC8522	*Desi*	Italy	*Desi* (Population Group II)
172	ICC16261	*Desi*	Malawi	*Desi* (Population Group II)
173	ICC16269	*Desi*	Malawi	*Desi* (Population Group II)
174	ICC16374	*Desi*	Malawi	*Desi* (Population Group II)
175	ICC12028	*Desi*	Mexico	*Desi* (Population Group II)
176	ICC5504	*Desi*	Mexico	*Desi* (Population Group II)
177	ICC15510	*Desi*	Morocco	*Desi* (Population Group II)
178	ICC16207	*Desi*	Myanmar	*Desi* (Population Group II)
179	ICC12307	*Desi*	Myanmar	*Desi* (Population Group II)
180	ICC12299	*Desi*	Nepal	*Desi* (Population Group II)
181	ICC11944	*Desi*	Nepal	*Desi* (Population Group II)
182	ICC1164	*Desi*	Nigeria	*Desi* (Population Group II)
183	ICC1052	*Desi*	Pakistan	*Desi* (Population Group II)
184	ICC16487	*Desi*	Pakistan	*Desi* (Population Group II)
185	ICC1161	*Desi*	Pakistan	*Desi* (Population Group II)
186	ICC8195	*Desi*	Pakistan	*Desi* (Population Group II)
187	ICC16524	*Desi*	Pakistan	*Desi* (Population Group II)
188	ICC15612	*Desi*	Tanzania	*Desi* (Population Group II)
189	ICC7184	*Desi*	Turkey	*Desi* (Population Group II)
190	ICC4495	*Desi*	Turkey	*Desi* (Population Group II)

Sl. No.	Accession No.	Cultivars	Geographical origin	Inferred population ancestry
191	ICC11284	*Desi*	Union of Soviet Socialist Republics	*Desi* (Population Group II)
192	ICC6306	*Desi*	Union of Soviet Socialist Republics	*Desi* (Population Group II)
