# Supplementary material for: EcoTILLING-Based Association Mapping Efficiently Delineates Functionally Relevant Natural Allelic Variants of Candidate Genes Governing Agronomic Traits in Chickpea
Source: Front Plant Sci. 2016 Apr 19;7:450. doi: 10.3389/fpls.2016.00450 (PMC4835497; doi:10.3389/fpls.2016.00450)
Supplement: Supplementary file 2 [file Table2.PDF]

Table S2. Details regarding 1133 transcription factor genes selected for large-scale mining and genotyping of SNP alleles and genetic association analysis using agarose gel-based EcoTILLING assay in chickpea.

**SNP IDs**	***Kabuli* /*desi* gene accession IDs**	***Kabuli* /*desi* chromosomes**	**SNPs**	**Physical positions (bp)**	**Sequence components of genome**	**Putative functions**	**Forward primer sequences (5'-3')**	**Reverse primer sequences (5'-3')**	**Primers used in Figure 2A**
SNP0001	Ca_00075	*Ca_Kabuli_Chr01*	[C/T]	644318	CDS_NON_SYNONYMOUS	Protein of unknown function DUF639	CCTCACTTCATTTTCAAAGGTTCT	GGATGTCTTCTCCTAAGGCAGTTA	NA
SNP0002	Ca_00186	*Ca_Kabuli_Chr01*	[G/A]	1533723	CDS_NON_SYNONYMOUS	Helix-loop-helix DNA-binding domain	GCAAGTTCCCTAGAATTGTTCTGT	GTTCCTCCTCAAGAACATTCACTT	NA
SNP0003	Ca_00224	*Ca_Kabuli_Chr01*	[C/A]	1783923	CDS_NON_SYNONYMOUS	Zinc finger, RING-type	GTCTTTTTATTCCAAAGCTGCAAG	TATTCGTGTAACCGAACAAAGATG	NA
SNP0004	Ca_00244	*Ca_Kabuli_Chr01*	[A/G]	1962876	CDS_NON_SYNONYMOUS	Transcriptional factor B3	TCCTTCCATATATAGGATGCGATT	CTAAATGGCTTCAACCTACAACCT	NA
SNP0005	Ca_00274	*Ca_Kabuli_Chr01*	[T/C]	2188472	CDS_NON_SYNONYMOUS	SNF2-related	AGAGTGAAAGGAGAGAAAAGGTGA	CTCTGTCTATGGCTTGATCTTCAG	NA
SNP0006	Ca_00347	*Ca_Kabuli_Chr01*	[G/T]	2832939	CDS_NON_SYNONYMOUS	Transcriptional factor B3	ATCTCAGGTTCAAACCTTAACCAC	AGGAAATATCAGAATGCCACAAGT	NA
SNP0007	Ca_00512	*Ca_Kabuli_Chr01*	[G/C]	4302669	CDS_NON_SYNONYMOUS	Zinc finger, CCCH-type	GTTGCATATGCTTGAAATCTCTTG	CTTTTGATTGTCACAACATCACTG	NA
SNP0008	Ca_00562	*Ca_Kabuli_Chr01*	[C/T]	4780330	CDS_NON_SYNONYMOUS	Zinc finger, RING-type	GTCCAAGGGACATTATTCAAAAAC	CAAGAGGTCTCTAGGAGGGAAAA	NA
SNP0009	Ca_00613	*Ca_Kabuli_Chr01*	[G/A]	5249839	CDS_NON_SYNONYMOUS	Protein of unknown function DUF584	AAGTGCTTCACGTCATGATTGTAT	TGAGTTGGACTCTCTTCAGACAAG	NA
SNP0010	Ca_00628	*Ca_Kabuli_Chr01*	[G/A]	5430809	CDS_NON_SYNONYMOUS	Protein of unknown function DUF584	ATGTTTCATTTGGGTTCTCTCTTC	TCACCTGAACAAACAGAAAGACAC	NA
SNP0011	Ca_00656	*Ca_Kabuli_Chr01*	[C/A]	5666217	CDS_NON_SYNONYMOUS	DNA-binding WRKY	TGGTTACTATCTCCGTTGTTTTCA	AGTTCGCAAACAAGTCAGTTTTCT	NA
SNP0012	Ca_07932	*Ca_Kabuli_Chr01*	[A/G]	6679329	CDS_NON_SYNONYMOUS	WD40 repeat	TTTTAGAGACCACCTCAACAAGTG	TGGAATGACTCTGTCATAGCTGTT	NA
SNP0013	Ca_07963	*Ca_Kabuli_Chr01*	[G/T]	6952382	CDS_NON_SYNONYMOUS	Protein of unknown function DUF1296	TGAAGTGACAGCAAACATAACAAG	ATACTTACCCTTCCCTGCTACACA	NA
SNP0014	Ca_07989	*Ca_Kabuli_Chr01*	[T/A]	7325164	CDS_NON_SYNONYMOUS	WD40 repeat	GCACCGCTCAAGACAATAATAATAA	AAACAAACTCACACTACACCAAGAA	NA
SNP0015	Ca_08067	*Ca_Kabuli_Chr01*	[T/C]	8192580	CDS_NON_SYNONYMOUS	Zinc finger, CCHC-type	TTACATAGCTGTGCTTGGTCATTC	AGGTCATTTATCTCGGGATTGTAA	NA
SNP0016	Ca_02909	*Ca_Kabuli_Chr01*	[T/C]	8942131	CDS_NON_SYNONYMOUS	Zinc finger, UBP-type	ATCTTTTGAAAGAGACATCCCAAC	TCGAAGAGAGGTATGCTTTTATCC	NA
SNP0017	Ca_02625	*Ca_Kabuli_Chr01*	[C/A]	11285291	CDS_NON_SYNONYMOUS	Transcription factor GRAS	AGCATCCAGTTGTTATCTTCATCA	AAACTCAGCTTTTTACTGGTTTGG	NA
**SNP0018**	**Ca_02472**	***Ca_Kabuli_Chr01***	**[G/T]**	**12794147**	**CDS_NON_SYNONYMOUS**	**Basic-leucine zipper (bZIP) transcription factor**	**TTCGTGAAACAGTTAAAACTCCTC**	**CTTAAAGGAAAGTGATTTAGGTGGT**	NA
SNP0019	Ca_02459	*Ca_Kabuli_Chr01*	[G/A]	12875506	CDS_NON_SYNONYMOUS	Zinc finger, C2H2-type	CGGATCAACAAACATAAACAACTC	CACAAAACCTCTCACTCCCTATTC	NA
SNP0020	Ca_14071	*Ca_Kabuli_Chr01*	[G/A]	13076695	CDS_NON_SYNONYMOUS	Zinc finger, RING-type	TGACGAATCAATAGAAGAACTTGC	TAGTCGCGAAGGCCTTATTACTAT	NA
SNP0021	Ca_14138	*Ca_Kabuli_Chr01*	[C/A]	13762410	CDS_NON_SYNONYMOUS	SET domain	CTTATATTTTGAGACGGAGGGAGT	CAGAAACTCCACAACAAAATCATC	NA
SNP0022	Ca_06945	*Ca_Kabuli_Chr01*	[A/C]	16061536	CDS_NON_SYNONYMOUS	BTB/Kelch-associated	TATCAACTACCCACCAAAAGGATT	AGTTGATTTTGGGTTTGCTCTAGT	NA
SNP0023	Ca_22780	*Ca_Kabuli_Chr01*	[A/G]	17314911	CDS_NON_SYNONYMOUS	WD40 repeat	CTTCCATATCTGTTCAAGCCTTTT	CTATCTCATGAGTTGGTTGGTCTG	NA
SNP0024	Ca_22785	*Ca_Kabuli_Chr01*	[C/A]	17395543	CDS_NON_SYNONYMOUS	Domain of unknown function DUF625	CATGTTAACCAAGCCAGAGAAAAT	CCTCCAAGAATACAGGTTGACTTT	NA
SNP0025	Ca_21411	*Ca_Kabuli_Chr01*	[C/A]	34866709	CDS_NON_SYNONYMOUS	SANT domain, DNA binding	GAAGTAAGCTCTCCAAAATTGTCC	ACGCACCACTATCTAAACCAGATT	NA
SNP0026	Ca_13620	*Ca_Kabuli_Chr01*	[G/A]	42452801	CDS_NON_SYNONYMOUS	Transcription factor CBF/NF-Y/archaeal histone	TGAGTTGGGTGGTGAGAAGAATAC	TAACAAGAGCCTCAATCACTTCTG	NA

SNP IDs	*Kabuli* /*desi* gene accession IDs	*Kabuli* /*desi* chromosomes	SNPs	Physical positions (bp)	Sequence components of genome	Putative functions	Forward primer sequences (5'-3')	Reverse primer sequences (5'-3')	Primers used in Figure 2A
SNP0027	Ca_13610	*Ca_Kabuli_Chr01*	[T/G]	42605239	CDS_NON_SYNONYMOUS	Helix-loop-helix DNA-binding domain	AGATGAGAGCCATATCTTGGAAAC	CAGTCTTTTTCTTCCTTCCTTCAA	NA
SNP0028	Ca_13608	*Ca_Kabuli_Chr01*	[T/G]	42613060	CDS_NON_SYNONYMOUS	Helix-loop-helix DNA-binding domain	GACCTTCCTTATCTTCCTGCAATA	CAGTCTTTTTCTTCCTTCCTTCAA	NA
SNP0029	Ca_12938	*Ca_Kabuli_Chr01*	[T/A]	47042798	CDS_NON_SYNONYMOUS	Protein of unknown function DUF92, TMEM19	TACCCTGCAAACACTTTACTTTCA	CAGGTCCCTTATATTGCTGAACAT	NA
SNP0030	Ca_12602	*Ca_Kabuli_Chr02*	[G/A]	1828406	CDS_NON_SYNONYMOUS	Protein of unknown function DUF1421	TCAACTACATCGTCGATAGAAACC	CAAGGGCTGAATTGTTTAATCATC	NA
SNP0031	Ca_10563	*Ca_Kabuli_Chr02*	[G/A]	2783727	CDS_NON_SYNONYMOUS	Zinc finger, RING-type	TAAGGGAGGGTGTTTTAGATGAAG	GGCCTTAAATTTCTTGATTCTCTG	NA
SNP0032	Ca_10517	*Ca_Kabuli_Chr02*	[C/T]	3366023	CDS_NON_SYNONYMOUS	ZF-HD homeobox protein, Cys/His-rich dimerisation domain	GCAAGTGGAGTTTGGTATAGTGTG	ATCATCTTTTGTGGAGTCTCTCCT	NA
SNP0033	Ca_10515	*Ca_Kabuli_Chr02*	[C/T]	3410375	CDS_NON_SYNONYMOUS	ZF-HD homeobox protein, Cys/His-rich dimerisation domain	AAAGGCAAGTGGAGTTTGGTATAG	TAATGCCTCTTGAGAAAGTGGAGT	NA
SNP0034	Ca_10463	*Ca_Kabuli_Chr02*	[A/G]	3930211	CDS_NON_SYNONYMOUS	Zinc finger, PHD-type	TCTTCTTGATTGAATCTCTCGTCA	TGTTAGCTTTCGTGTTCAGTTAGG	NA
SNP0035	Ca_14702	*Ca_Kabuli_Chr02*	[C/T]	5079639	CDS_NON_SYNONYMOUS	Zinc finger, RING-type	TCTTTGAACAAATCAGGAAGACTG	TTAGGATCGCTCAAACATATAGGG	NA
SNP0036	Ca_20380	*Ca_Kabuli_Chr02*	[A/G]	10068214	CDS_NON_SYNONYMOUS	SANT domain, DNA binding	CTTTTGTCTATACTTGCAGCAGGA	AAACTGTCTCCATATCAGACACCA	NA
SNP0037	Ca_17564	*Ca_Kabuli_Chr02*	[G/A]	10760939	CDS_NON_SYNONYMOUS	Zinc finger, C2H2-type	TCAGTCATGAGTACAATGAGTTTAAGG	CATCAAATCGACACATTCACAGTA	NA
SNP0038	Ca_18061	*Ca_Kabuli_Chr02*	[A/C]	12281599	CDS_NON_SYNONYMOUS	Zinc finger, RING-CH-type	GTCAATATCTCGCACAACAACAAT	TGGAAGTACGAAGTTGGTAATGAA	NA
SNP0039	Ca_18079	*Ca_Kabuli_Chr02*	[C/G]	12528825	CDS_NON_SYNONYMOUS	Zinc finger, Dof-type	ACTTTACTTACTTGCTGGCCAAAC	GCTTGCCATTGAATGTCTAAATCT	NA
SNP0040	Ca_11686	*Ca_Kabuli_Chr02*	[T/G]	13079417	CDS_NON_SYNONYMOUS	Protein of unknown function DUF3110	CTGACCCGACTAACAAGCCTATAC	CACTTGATCGAAGTTAATCTGTCA	NA
SNP0041	Ca_14967	*Ca_Kabuli_Chr02*	[G/A]	23297412	CDS_NON_SYNONYMOUS	Domain of unknown function FMP27, domain-6, C-terminal	AAAATACGTCCAAAACAGGAGAGA	GCACCTGGTAAAGTACAAACAACA	NA
SNP0042	Ca_21663	*Ca_Kabuli_Chr02*	[T/C]	24291665	CDS_NON_SYNONYMOUS	Protein of unknown function DUF3133	TTGATTTCCTTCTCCTTCTAATGG	GTTCTTATCTTATGCAGGCAGTGA	NA
SNP0043	Ca_14236	*Ca_Kabuli_Chr02*	[A/G]	25022971	CDS_NON_SYNONYMOUS	Zinc finger, B-box	TGTTTCCACACTGTTGATCCTAGT	ATCAAATTGGCTACTATGCACTGA	NA
SNP0044	Ca_14280	*Ca_Kabuli_Chr02*	[C/G]	25507637	CDS_NON_SYNONYMOUS	Zinc finger, CCCH-type	CATTGCTGTTTGTGATTCTGTAAC	AGTGTTAAACCTGCTTTTTGGAAG	NA
SNP0045	Ca_15694	*Ca_Kabuli_Chr02*	[T/C]	28018158	CDS_NON_SYNONYMOUS	AUX/IAA protein	AGACCAGATGAATCGATATTGACC	ATCCTGGCACTTCTTCTTTTCTT	NA
SNP0046	Ca_12487	*Ca_Kabuli_Chr02*	[G/A]	30702090	CDS_NON_SYNONYMOUS	High mobility group, HMG1/HMG2	CCCCCTCTTATCATTGTTCTTTTA	GGCACTGTCAGTTCTCTCTTTTTC	NA
SNP0047	Ca_16883	*Ca_Kabuli_Chr02*	[T/C]	34848480	CDS_NON_SYNONYMOUS	Bromodomain	GATATCCATTTCATCCCATCCTAA	ACTCTAGGACCTACTGCTTGGAGA	NA
SNP0048	Ca_09731	*Ca_Kabuli_Chr02*	[T/C]	35612895	CDS_NON_SYNONYMOUS	Protein of unknown function DUF827, plant	GCTGACTGAGACAATGAATGAAAT	CAATTGCTTCAAGAATGTCTCTCT	NA
SNP0049	Ca_09786	*Ca_Kabuli_Chr02*	[C/T]	36088623	CDS_NON_SYNONYMOUS	Transcription factor GRAS	CAATTCTCCACTCAAAGTTCCTCT	AAGCAACCAAATCTCTATTCATCC	NA
SNP0050	Ca_22148	*Ca_Kabuli_Chr03*	[C/T]	2708703	CDS_NON_SYNONYMOUS	Zinc finger, B-box	CATGATACTTTGGCCCTTAGATTC	CAAAAACAGCAAGAACAATCTCTG	NA
SNP0051	Ca_21346	*Ca_Kabuli_Chr03*	[G/A]	3617555	CDS_NON_SYNONYMOUS	ZF-HD homeobox protein, Cys/His-rich dimerisation domain	AAGTGGCAGAAAACTCTTAACACC	GAAGTCCTGTGAGATCATCATTTG	NA
SNP0052	Ca_16540	*Ca_Kabuli_Chr03*	[T/G]	7015213	CDS_NON_SYNONYMOUS	Zinc finger, ZZ-type	TATGATGCTGTTCCAAACCATTAC	GTTTTAGCTTGAAGGGGAGTGTTA	NA
SNP0053	Ca_22696	*Ca_Kabuli_Chr03*	[A/G]	13420756	CDS_NON_SYNONYMOUS	Zinc finger, C2H2-type	GCTTTTCTGGGACTTGGATAATAA	CACCTTTTTCTTTCCCTTGTACTC	NA

SNP IDs	*Kabuli* /*desi* gene accession IDs	*Kabuli* /*desi* chromosomes	SNPs	Physical positions (bp)	Sequence components of genome	Putative functions	Forward primer sequences (5'-3')	Reverse primer sequences (5'-3')	Primers used in Figure 2A
SNP0054	Ca_24617	*Ca_Kabuli_Chr03*	[A/G]	17181213	CDS_NON_SYNONYMOUS	WD40 repeat	GTACACCCAAACCAGGTCTAAGTT	TTTTGAGTGGTGGTCTGTATTTTG	NA
SNP0055	Ca_09470	*Ca_Kabuli_Chr03*	[A/G]	21020182	CDS_NON_SYNONYMOUS	Protein of unknown function DUF827, plant	TGTTATTTTGAGTGCTAAGGTTGC	GCTTCGTCTACCCTTTTCAAAATA	NA
SNP0056	Ca_06206	*Ca_Kabuli_Chr03*	[A/G]	23028855	CDS_NON_SYNONYMOUS	Mediator complex, subunit Med12	GGAAGGAATCTCATGAGTAACTGG	GGCTATTAAGGTGGACAGTTCAGT	NA
SNP0057	Ca_06189	*Ca_Kabuli_Chr03*	[C/G]	23273069	CDS_NON_SYNONYMOUS	SNF2-related	GTTGTGGATTGTAACCAAACAAGA	CACCTAAGTTCATGTTTGCAGAAG	NA
SNP0058	Ca_08144	*Ca_Kabuli_Chr03*	[G/A]	27632100	CDS_NON_SYNONYMOUS	Myb, DNA-binding	GGTGTACCTGGACTGACCATTTAT	ACCTTAGTATGCATTATCGCTCTG	NA
SNP0059	Ca_08113	*Ca_Kabuli_Chr03*	[T/G]	27968979	CDS_NON_SYNONYMOUS	Zinc finger, C2H2-type	AAATCTCTTTGTCTTCATCAGCAG	TGAAACCTACATAGATTCATCCTCA	NA
SNP0060	Ca_07170	*Ca_Kabuli_Chr03*	[A/T]	28614919	CDS_NON_SYNONYMOUS	Basic-leucine zipper (bZIP) transcription factor	ACCACAAACCTCTCCAAGTAGAAC	AGAGATATCTGCTTTTTGCTTTGG	NA
SNP0061	Ca_12181	*Ca_Kabuli_Chr03*	[T/C]	30837265	CDS_NON_SYNONYMOUS	Domain of unknown function DUF828	ATCTTAAGCCAACCTCATGCTAAC	TTCCATACACACGCTTAGACATTT	NA
SNP0062	Ca_00729	*Ca_Kabuli_Chr03*	[G/A]	34576769	CDS_NON_SYNONYMOUS	Transcription factor, MADS-box	CAGCAATCTCTTTGGAATTTCTTC	GGTTGTGATTCACTGTTTGTGATT	NA
SNP0063	Ca_00732	*Ca_Kabuli_Chr03*	[T/A]	34603669	CDS_NON_SYNONYMOUS	Zinc finger, PHD-type	TGAAGTCATAGAAAGCCATCAATC	GCTCAAATTCTTTATGGAATCCTC	NA
SNP0064	Ca_00756	*Ca_Kabuli_Chr03*	[G/A]	34800277	CDS_NON_SYNONYMOUS	Protein of unknown function DUF668	TAAAGAAGAATCTGATGTCCACGA	CATGTCAGTTTCAAACAATTCTCC	NA
SNP0065	Ca_00917	*Ca_Kabuli_Chr03*	[C/G]	36165997	CDS_NON_SYNONYMOUS	Zinc finger, C2H2-type matrin	AATGCAGGTTACAAACACAAGGTA	CAAGTTCTTTCATCCAGGAATAGC	NA
SNP0066	Ca_00938	*Ca_Kabuli_Chr03*	[T/G]	36282091	CDS_NON_SYNONYMOUS	Domain of unknown function DUF255	ACCCAGGTAAAGATTAATGTCTGG	CAAGAGAGGTTGTGGTTAAAAGGT	NA
SNP0067	Ca_00942	*Ca_Kabuli_Chr03*	[A/G]	36330750	CDS_NON_SYNONYMOUS	Homeobox	ACAATATGGCTTACTTTGCTGACA	CACTGGACCTGGAAAATAAGAATC	NA
SNP0068	Ca_00943	*Ca_Kabuli_Chr03*	[T/C]	36345158	CDS_NON_SYNONYMOUS	Zinc finger, C2H2-type	GTTAGCTCATGGAAAAGTGCATAA	CGGAGACGTAGACATATTTGATTG	NA
SNP0069	Ca_01036	*Ca_Kabuli_Chr03*	[T/C]	37094437	CDS_NON_SYNONYMOUS	Transcription factor, SBP-box	CATTCATCTCTTGTGGAATCAATC	TCCTGCTTAATGGAACAGTGAGTA	NA
SNP0070	Ca_01102	*Ca_Kabuli_Chr03*	[G/A]	37635909	CDS_NON_SYNONYMOUS	Zinc finger, U1-type	TTTACTGAGCAAGGTAAGGGAGTT	TGCTTCTATATGGTTTGGCTATCA	NA
SNP0071	Ca_01189	*Ca_Kabuli_Chr03*	[T/C]	38386392	CDS_NON_SYNONYMOUS	Protein of unknown function DUF3595	TCATTGAACTGCTACACTCATTCA	CACTAAAACCCAATATCTGCCTCT	NA
SNP0072	Ca_01258	*Ca_Kabuli_Chr03*	[T/A]	38965610	CDS_NON_SYNONYMOUS	SANT domain, DNA binding	AGGAGAAAAAGGAGAGGAATTGAT	TGCAGGGTAATAATGGGATAAACT	NA
SNP0073	Ca_01271	*Ca_Kabuli_Chr03*	[T/A]	39084916	CDS_NON_SYNONYMOUS	Domain of unknown function DUF296	TCAATGGCAGTAGAAAGCATTAAC	CTAACAAATGGCAAGAGGTTTGAT	NA
SNP0074	Ca_07683	*Ca_Kabuli_Chr04*	[C/A]	504928	CDS_NON_SYNONYMOUS	SANT associated	GGTTAGTTGGGCTGAGATGATTAG	CATAATGTTGAAGTCAGCCAGAAG	NA
SNP0075	Ca_07798	*Ca_Kabuli_Chr04*	[A/T]	1747181	CDS_NON_SYNONYMOUS	Transcription factor jumonji/aspartyl beta-hydroxylase	GCCGCTTCTTTCAATTTTATACTC	TACGTTGGTTAGTATTGGCTGAGA	NA
SNP0076	Ca_07804	*Ca_Kabuli_Chr04*	[C/T]	1833725	CDS_NON_SYNONYMOUS	Domain of unknown function DUF828	CTCTTGATTCTGTCTTCAATCCAA	GAAAAATCACAGAACACAGATTGC	NA
SNP0077	Ca_07844	*Ca_Kabuli_Chr04*	[G/A]	2213710	CDS_NON_SYNONYMOUS	Helix-loop-helix DNA-binding domain	TTGGGAAGGTACGGTTAATGTAGT	TACATAAGCTTGACTACCCTGCAA	NA
SNP0078	Ca_03781	*Ca_Kabuli_Chr04*	[G/A]	4757416	CDS_NON_SYNONYMOUS	SET domain	CAAACCTTGTGAAGAGTAAGCAAA	ATGGAGAAAGACCAAAACAGAAAC	NA
SNP0079	Ca_03778	*Ca_Kabuli_Chr04*	[C/T]	4799116	CDS_NON_SYNONYMOUS	SNF2-related	AACAAAGACCTCCTTGAAAATGTC	TGTGCCTAAATGTATCCAGTGTTT	NA
SNP0080	Ca_03766	*Ca_Kabuli_Chr04*	[T/C]	4920706	CDS_NON_SYNONYMOUS	Zinc finger, PMZ-type	ATGAGAGTCATACATGTGGAGGAA	CTAAAGTGACCTGTTTGATTGCAG	NA

SNP IDs	*Kabuli*/*desi* gene accession IDs	*Kabuli*/*desi* chromosomes	SNPs	Physical positions (bp)	Sequence components of genome	Putative functions	Forward primer sequences (5'-3')	Reverse primer sequences (5'-3')	Primers used in Figure 2A
SNP0081	Ca_03621	*Ca_Kabuli_Chr04*	[T/A]	6238367	CDS_NON_SYNONYMOUS	High mobility group, HMG1/HMG2	ACTGTGGCCCTATAGTTGATTCAG	CGTTTTCAAGATAAGCCCAAATAC	NA
SNP0082	Ca_03564	*Ca_Kabuli_Chr04*	[T/C]	6765884	CDS_NON_SYNONYMOUS	SANT domain, DNA binding	TGATGCTGTCCTTTTCTTTGTAAG	ATGAACAGCAAAGACATGTTCCTA	NA
SNP0083	Ca_03548	*Ca_Kabuli_Chr04*	[C/T]	6919896	CDS_NON_SYNONYMOUS	Transcription factor, SBP-box	TGAGTAGCAGTTTCTTTCCTTTCA	ACTTTTAAACAGAAGTGCCACACA	NA
SNP0084	Ca_03516	*Ca_Kabuli_Chr04*	[G/A]	7185974	CDS_NON_SYNONYMOUS	SANT domain, DNA binding	ATGATGTTGTTTCGGTGATTAGTG	AGACACCTAAAGTTGGTTGGAAAG	NA
SNP0085	Ca_03429	*Ca_Kabuli_Chr04*	[T/C]	8050770	CDS_NON_SYNONYMOUS	Tubby, C-terminal	GTGATACCTAGAGCCCAAAACATT	GGTACCATTTGTTACCACCATTTT	NA
SNP0086	Ca_08309	*Ca_Kabuli_Chr04*	[T/G]	8198702	CDS_NON_SYNONYMOUS	WD40 repeat	ATGTTTACTCGACTCCATTGCATA	ACAGTAAGAACGATAAAGCGAAGG	NA
SNP0087	Ca_08340	*Ca_Kabuli_Chr04*	[G/A]	8484976	CDS_NON_SYNONYMOUS	WD40 repeat	AAAAGAGGGAAGAAAGAACCAGAT	CACTTCTTTTTGGCAATTACACTG	NA
SNP0088	Ca_08371	*Ca_Kabuli_Chr04*	[A/C]	8751618	CDS_NON_SYNONYMOUS	No apical meristem (NAM) protein	ACCTGAATGCCAAACCATCTATAC	GAAGAAAGCCCTTACTAACTGCAA	NA
SNP0089	Ca_08381	*Ca_Kabuli_Chr04*	[T/C]	8848653	CDS_NON_SYNONYMOUS	Zinc finger, RING-type	TGAGACTATTAGTTGGCAAGAAGC	CCAGACAGAATTCAAAACCAGTAA	NA
SNP0090	Ca_05487	*Ca_Kabuli_Chr04*	[C/T]	16278600	CDS_NON_SYNONYMOUS	Zinc finger, B-box	CGTTTTTCTCTGTACCTTGAAACC	AAAACCGCTAGAGCTTGTGATAAT	NA
SNP0091	Ca_05477	*Ca_Kabuli_Chr04*	[T/A]	16501291	CDS_NON_SYNONYMOUS	Domain of unknown function DUF292, eukaryotic	GTTCTGATGTCCTTCAACTTCTCC	ATTCACCCTCAATAGCAACAGAAT	NA
SNP0092	Ca_05430	*Ca_Kabuli_Chr04*	[A/T]	16926614	CDS_NON_SYNONYMOUS	Zinc finger, C2H2-type	CATGAGAAATTATATGGTCCATGC	AACAACCTCTTCAACATTCCTTTC	NA
SNP0093	Ca_18659	*Ca_Kabuli_Chr04*	[T/C]	18222762	CDS_NON_SYNONYMOUS	SET domain	TCAGTGGCATTTGACAATTT	TGCCCTCTCCTAGTTTATGTTTCT	NA
SNP0094	Ca_20151	*Ca_Kabuli_Chr04*	[C/T]	21830534	CDS_NON_SYNONYMOUS	Helix-turn-helix motif, lambda-like repressor	CTCTTTCAGCATACCCTTCAACTT	CAACTACAACCAAACCTCATTTCA	NA
SNP0095	Ca_22438	*Ca_Kabuli_Chr04*	[C/A]	35574379	CDS_NON_SYNONYMOUS	Transcription factor, MADS-box	GCATGTTCAATTATCCAATGTCTC	GGCAAGAAAGTATTCGTGTTGAAT	NA
SNP0096	Ca_14853	*Ca_Kabuli_Chr04*	[A/G]	36153818	CDS_NON_SYNONYMOUS	Protein of unknown function DUF827, plant	GAAACAAGATTTATGGGGTGTCTC	CCTTCTCTATTTCCACCTTCAGAG	NA
SNP0097	Ca_14825	*Ca_Kabuli_Chr04*	[C/T]	36537170	CDS_NON_SYNONYMOUS	Transcriptional factor B3	AACCATCTAATCCTTTCTGTCGAG	ATTTCCGACACTAACACAGATTCA	NA
SNP0098	Ca_15144	*Ca_Kabuli_Chr04*	[C/A]	37316700	CDS_NON_SYNONYMOUS	Zinc finger, RING-type	CCCTCAAATAATGATGTGACAA	TCGACGATGTAAAGAGATTTTG	NA
SNP0099	Ca_13141	*Ca_Kabuli_Chr04*	[C/T]	38038865	CDS_NON_SYNONYMOUS	Protein of unknown function DUF3326	CAGATTACAACCACTACGCTTGAG	TTCGTACAGTGTCAACCAAAACTT	NA
SNP0100	Ca_13134	*Ca_Kabuli_Chr04*	[T/A]	38195411	CDS_NON_SYNONYMOUS	Domain of unknown function DUF250	ATTACCATATGCACGCAAGTACAG	CACGTGTATTTTGGTTTCTTTCTG	NA
SNP0101	Ca_13128	*Ca_Kabuli_Chr04*	[C/G]	38288877	CDS_NON_SYNONYMOUS	Protein of unknown function DUF869, plant	CCCCCTAAGTTTCTCCTCATAAAT	CCTCACTCAACTGAAGTTGTGACT	NA
SNP0102	Ca_13115	*Ca_Kabuli_Chr04*	[T/A]	38425720	CDS_NON_SYNONYMOUS	Zinc finger, CCCH-type	ACTACCTGACTGATTTTTGCTTCC	CTAGGATTCAGGATGGTCAAGATT	NA
SNP0103	Ca_13102	*Ca_Kabuli_Chr04*	[T/A]	38659534	CDS_NON_SYNONYMOUS	Transcription factor GRAS	ATGCCAAGCTATACTCGTTAAAGG	AACATTCACCCTATCCTGACTCTC	NA
SNP0104	Ca_14868	*Ca_Kabuli_Chr04*	[G/T]	40583205	CDS_NON_SYNONYMOUS	Zinc finger, RING-type	TCACAGTGACAACTGCAATACATC	TTGATGACCAAATTGTTAGGAGTC	NA
SNP0105	Ca_10988	*Ca_Kabuli_Chr04*	[T/C]	41228918	CDS_NON_SYNONYMOUS	Zinc finger, RING-type	AAGAATATGACACCCCTCATTCTC	AACTCCAAAAGTGGAGACAAATTC	NA
SNP0106	Ca_10932	*Ca_Kabuli_Chr04*	[C/G]	41864846	CDS_NON_SYNONYMOUS	WD40 repeat	AACTCAACCTTACAAAACCAGCTT	ATATTTATTGCCAGGACAAACGAG	NA
SNP0107	Ca_09097	*Ca_Kabuli_Chr04*	[A/T]	44239883	CDS_NON_SYNONYMOUS	BTB/POZ-like	TCTATTTTAGGCAATAGGGCGATA	TATGAGATTAGCCGTGATTCTTTG	NA

SNP IDs	*Kabuli* /*desi* gene accession IDs	*Kabuli* /*desi* chromosomes	SNPs	Physical positions (bp)	Sequence components of genome	Putative functions	Forward primer sequences (5'-3')	Reverse primer sequences (5'-3')	Primers used in Figure 2A
SNP0108	Ca_09101	*Ca_Kabuli_Chr04*	[G/A]	44266819	CDS_NON_SYNONYMOUS	DNA-binding WRKY	GTTGGTAGCATTGACATTGATGAT	GATTTCAAGAGAGATTGCAAGGTT	NA
SNP0109	Ca_09120	*Ca_Kabuli_Chr04*	[T/A]	44472237	CDS_NON_SYNONYMOUS	Protein of unknown function DUF1350	CTATGTTTGATGGTTTGAGACACC	AATATAGGTGTCAGTGTCGTGTCG	NA
SNP0110	Ca_09185	*Ca_Kabuli_Chr04*	[G/C]	45224997	CDS_NON_SYNONYMOUS	Protein of unknown function DUF647	CTAGTGCTGCAATGTCACTGCTAT	GTCATTAATTCACCTCATTGCTTG	NA
SNP0111	Ca_19256	*Ca_Kabuli_Chr04*	[C/T]	46612244	CDS_NON_SYNONYMOUS	Protein of unknown function DUF827, plant	CTTCTTTTCTCACCAAGAGTGATG	ACATATGGAAGCAGAAGAACACAG	NA
SNP0112	Ca_10746	*Ca_Kabuli_Chr04*	[T/C]	48464493	CDS_NON_SYNONYMOUS	Zinc finger, CCHC-type	CGCCGTTAAGTCTAACCATTATTT	TTCAGTTCCAGGTGTGATTACATT	NA
SNP0113	Ca_18171	*Ca_Kabuli_Chr05*	[G/C]	166217	CDS_NON_SYNONYMOUS	No apical meristem (NAM) protein	AGGAGCTGATAGGATGATCAGAAC	TGAGGAATAATGAGAGTGACAAGC	NA
SNP0114	Ca_18178	*Ca_Kabuli_Chr05*	[A/C]	234410	CDS_NON_SYNONYMOUS	Protein of unknown function DUF647	TCATTTTCCAGGTAGTCCTTAACC	TCTGACATCTTCTGGTTACATGGT	NA
SNP0115	Ca_26279	*Ca_Kabuli_Chr05*	[T/G]	1280977	CDS_NON_SYNONYMOUS	Transcription factor GRAS	AAATCTGATTCCGAAGAACAAGTC	CTATGGAAATGGACATGGATACAA	NA
SNP0116	Ca_22620	*Ca_Kabuli_Chr05*	[T/G]	5205261	CDS_NON_SYNONYMOUS	Domain of unknown function DUF641	TATATTTGAGGTCGTGGATCATGT	CATCCATTATCAGATTCTTGACCA	NA
SNP0117	Ca_20508	*Ca_Kabuli_Chr05*	[C/T]	9961380	CDS_NON_SYNONYMOUS	DNA-binding WRKY	TACCAAACCGACAGTTGATTATTG	ACGACTCCTAACATGTCAATTGTG	NA
SNP0118	Ca_17110	*Ca_Kabuli_Chr05*	[T/C]	11864959	CDS_NON_SYNONYMOUS	Zinc finger, CCHC-type	CATTTGTAACAGTTTCCACCACTG	AATCTTTTGCTGCTGGTAAGAGTT	NA
SNP0119	Ca_17657	*Ca_Kabuli_Chr05*	[A/C]	24090515	CDS_NON_SYNONYMOUS	SANT domain, DNA binding	GGAGAAAGAAAAAGAAGTTGACCA	TCTTTTGTAGCCTCAGAATCATCC	NA
SNP0120	Ca_17656	*Ca_Kabuli_Chr05*	[T/C]	24097823	CDS_NON_SYNONYMOUS	Protein of unknown function DUF3437	CAGTGGTAGCTACTGTTGGAGGTA	TTCTTCTTTGAAGTTCTCATCACG	NA
SNP0121	Ca_17653	*Ca_Kabuli_Chr05*	[G/C]	24153061	CDS_NON_SYNONYMOUS	Protein of unknown function DUF3437	ACTCCTAATGGTATAGAGGCGTCA	TTCAGAAGTGACAACATCATGAGA	NA
SNP0122	Ca_09042	*Ca_Kabuli_Chr05*	[A/G]	25400604	CDS_NON_SYNONYMOUS	Zinc finger, CCCH-type	AGCATCTTCTCCTATGAATGGAAC	ACGACAATGACATGTAGACACTGA	NA
SNP0123	Ca_08907	*Ca_Kabuli_Chr05*	[C/G]	27362517	CDS_NON_SYNONYMOUS	BTB/POZ-like	TAACAAACTCGTTAAACGTTGTCG	ATGCACTCTGTCTCATTTTCAAAC	NA
SNP0124	Ca_13370	*Ca_Kabuli_Chr05*	[G/A]	28562734	CDS_NON_SYNONYMOUS	Domain of unknown function DUF399	GTCTACATCTGCATTTTGTCTGCT	ACTAATTTTGACATGACCCCACTT	NA
SNP0125	Ca_13361	*Ca_Kabuli_Chr05*	[G/A]	28682995	CDS_NON_SYNONYMOUS	BTB/POZ-like	AGGATTCAACATAGAGGAGTTTCT	TACCTGCACATAAATCTGTTTTGG	NA
SNP0126	Ca_13354	*Ca_Kabuli_Chr05*	[A/T]	28757467	CDS_NON_SYNONYMOUS	Protein of unknown function DUF827, plant	CATATCCTTCAGGAACTTGCTTTT	TTTAAGACCGGGGAGTAAGTATTG	NA
SNP0127	Ca_04684	*Ca_Kabuli_Chr05*	[A/C]	30086232	CDS_NON_SYNONYMOUS	Protein of unknown function DUF300	CTTTGTTTTTGAAGGGAGGTTTAG	TCCAGGGTTTAAGGAAATAGTTGA	NA
SNP0128	Ca_04722	*Ca_Kabuli_Chr05*	[G/A]	30438913	CDS_NON_SYNONYMOUS	SANT domain, DNA binding	CTGAACACTTGCTTGAAATTCTTG	TGATGTTCCTCCCTAATAAGCAAT	NA
SNP0129	Ca_04825	*Ca_Kabuli_Chr05*	[T/C]	31194974	CDS_NON_SYNONYMOUS	High mobility group, HMG1/HMG2	ACCAAATCATGACAAGAGCTTACA	GAACTGCTAAATTAGGGTTTCGAG	NA
SNP0130	Ca_04880	*Ca_Kabuli_Chr05*	[T/C]	31758166	CDS_NON_SYNONYMOUS	SANT domain, DNA binding	CTTTTACAGCTGAGGGGTTTATGT	GGAGATTGTGCATTTAGTTGTGAC	NA
SNP0131	Ca_04898	*Ca_Kabuli_Chr05*	[T/C]	31935674	CDS_NON_SYNONYMOUS	Protein of unknown function DUF869, plant	ACAGTCGAATCTATGTTGGAATCA	TTTGGAGGATCAAGTGAAGACATA	NA
SNP0132	Ca_01919	*Ca_Kabuli_Chr05*	[C/T]	33379873	CDS_NON_SYNONYMOUS	Homeobox	TGCATAAGCAGGAGAACATAATTC	GGAGGTCAGGTTATTCTTCCACTA	NA
SNP0133	Ca_01800	*Ca_Kabuli_Chr05*	[A/C]	34441581	CDS_NON_SYNONYMOUS	Transcription elongation factor S-II, central domain	AAACTGTCACTGAAAGGAACATCA	TTCTTCTTCCAACCAATCTTCTCT	NA
SNP0134	Ca_01790	*Ca_Kabuli_Chr05*	[G/T]	34511669	CDS_NON_SYNONYMOUS	WD40 repeat	CAAGTAACAAAGCCACGGACTAAT	GTATGGTTGATGACAACATGGAAT	NA

SNP IDs	*Kabuli* /*desi* gene accession IDs	*Kabuli* /*desi* chromosomes	SNPs	Physical positions (bp)	Sequence components of genome	Putative functions	Forward primer sequences (5'-3')	Reverse primer sequences (5'-3')	Primers used in Figure 2A
SNP0135	Ca_01550	*Ca_Kabuli_Chr05*	[A/T]	36735459	CDS_NON_SYNONYMOUS	Helix-loop-helix DNA-binding domain	CACTATTCTTTCTTTCTCGCCAAT	ACTTGGCTAAGTTTTCCTTACCG	NA
SNP0136	Ca_01473	*Ca_Kabuli_Chr05*	[A/G]	37460980	CDS_NON_SYNONYMOUS	BEACH domain	CTCTCCCTGCTATCTTGTGTTTTT	ATTTTGAACTGCCTTGTTTAGAGC	NA
SNP0137	Ca_01417	*Ca_Kabuli_Chr05*	[C/T]	38057582	CDS_NON_SYNONYMOUS	Zinc finger, DHHC-type, palmitoyltransferase	ATGCTAACGATAGTTCCTTTTGGT	TTTCACGAGTATCCTCTCAAACAA	NA
SNP0138	Ca_07428	*Ca_Kabuli_Chr05*	[C/A]	38783604	CDS_NON_SYNONYMOUS	Mediator complex subunit Med28	CTTCTCATCTGGACAGAGTTTCAA	CCAAGGGCAGCTTTACATTATTAC	NA
SNP0139	Ca_07534	*Ca_Kabuli_Chr05*	[A/G]	40078077	CDS_NON_SYNONYMOUS	Ethylene insensitive 3	GGAAACCTGAGACTCTTCTTCATC	GTATATTTCCAGTTCCACCAGCTT	NA
SNP0140	Ca_07550	*Ca_Kabuli_Chr05*	[G/C]	40239397	CDS_NON_SYNONYMOUS	Protein of unknown function DUF247	CCAAGTTCTAGTGCCAAACTTTTT	TGGTGACAAGAGGTTAGTCTCAAT	NA
SNP0141	Ca_07642	*Ca_Kabuli_Chr05*	[T/C]	41283253	CDS_NON_SYNONYMOUS	Protein of unknown function DUF1296	AGGACAGTACCTTCACATCCTCAT	AGTTTGTGTAACGGTTGCTTAGGT	NA
SNP0142	Ca_19218	*Ca_Kabuli_Chr05*	[C/T]	41635262	CDS_NON_SYNONYMOUS	Zinc finger, RING-type	ACAGACAGAACACTGCAAATTCTC	GTGTCAAATACGTTCATGTTGCTT	NA
SNP0143	Ca_11376	*Ca_Kabuli_Chr05*	[C/A]	42422932	CDS_NON_SYNONYMOUS	Zinc finger, C2H2-type	AGAAACTTGAGGACCAAAGATGTC	CACCCTATAAGCAAGTCCTGTTCT	NA
SNP0144	Ca_11356	*Ca_Kabuli_Chr05*	[T/A]	42582262	CDS_NON_SYNONYMOUS	Transcription factor, SBP-box	TGCAGATTACTGTGATCATTTGTG	CTGGAATCATCAGAAAGCATAGTG	NA
SNP0145	Ca_12641	*Ca_Kabuli_Chr05*	[C/G]	44159237	CDS_NON_SYNONYMOUS	SANT domain, DNA binding	CTTTTGATGCGATTCTTACACATC	TAAAACTGCACCATTCTAGTTCCA	NA
SNP0146	Ca_03889	*Ca_Kabuli_Chr05*	[G/A]	44428965	CDS_NON_SYNONYMOUS	SET domain	GAACAAATGCTGTGATGATTTCTC	CCTGTGAACAGTAATGATTTTTGC	NA
SNP0147	Ca_03920	*Ca_Kabuli_Chr05*	[G/T]	44760347	CDS_NON_SYNONYMOUS	Transcription factor GRAS	AGTTCAGGAGCTTCACTGCTTACT	AGACTATCTTGTTCACCTCCAAGC	NA
SNP0148	Ca_03964	*Ca_Kabuli_Chr05*	[G/T]	45219258	CDS_NON_SYNONYMOUS	Protein of unknown function DUF455	GTACTGCGTATCAATGTTGTCCAT	GGCCTCAAATTTGTAATACTCGAA	NA
SNP0149	Ca_03969	*Ca_Kabuli_Chr05*	[T/G]	45254112	CDS_NON_SYNONYMOUS	Zinc finger, C2H2-type matrin	TTAAGGCTCGTTTGATTCTCATTC	ACAACAAGCACTGGTGTTTTCAT	NA
SNP0150	Ca_04004	*Ca_Kabuli_Chr05*	[C/A]	45559874	CDS_NON_SYNONYMOUS	WD40 repeat	ACACATGACTTTGCACCTCTTTTA	GCAGTTAGATTCCCAACTATGCTT	NA
SNP0151	Ca_04069	*Ca_Kabuli_Chr05*	[T/C]	46176366	CDS_NON_SYNONYMOUS	No apical meristem (NAM) protein	TCTTTCTATCATCATCACCACTGC	ATGGGTTAGGAAGTCGAATGAGTA	NA
SNP0152	Ca_04211	*Ca_Kabuli_Chr05*	[G/A]	47518533	CDS_NON_SYNONYMOUS	Zinc finger, RING-type	TATGGACAGATTGTTCCACTCTTG	AGGGAGAGGTCTTTTATGTTGATG	NA
SNP0153	Ca_04233	*Ca_Kabuli_Chr05*	[A/G]	47733645	CDS_NON_SYNONYMOUS	No apical meristem (NAM) protein	GAATGGTACTTCTTCTCTCCAAGG	TACATTGGCAGCTGTTATTAATGC	NA
SNP0154	Ca_04239	*Ca_Kabuli_Chr05*	[A/G]	47774516	CDS_NON_SYNONYMOUS	Protein of unknown function DUF869, plant	CAGTCCTAGAGATTCGATGGTTTT	TCTTGAAGACCAAGTTTGTCATGT	NA
SNP0155	Ca_13257	*Ca_Kabuli_Chr06*	[C/T]	324301	CDS_NON_SYNONYMOUS	Zinc finger, FYVE-type	TCTGGTGTTTGTGAATCACTGTAA	CCGCACGATATCATTTTAACATAC	NA
SNP0156	Ca_10329	*Ca_Kabuli_Chr06*	[T/G]	2079137	CDS_NON_SYNONYMOUS	Protein of unknown function DUF760	AAGCCAGGTAGATACTTCAGGATG	AGTAGAGAAGATACGTGGCAATCC	NA
SNP0157	Ca_10431	*Ca_Kabuli_Chr06*	[A/G]	2925908	CDS_NON_SYNONYMOUS	Tify	TTCTCTCTTCTGTTCACGTTTTTG	GTAAGTGAAAAATGAAAGCCACCT	NA
SNP0158	Ca_05766	*Ca_Kabuli_Chr06*	[C/A]	5341510	CDS_NON_SYNONYMOUS	ARID/BRIGHT DNA-binding domain	GCTATTTGTCCAAAAAGGACCTAA	CCTGATAAAGGAGAAAGCAGAAAG	NA
SNP0159	Ca_16913	*Ca_Kabuli_Chr06*	[C/G]	6443828	CDS_NON_SYNONYMOUS	SANT domain, DNA binding	ACATGGCCACTAGTCAATAAACAA	GCTTGGTCTAAAGAAGAAGACGAC	NA
SNP0160	Ca_16923	*Ca_Kabuli_Chr06*	[C/T]	6631216	CDS_NON_SYNONYMOUS	Protein of unknown function DUF668	TCCTAATCCTGATTTTGAGCTAGG	AAAGTCGAAGGCAGATTTTATACG	NA
SNP0161	Ca_16938	*Ca_Kabuli_Chr06*	[G/C]	6779147	CDS_NON_SYNONYMOUS	Protein of unknown function DUF869, plant	GGTGACAATATGAGTGGTGACTTT	TCATCTCCCATCCACCTAATTTAT	NA

SNP IDs	*Kabuli* /*desi* gene accession IDs	*Kabuli* /*desi* chromosomes	SNPs	Physical positions (bp)	Sequence components of genome	Putative functions	Forward primer sequences (5'-3')	Reverse primer sequences (5'-3')	Primers used in Figure 2A
SNP0162	Ca_16954	*Ca_Kabuli_Chr06*	[A/C]	6913622	CDS_NON_SYNONYMOUS	SNF2-related	TAAGTGTCAGTCCTAAACCACCAA	ACTCATCAAGTGTATGGAATTGGA	NA
SNP0163	Ca_09624	*Ca_Kabuli_Chr06*	[G/A]	7603195	CDS_NON_SYNONYMOUS	Domain of unknown function DUF699, exodeoxyribonuclease V alpha chain	CCATATCTGGTCTTCCTGTCTTCT	GTAGCACAAATAAGTGGTGAGCTG	NA
SNP0164	Ca_08667	*Ca_Kabuli_Chr06*	[A/C]	9324122	CDS_NON_SYNONYMOUS	Homeobox	TAGATCCATTGTGCCAAAGTAAGA	GATGCTATCAATGGATTTGTTGAC	NA
SNP0165	Ca_08608	*Ca_Kabuli_Chr06*	[C/T]	9834183	CDS_NON_SYNONYMOUS	WD40 repeat	TATGGTGAAACAGAGTTTGAAGGA	CCAGGTCTGTGCATAAAACTACAA	NA
SNP0166	Ca_08544	*Ca_Kabuli_Chr06*	[T/C]	10510996	CDS_NON_SYNONYMOUS	Protein of unknown function DUF803	ACAGCTTTCACACCCATAACCTAT	GAATAAAAGCACCTCGCAAAGTAG	NA
SNP0167	Ca_20467	*Ca_Kabuli_Chr06*	[C/A]	10991652	CDS_NON_SYNONYMOUS	Zinc finger, MYND-type	CCCTTTTGCCTTAGTTTTTGTATC	ACCGTTAACCATAGCCATAATACC	NA
SNP0168	Ca_23423	*Ca_Kabuli_Chr06*	[A/G]	11394963	CDS_NON_SYNONYMOUS	Zinc finger, MYND-type	TACCTTGGTAAATGATCACCTTCA	TTTAAAAGTGTCACCACCATGACT	NA
SNP0169	Ca_05025	*Ca_Kabuli_Chr06*	[A/T]	12437275	CDS_NON_SYNONYMOUS	AUX/IAA protein	ACTCACAACATTTCCAATGTATCC	TACAATGTAGCAAGGGAGTGAAAG	NA
SNP0170	Ca_05052	*Ca_Kabuli_Chr06*	[T/G]	12799890	CDS_NON_SYNONYMOUS	Protein of unknown function DUF2359, TMEM214	TCCCTTTTCATCATCTTCTTCTTC	CATTGAATCTCCAACTAGCCAATA	NA
SNP0171	Ca_05104	*Ca_Kabuli_Chr06*	[C/G]	13326969	CDS_NON_SYNONYMOUS	Zinc finger, RING-type	AGTTTGAAATCGCTTCTTCATCTC	TAGGAGCCACTGAATTTTGCTTAT	NA
SNP0172	Ca_05200	*Ca_Kabuli_Chr06*	[C/T]	14309042	CDS_NON_SYNONYMOUS	Domain of unknown function DUF640	AGTGCGAGTATTCCAACTTAACAA	ACAAACCCACCTCAAAAACTCTAC	NA
SNP0173	Ca_05213	*Ca_Kabuli_Chr06*	[A/G]	14416997	CDS_NON_SYNONYMOUS	Domain of unknown function DUF676, hydrolase-like	TCATATTGTCCTGTCCACTTTGAT	GTGCTATACCTCGCCTATGAAGTT	NA
SNP0174	Ca_06319	*Ca_Kabuli_Chr06*	[C/G]	16932014	CDS_NON_SYNONYMOUS	WD40 repeat	CTCATTACTGTTTCATGGTTTGGA	TACCCATTCATTATGTCCAACAAG	NA
SNP0175	Ca_06326	*Ca_Kabuli_Chr06*	[A/C]	17048493	CDS_NON_SYNONYMOUS	Zinc finger, Dof-type	TCTCCCCTCCTCCTATACCATATT	TAGTGCTAATTCAAACCCTCCTTC	NA
SNP0176	Ca_06343	*Ca_Kabuli_Chr06*	[C/A]	17258827	CDS_NON_SYNONYMOUS	Homeobox	AGGTAATAAAACCTTTGGTCGTTG	GTTGAGGAACTTCTACAGGTAGCC	NA
SNP0177	Ca_06458	*Ca_Kabuli_Chr06*	[T/C]	18444725	CDS_NON_SYNONYMOUS	Basic-leucine zipper (bZIP) transcription factor	GTGGTAATTTGCAAAGACAAGGTT	ATTGTGGAAATGGGTGCTTATACT	NA
SNP0178	Ca_06467	*Ca_Kabuli_Chr06*	[C/A]	18562366	CDS_NON_SYNONYMOUS	Zinc finger, RING-type	ATAGCCTTTAAGCAACAGTCCAAC	ACTGAATCTGATCAACCAAGTCAA	NA
SNP0179	Ca_16355	*Ca_Kabuli_Chr06*	[C/A]	20766377	CDS_NON_SYNONYMOUS	Helix-loop-helix DNA-binding domain	CATAGTTGAGTCAAATGGAAATGC	ACTCGGTCCTTACAAAGCATAACT	NA
SNP0180	Ca_11272	*Ca_Kabuli_Chr06*	[T/G]	21675734	CDS_NON_SYNONYMOUS	Homeobox	GCTTCCTTTGTTCGTAGTTTCTCT	TACACACACACACACAGGGTCTAC	NA
SNP0181	Ca_11268	*Ca_Kabuli_Chr06*	[G/T]	21718674	CDS_NON_SYNONYMOUS	Myb, DNA-binding	ATTAGCTCGACAAAGTTCCTCAGT	GAGACATGAAAAAGTTCTCCACAA	NA
SNP0182	Ca_11216	*Ca_Kabuli_Chr06*	[T/G]	22217157	CDS_NON_SYNONYMOUS	Zinc finger, CHCC-type	GATTGGAGAAAGTTCCCTCAGTTA	GCCGTGTGAGGTTTTCTAAGTTAC	NA
SNP0183	Ca_11135	*Ca_Kabuli_Chr06*	[A/C]	23166355	CDS_NON_SYNONYMOUS	Zinc finger, PHD-type	AACATCGAACCGAAAGCTATTAAC	AACCCTCTTATCACATCCATCATT	NA
SNP0184	Ca_16676	*Ca_Kabuli_Chr06*	[C/A]	26349363	CDS_NON_SYNONYMOUS	Transcription factor GRAS	GAACTTAACAATTCTGTGCGTTTG	ACTTCTAGGACTGTTCGCTTCAAT	NA
SNP0185	Ca_15224	*Ca_Kabuli_Chr06*	[T/C]	32328468	CDS_NON_SYNONYMOUS	DNA-binding WRKY	ATTAATTTGATACCGTGAGGTTGC	AAATATGCGTACTTTCCTGAATCC	NA
SNP0186	Ca_23252	*Ca_Kabuli_Chr06*	[T/G]	42855091	CDS_NON_SYNONYMOUS	BTB/POZ-like	GATCTAGGCCATGATTGGATAAAC	GACTATCGGATTCGAAAAGAGAAC	NA
SNP0187	Ca_17522	*Ca_Kabuli_Chr06*	[T/C]	52227277	CDS_NON_SYNONYMOUS	Zinc finger, GATA-type	TAGTGAGATATGATTGGGCATTTG	CACCTTGAGTCTGCAGTTTTAGTG	NA
SNP0188	Ca_16263	*Ca_Kabuli_Chr06*	[A/C]	54091997	CDS_NON_SYNONYMOUS	Protein of unknown function DUF3527	AGAACCTACCAACAGGAGAAAGTG	CATCAGCGTTGGCTATACAAGTAG	NA

SNP IDs	*Kabuli* /*desi* gene accession IDs	*Kabuli* /*desi* chromosomes	SNPs	Physical positions (bp)	Sequence components of genome	Putative functions	Forward primer sequences (5'-3')	Reverse primer sequences (5'-3')	Primers used in Figure 2A
SNP0189	Ca_19023	*Ca_Kabuli_Chr06*	[T/A]	55922843	CDS_NON_SYNONYMOUS	Protein of unknown function wound-induced	TCAAAATAGTCTTCCTTCCAATCC	CAACTGAATTCTTAACTCTCCAAGC	NA
SNP0190	Ca_19026	*Ca_Kabuli_Chr06*	[T/A]	55953320	CDS_NON_SYNONYMOUS	Protein of unknown function wound-induced	ATGAATGGTTTTCTGACCCTAATC	TAGAGCATTTGAAGCTCTTCCTTT	NA
SNP0191	Ca_13705	*Ca_Kabuli_Chr06*	[G/A]	57382220	CDS_NON_SYNONYMOUS	Zinc finger, RING-type	AGATTGAGAAGCTCAGTGATACCC	GCAACAATACAAAGTTCACAAAGG	NA
SNP0192	Ca_13700	*Ca_Kabuli_Chr06*	[T/G]	57436320	CDS_NON_SYNONYMOUS	Bromodomain	TGACAATCCTAGTGCCATTGTAGT	GGTTTACTAATCTTGCAGGCTCAT	NA
SNP0193	Ca_13682	*Ca_Kabuli_Chr06*	[G/T]	57634387	CDS_NON_SYNONYMOUS	Protein of unknown function DUF936, plant	CAACTCATTAACCATCCATTGAAC	TTTTTCATTGGTCCACTAGGTCTT	NA
SNP0194	Ca_15410	*Ca_Kabuli_Chr06*	[A/T]	58650459	CDS_NON_SYNONYMOUS	WD40 repeat	AATTCCTTTACATCATCCACTTCC	ATATCAAATACTGCCTCCCAAATG	NA
SNP0195	Ca_15412	*Ca_Kabuli_Chr06*	[A/G]	58672317	CDS_NON_SYNONYMOUS	Zinc finger, B-box	AAGCGAGCTTTACGTTAGAACATT	CAAGCTTTACACCTGTGAGAAGAA	NA
SNP0196	Ca_20223	*Ca_Kabuli_Chr07*	[C/G]	218341	CDS_NON_SYNONYMOUS	Transcription factor GRAS	GAGGGACCAGAAAGTTTGATCTTA	CGTTCTCCTACAATCTGAAGTCCT	NA
SNP0197	Ca_24417	*Ca_Kabuli_Chr07*	[G/T]	610723	CDS_NON_SYNONYMOUS	Transcription factor GRAS	GGTTATACACACATCCCATCTTCC	TAAACTCAATTATGCAGCTTCACC	NA
SNP0198	Ca_03291	*Ca_Kabuli_Chr07*	[T/A]	1787308	CDS_NON_SYNONYMOUS	Domain of unknown function DUF828	AAATAGAGTGCCAGAATGGATGAT	CACTTAGCCAACACATTTTCACTT	NA
SNP0199	Ca_03202	*Ca_Kabuli_Chr07*	[T/G]	2757253	CDS_NON_SYNONYMOUS	Transcription factor jumonji/aspartyl beta-hydroxylase	ACAACTTTGCAAGAGTAACATTGG	CACCTACTGTGAAAACTTTGATGC	NA
SNP0200	Ca_03139	*Ca_Kabuli_Chr07*	[G/T]	3297677	CDS_NON_SYNONYMOUS	Domain of unknown function DUF231, plant	GAATGATGTTGAAGATGTGGGTAA	TGCTTAGAGCATTACAGAATTTGC	NA
SNP0201	Ca_03128	*Ca_Kabuli_Chr07*	[A/G]	3355627	CDS_NON_SYNONYMOUS	AUX/IAA protein	AGTCATAACCCCTTGAAAGCATAG	CATCCATGCTCTCATCAAATACTC	NA
SNP0202	Ca_03113	*Ca_Kabuli_Chr07*	[G/A]	3560182	CDS_NON_SYNONYMOUS	Zinc finger, FYVE-type	CTACAGAAAGAAGTGGACGAGGTT	TTACCATGATCTGTGTCTTTACCG	NA
SNP0203	Ca_03002	*Ca_Kabuli_Chr07*	[C/T]	4497784	CDS_NON_SYNONYMOUS	Domain of unknown function DUF625	CTGGAAGAGGTAAAATTTGGAGAG	GTGGATCTTAAATTCCCCATCTAA	NA
SNP0204	Ca_06816	*Ca_Kabuli_Chr07*	[C/A]	5500318	CDS_NON_SYNONYMOUS	SNF2-related	CTTCGAATGGTACAGGTAGGCTAT	AGTTTATCTCTGACTCCCAACCAG	NA
SNP0205	Ca_06691	*Ca_Kabuli_Chr07*	[G/T]	6703849	CDS_NON_SYNONYMOUS	WD40 repeat	TCATAGATTGTTTTCGTGTGGAAC	GGTCTCAGTTAGGCCGATTATAGA	NA
SNP0206	Ca_06681	*Ca_Kabuli_Chr07*	[G/T]	6814954	CDS_NON_SYNONYMOUS	YABBY protein	CTGGATCCTAGGGTTTTTCTTTTT	CTCACAGTCCCCAATTATCCTTTA	NA
SNP0207	Ca_13243	*Ca_Kabuli_Chr07*	[C/T]	7756455	CDS_NON_SYNONYMOUS	SNF2-related	TGAAAACTACTGCACGTTTAGCTT	TAAGTGTCAGTCCTAAACCACCAA	NA
SNP0208	Ca_15325	*Ca_Kabuli_Chr07*	[A/T]	8894362	CDS_NON_SYNONYMOUS	Domain of unknown function DUF629	GCAACCACTTTCTTCTTCTCTAGG	GGCTTCCAAGAACAATTAAGAATC	NA
SNP0209	Ca_12810	*Ca_Kabuli_Chr07*	[C/T]	10485414	CDS_NON_SYNONYMOUS	Protein of unknown function DUF702	AAAGGTCGTACAAATGTTGTTCCT	AGCATTTTCCTTGAACAATACAGC	NA
SNP0210	Ca_12811	*Ca_Kabuli_Chr07*	[C/A]	10521449	CDS_NON_SYNONYMOUS	Zinc finger, RING-type	GGATAGAGAAGGAACAGAAACAGG	TTCTATATTTAACCCATCCGCTTC	NA
SNP0211	Ca_09342	*Ca_Kabuli_Chr07*	[A/G]	11545846	CDS_NON_SYNONYMOUS	Lateral organ boundaries, LOB	TTTACAATAGGGAAGGACCCATTA	TTTCGACGGTTACAAGTTTACACA	NA
SNP0212	Ca_09335	*Ca_Kabuli_Chr07*	[C/A]	11638885	CDS_NON_SYNONYMOUS	BTB/POZ-like	GGCCTTGTTTGGATAAATAGCTTA	AATTATTCCCAGGAGATTCCAAGT	NA
SNP0213	Ca_09247	*Ca_Kabuli_Chr07*	[C/A]	12730600	CDS_NON_SYNONYMOUS	Lateral organ boundaries, LOB	TTGCCAGGTTGTTACAAGATAAAC	TAGTTGTGTTTCCGTTGTTGATG	NA
SNP0214	Ca_09222	*Ca_Kabuli_Chr07*	[C/A]	13059710	CDS_NON_SYNONYMOUS	BTB/POZ-like	GACAGAACAGAAAGAGATTCTGGA	AATTTCATGTCTTGTGCTTCTCAG	NA
SNP0215	Ca_16064	*Ca_Kabuli_Chr07*	[T/G]	13903980	CDS_NON_SYNONYMOUS	Tubby, C-terminal	ATCCACTTTCTCAGCTCTCAAGAC	ACATTTTGCAGGTCTAACTTTTGG	NA

SNP IDs	*Kabuli /desi* gene accession IDs	*Kabuli /desi* chromosomes	SNPs	Physical positions (bp)	Sequence components of genome	Putative functions	Forward primer sequences (5'-3')	Reverse primer sequences (5'-3')	Primers used in Figure 2A
SNP0216	Ca_23041	*Ca_Kabuli_Chr07*	[T/G]	14233343	CDS_NON_SYNONYMOUS	Protein of unknown function DUF248, methyltransferase putative	GATAATCACCCCTTCAACATCAAT	TAATATCGAAGTTTGCAACGTCAC	NA
SNP0217	Ca_23042	*Ca_Kabuli_Chr07*	[G/T]	14281993	CDS_NON_SYNONYMOUS	Protein of unknown function DUF702	TGTAGTGGTTTTTGTCATTGTGTG	TTATTTCATCACTCACGGAAACTG	NA
SNP0218	Ca_15859	*Ca_Kabuli_Chr07*	[G/T]	17318174	CDS_NON_SYNONYMOUS	SET domain	AAAAAGAGGATCCTGATTGTCATC	CCACAAATCCCTAACATTTTATCC	NA
SNP0219	Ca_15849	*Ca_Kabuli_Chr07*	[T/C]	17597348	CDS_NON_SYNONYMOUS	Protein of unknown function DUF1639	ACTGAGACAAGTCATAGCACAAGC	TACTGACAGAGACACCAGACACAA	NA
SNP0220	Ca_15839	*Ca_Kabuli_Chr07*	[A/G]	17779414	CDS_NON_SYNONYMOUS	Basic-leucine zipper (bZIP) transcription factor	ATTGACCAAGGACCTTTTACAGAC	GTGCTAGTCAACAAACGGAATAGA	NA
SNP0221	Ca_12308	*Ca_Kabuli_Chr07*	[T/C]	18796748	CDS_NON_SYNONYMOUS	ZF-HD homeobox protein, Cys/His-rich dimerisation domain	TCTGTCATTTGTCGTAGGACTCAC	CAAAGTAGGCATGAACTCAAATGT	NA
SNP0222	Ca_14522	*Ca_Kabuli_Chr07*	[T/C]	21744514	CDS_NON_SYNONYMOUS	SANT domain, DNA binding	TAATAACAGAATCTGCGTTCTTCC	TCCAAACTAGTCCAAAACTTCTCC	NA
SNP0223	Ca_19916	*Ca_Kabuli_Chr07*	[T/A]	24021690	CDS_NON_SYNONYMOUS	ZF-HD homeobox protein, Cys/His-rich dimerisation domain	CAACTGATCTGCCTCAATCCTAT	AATGATTAGTGGTGGAGATTTTGG	NA
SNP0224	Ca_19912	*Ca_Kabuli_Chr07*	[G/A]	24181321	CDS_NON_SYNONYMOUS	Zinc finger, RING-CH-type	TGAGCTTTAGGGATGCTTTAACTT	GACACATAGGCACAGCACTACAAC	NA
SNP0225	Ca_11718	*Ca_Kabuli_Chr07*	[C/T]	30085847	CDS_NON_SYNONYMOUS	Domain of unknown function DUF828	AGTAAACGCCAGTTACGTGGTAGT	GACCGAATTTAGTAACCGATTTTG	NA
SNP0226	Ca_10028	*Ca_Kabuli_Chr07*	[A/G]	32522203	CDS_NON_SYNONYMOUS	SANT domain, DNA binding	CAGCTTTTCCATCTTGGACTTAAT	GTGTCAGTTTGCTCTTATCTTCCA	NA
SNP0227	Ca_10004	*Ca_Kabuli_Chr07*	[A/G]	32942199	CDS_NON_SYNONYMOUS	Transcription factor GRAS	ATGGTCCAGATCACAAGTTCAATA	AGCCTGAACTGAGCACATACAATA	NA
SNP0228	Ca_16177	*Ca_Kabuli_Chr07*	[A/C]	33573655	CDS_NON_SYNONYMOUS	CCAAT-binding factor	CGTAAGTCTCTCTATGCATTCGAC	TAAAACGGACCAAAGTCTTAAACC	NA
SNP0229	Ca_17639	*Ca_Kabuli_Chr07*	[T/G]	35181619	CDS_NON_SYNONYMOUS	Helix-loop-helix DNA-binding domain	AAACTTGCTGTTAGGAAAATGGAG	GGCTCAAAGTAGAAAAGTTGCATT	NA
SNP0230	Ca_17781	*Ca_Kabuli_Chr07*	[T/C]	35799073	CDS_NON_SYNONYMOUS	BTB/POZ-like	CCTGAAAATCAAGATGAAGAGGTT	GAAGAACTGCAACAAAAGAAACAG	NA
SNP0231	Ca_13756	*Ca_Kabuli_Chr07*	[C/A]	36711954	CDS_NON_SYNONYMOUS	Mediator complex, subunit Med12	CAGGTATGAGAACATTCTTGTTGC	TATCGGAGCTATTCAACTGTAGCA	NA
SNP0232	Ca_19581	*Ca_Kabuli_Chr07*	[G/A]	39181880	CDS_NON_SYNONYMOUS	BEACH domain	GCTGGTCTGTTAGGTGTACTGTTG	AACTCAAAAGGACGACTTTCGTAT	NA
SNP0233	Ca_17996	*Ca_Kabuli_Chr07*	[A/C]	41387475	CDS_NON_SYNONYMOUS	WD40 repeat	TCATGATACCACTTTGGCTTTTAG	CATCATACCCTACACCTCCAACTT	NA
SNP0234	Ca_15697	*Ca_Kabuli_Chr07*	[C/A]	44612651	CDS_NON_SYNONYMOUS	Ethylene insensitive 3	AGCAACCTTTATGTTGTTTTCTCC	AGGAGCTTCATCATAAGGAAAATG	NA
SNP0235	Ca_15708	*Ca_Kabuli_Chr07*	[A/C]	44877324	CDS_NON_SYNONYMOUS	Zinc finger, C2H2-type	GGTCTGACCTTCTTTTACATGCTT	AGGCCTGTCCTTCTACATATTGAC	NA
SNP0236	Ca_11909	*Ca_Kabuli_Chr08*	[T/A]	281608	CDS_NON_SYNONYMOUS	Helix-loop-helix DNA-binding domain	GGCTGACTTTATGAACCACTTTCT	GTACGTTACGGAAGGTAAAATTGC	NA
SNP0237	Ca_11856	*Ca_Kabuli_Chr08*	[T/C]	709063	CDS_NON_SYNONYMOUS	Protein of unknown function DUF1950	CACTTTTTCAAGGAGGGATTATGT	ATTATGCATGTTTGTGTTGAGCAG	NA
SNP0238	Ca_11855	*Ca_Kabuli_Chr08*	[A/G]	714967	CDS_NON_SYNONYMOUS	Protein of unknown function DUF1950	GGGACCATCTTATTTTAAACATGC	CCTTAGGCTTAGTGCCTTATTGAA	NA
SNP0239	Ca_11839	*Ca_Kabuli_Chr08*	[A/G]	822302	CDS_NON_SYNONYMOUS	Zinc finger, PHD-type	AGACAAACAGGGTTGTTGAGTACA	ATCTGTGTATCTCCCAAAAAGGAG	NA
SNP0240	Ca_11834	*Ca_Kabuli_Chr08*	[G/A]	855332	CDS_NON_SYNONYMOUS	Protein of unknown function DUF827, plant	TACCAAGAGTTGCAAAGAGTTCAG	CTTCTTCCTCGGTTTCTTTCAATA	NA
SNP0241	Ca_11832	*Ca_Kabuli_Chr08*	[A/C]	866741	CDS_NON_SYNONYMOUS	SANT domain, DNA binding	TAAGAAGGTGAAGTGGGGAGTTAG	TGAGTGTGTTTTTGTCCTTTTAGG	NA
SNP0242	Ca_15078	*Ca_Kabuli_Chr08*	[T/C]	1070728	CDS_NON_SYNONYMOUS	WD40 repeat	CATATTGCTATTGGTTTGAACAGC	TAGTCTTCCAAACCGAACTTTGAT	NA

SNP IDs	*Kabuli* /*desi* gene accession IDs	*Kabuli* /*desi* chromosomes	SNPs	Physical positions (bp)	Sequence components of genome	Putative functions	Forward primer sequences (5'-3')	Reverse primer sequences (5'-3')	Primers used in Figure 2A
SNP0243	Ca_15049	*Ca_Kabuli_Chr08*	[A/G]	1265535	CDS_NON_SYNONYMOUS	Zinc finger, CCCH-type	TTCCTCTAAAACACTCACACTTCG	TCTCGTGAGTTTTGGTAATGAGTC	NA
SNP0244	Ca_15035	*Ca_Kabuli_Chr08*	[G/A]	1382266	CDS_NON_SYNONYMOUS	Zinc finger, PHD-type	TTCTGATGAGGTTGGTAATTGGTA	TCGACAGTTTGAAAGTTAAGACCA	NA
SNP0245	Ca_02440	*Ca_Kabuli_Chr08*	[C/T]	1675888	CDS_NON_SYNONYMOUS	Protein of unknown function DUF604	CTCTGCATGCTATCTATCTCCTCA	ATGAGCATTCACCAATGAGTACAG	NA
SNP0246	Ca_02313	*Ca_Kabuli_Chr08*	[T/C]	2664625	CDS_NON_SYNONYMOUS	Zinc finger, GATA-type	AATATCCAATGAGTGAGGCGTATT	CAAATCCTAATTGGAAGGTCTTGT	NA
SNP0247	Ca_02249	*Ca_Kabuli_Chr08*	[T/C]	3297410	CDS_NON_SYNONYMOUS	SANT domain, DNA binding	AGATAGGTATGCATGCTAGTTCCA	ATGTTTAGAAATTCAAGGCCTCAC	NA
SNP0248	Ca_02240	*Ca_Kabuli_Chr08*	[T/C]	3396820	CDS_NON_SYNONYMOUS	BSD	CAGTGACAACCATACATAGCACAA	ATTTGTCTGATGCACAACAAGAAC	NA
SNP0249	Ca_02167	*Ca_Kabuli_Chr08*	[G/A]	4042424	CDS_NON_SYNONYMOUS	Protein of unknown function DUF869, plant	CCTATGTGTGCTCATGTATTTTCC	TGGAGGCTTAAATGAGAAGGATTA	NA
SNP0250	Ca_02163	*Ca_Kabuli_Chr08*	[C/T]	4073528	CDS_NON_SYNONYMOUS	Zinc finger, RING-type	GAATCAGAGCCAATTTAAGGAAAG	GTAAGAGTGACTTGTCCTCCTGGT	NA
SNP0251	Ca_02124	*Ca_Kabuli_Chr08*	[A/G]	4421609	CDS_NON_SYNONYMOUS	Lateral organ boundaries, LOB	AATCCTCAAAGGTTCACATACGTT	TTTCACAAGATGCTAGGACAGCTA	NA
SNP0252	Ca_02117	*Ca_Kabuli_Chr08*	[T/C]	4483130	CDS_NON_SYNONYMOUS	Zinc finger, LIM-type	GAAGAAGAAGATGGTGCATCAATA	GTTAAGATTCTTGGGGGAGAGTTT	NA
SNP0253	Ca_02096	*Ca_Kabuli_Chr08*	[C/T]	4611094	CDS_NON_SYNONYMOUS	SET domain	TGAAGAAGACATGATAAACGAACG	TGAAGGTATAGAGCCAATTGTCAG	NA
SNP0254	Ca_02034	*Ca_Kabuli_Chr08*	[G/T]	5197004	CDS_NON_SYNONYMOUS	Homeobox	CTGCAATTAATACAACCATCATCC	TAGCTTGTTTGTTATTGTGCATCC	NA
SNP0255	Ca_02005	*Ca_Kabuli_Chr08*	[G/A]	5399503	CDS_NON_SYNONYMOUS	Protein of unknown function DUF248, methyltransferase putative	AAATCTGCATGTAGGTGTTCTTTG	AAGATGTCCAGATATGGAAGGGTA	NA
SNP0256	Ca_01989	*Ca_Kabuli_Chr08*	[C/T]	5539044	CDS_NON_SYNONYMOUS	Protein of unknown function DUF726	TAATATCTCAACCCCTAGCCTCTG	TCTGCTTGTTTATCGGATTTAGGT	NA
SNP0257	Ca_10686	*Ca_Kabuli_Chr08*	[A/C]	7041901	CDS_NON_SYNONYMOUS	Transcription factor jumonji/aspartyl beta-hydroxylase	GCGGTGTATTGAAAATAAAAGGAG	ATTGAGGTATCACAGCATGGTTTA	NA
SNP0258	Ca_11471	*Ca_Kabuli_Chr08*	[C/T]	8389117	CDS_NON_SYNONYMOUS	Transcription factor jumonji/aspartyl beta-hydroxylase	TATGAGTGGAAGGTCAGTCCCTAT	AAACACTTATACATGCCCTCGATT	NA
SNP0259	Ca_16839	*Ca_Kabuli_Chr08*	[C/G]	11186919	CDS_NON_SYNONYMOUS	Zinc finger, RanBP2-type	CCTAGATGCAATACTCGATTGGTC	TTTTCCAGAAGCAGTAACAAACAG	NA
SNP0260	Ca_16820	*Ca_Kabuli_Chr08*	[G/A]	11403754	CDS_NON_SYNONYMOUS	SANT domain, DNA binding	ACGGCGTCTTTTAGTAGTTTGACT	TGTAGGGACAGGTAATGCATTTTA	NA
SNP0261	Ca_15527	*Ca_Kabuli_Chr08*	[C/G]	16145411	CDS_NON_SYNONYMOUS	Domain of unknown function DUF292, eukaryotic	CAGCTATTGCAGCCAGTATCTTTA	GCTCTTGAGGAATAAGAGAGATGC	NA
SNP0262	Ca_25873	*Ca_Kabuli_Scaffold_1313*	[G/A]	18412	CDS_NON_SYNONYMOUS	Homeobox	ATTGGAGCATCTCTTGTATTCCTC	TGAAATAATCACCTGGTTACGAGA	NA
SNP0263	Ca_22581	*Ca_Kabuli_Scaffold_134*	[T/G]	218336	CDS_NON_SYNONYMOUS	SET domain	TTCGTCAAAACAGAGTCTCTTCAT	AAACCAGGATAATCTCCATACTGC	NA
SNP0264	Ca_08743	*Ca_Kabuli_Scaffold_1348_1*	[G/T]	553863	CDS_NON_SYNONYMOUS	Zinc finger, RING-type	GCCTTTAATCGGAAGTTCGTC	ACTTCCTCTTGACCATGGATAGTG	NA
SNP0265	Ca_08787	*Ca_Kabuli_Scaffold_1348_1*	[A/C]	959608	CDS_NON_SYNONYMOUS	Transcription factor jumonji/aspartyl beta-hydroxylase	GAGGATCATGATTTGCATAGTTTG	CTCATAATCGATTCCCATTTCTTC	NA
SNP0266	Ca_21995	*Ca_Kabuli_Scaffold_1351*	[T/C]	172110	CDS_NON_SYNONYMOUS	Protein of unknown function DUF2419	ACTGCACAAGCATAATTCAATCAC	AGCAGCTGATCTATTTGAAGAACA	NA
SNP0267	Ca_26665	*Ca_Kabuli_Scaffold_2557*	[C/T]	110378	CDS_NON_SYNONYMOUS	Domain of unknown function DUF828	ACCTACATCTGGTGAGTCCTAAGC	GGGCTAGTTCTGTTAATCTGCATT	NA
SNP0268	Ca_24396	*Ca_Kabuli_Scaffold_2763*	[G/A]	43207	CDS_NON_SYNONYMOUS	Zinc finger, RING-type	AGGCCGTTATCTGTTTAAATACCA	AGACCAAATTCCGTTCTCTTACAC	NA
SNP0269	Ca_27794	*Ca_Kabuli_Scaffold_2845*	[G/A]	7107	CDS_NON_SYNONYMOUS	Protein of unknown function DUF652	TTCATTTGTCCAGGTTGTAAGCTA	GGAAACATAAAGCAAGAACTGTCA	NA

SNP IDs	*Kabuli* /*desi* gene accession IDs	*Kabuli* /*desi* chromosomes	SNPs	Physical positions (bp)	Sequence components of genome	Putative functions	Forward primer sequences (5'-3')	Reverse primer sequences (5'-3')	Primers used in Figure 2A
SNP0270	Ca_21814	*Ca_Kabuli_Scaffold_314*	[C/G]	610535	CDS_NON_SYNONYMOUS	Zinc finger, CCCH-type	CTCTCTTGCTTCTGAAACAGATCA	AAGCATCACAAATTGCACAGTTAC	NA
SNP0271	Ca_28178	*Ca_Kabuli_Scaffold_3337*	[G/A]	17956	CDS_NON_SYNONYMOUS	Domain of unknown function DUF828	CCTTGGATCACTTGTTCTATTTGA	TCGGGTTTATGTTTTAGGGTTAAG	NA
SNP0272	Ca_26273	*Ca_Kabuli_Scaffold_3397*	[T/C]	45363	CDS_NON_SYNONYMOUS	WD40 repeat	TTGATAGGAAGCAGGATATTGACA	ATGTCTCTGCAATGCATTAGATTC	NA
SNP0273	Ca_22195	*Ca_Kabuli_Scaffold_420*	[T/G]	34145	CDS_NON_SYNONYMOUS	Zinc finger, PHD-type	TGACTATTGTGGTTTTCTCCTTGA	CAAGTCTATTAACAGCCTCCACAA	NA
SNP0274	Ca_22883	*Ca_Kabuli_Scaffold_421_2*	[C/A]	341136	CDS_NON_SYNONYMOUS	Helix-loop-helix DNA-binding domain	CCTAGTTGGGATGTTGAAGAAAAT	ACCTTGAGGTTGTTGCATCTG	NA
SNP0275	Ca_24566	*Ca_Kabuli_Scaffold_50*	[C/T]	153631	CDS_NON_SYNONYMOUS	Protein of unknown function DUF3527	AGCCTAAATCATTCTTCTCCTGTG	TAAGCATGTGCCAGAAGAACTAAC	NA
SNP0276	Ca_27547	*Ca_Kabuli_Scaffold_53*	[A/G]	49273	CDS_NON_SYNONYMOUS	Zinc finger, CCCH-type	CAGGAAAAGTAAAATGGTCCAAAC	TTCTGAATCCTTTAGGGTTCTTTG	NA
SNP0277	Ca_23141	*Ca_Kabuli_Scaffold_562*	[C/A]	289080	CDS_NON_SYNONYMOUS	Protein of unknown function DUF642	GTTGCCTCCCTAATTATCTGCTT	CATCGCTATTTAACAACACCGTAA	NA
SNP0278	Ca_26833	*Ca_Kabuli_Scaffold_681*	[C/G]	18376	CDS_NON_SYNONYMOUS	Zinc finger, RING-type	CCTTGTGGTCACAGGTATATCAAA	AAACAAAAGGTTCGAGTAAAGCAC	NA
SNP0279	Ca_23185	*Ca_Kabuli_Scaffold_682*	[G/C]	157238	CDS_NON_SYNONYMOUS	Transcriptional factor B3	AATCCATAATCCCAAACTATGCTG	ATTCGACTTTGCATTTATGGAGTG	NA
SNP0280	Ca_25929	*Ca_Kabuli_Scaffold_753*	[A/G]	8312	CDS_NON_SYNONYMOUS	Zinc finger, Dof-type	TAAAAAGCCCGACAAAATACTCTC	AAGGAGATTCCTCTTTTGATGTTC	NA
SNP0281	Ca_22169	*Ca_Kabuli_Scaffold_88*	[C/T]	16398	CDS_NON_SYNONYMOUS	ZF-HD homeobox protein, Cys/His-rich dimerisation domain	GATCTGAGTCATCTAAATCGAGCA	GTCCACAAAATAGGGAACAATGTA	NA
SNP0282	Ca_28056	*Ca_Kabuli_Scaffold_C11164954*	[T/A]	5778	CDS_NON_SYNONYMOUS	Protein of unknown function DUF2930	TTGGTAATGCTCTTTAAGTCCACA	CGTGGAAGCCTATCTAAATTGTTC	NA
SNP0283	Ca_00488	*Ca_Desi_Chr01*	[C/A]	5704265	CDS_NON_SYNONYMOUS	Kinase-related protein of unknown function (DUF1296)	CAATGAACCCTATCCTTTGCTAGT	AGAGCCGACTAGGATTAGTAGCTG	NA
SNP0284	Ca_00882	*Ca_Desi_Chr01*	[G/C]	11411433	CDS_NON_SYNONYMOUS	MYB family transcription factor	TATGGAGAAGAAGGGTTTGTCTTC	CTCAAGGATGAAACGAAGAAACTT	NA
SNP0285	Ca_01546	*Ca_Desi_Chr02*	[A/C]	7082872	CDS_NON_SYNONYMOUS	ABSCISIC ACID-INSENSITIVE 5-like protein 2	CGATCTACAATCTTGTTGAAAACG	GAGATACTCACCGAAACCATCATT	NA
SNP0286	Ca_02202	*Ca_Desi_Chr03*	[T/G]	697967	CDS_NON_SYNONYMOUS	Protein LHY	TTGTCCAAGACTTATGGTTAGCAG	ACAAAAGACTGATGGCACTTGTAA	NA
SNP0287	Ca_02541	*Ca_Desi_Chr03*	[C/T]	4356981	CDS_NON_SYNONYMOUS	WD repeat domain phosphoinositide-interacting protein 3	TGCTCATGATTCAAGAATAGGTTG	ACATGAAAATACTCCAATGCACAG	NA
SNP0288	Ca_02786	*Ca_Desi_Chr03*	[A/G]	8826641	CDS_NON_SYNONYMOUS	Transcription elongation factor SPT6	CATGTCACCTTCATGTAGCCTATC	TTCCCTCTGATCTATCTTTCACCT	NA
SNP0289	Ca_03197	*Ca_Desi_Chr03*	[G/C]	15385992	CDS_NON_SYNONYMOUS	Zinc finger CCCH domain-containing protein 5	GTTGCATATGCTTGAAATCTCTTG	CTTTTGATTGTCACAACATCACTG	NA
SNP0290	Ca_03610	*Ca_Desi_Chr03*	[A/G]	19826899	CDS_NON_SYNONYMOUS	Bromodomain adjacent to zinc finger domain protein 1A	AGACAAACAGGGTTGTTGAGTACA	ATCTGTGTATCTCCCAAAAAGGAG	NA
SNP0291	Ca_04110	*Ca_Desi_Chr04*	[G/C]	3248872	CDS_NON_SYNONYMOUS	Transcriptional corepressor LEUNIG	ATATTTATTGCCAGGACAAACGAG	AACTCAACCTTACAAAACCAGCTT	NA
SNP0292	Ca_04363	*Ca_Desi_Chr04*	[C/T]	7647357	CDS_NON_SYNONYMOUS	Lysine-specific demethylase 3B	CAGAAAATCTAACCACAACTGCAT	TTTGGGATGTACTTACAGGGATCT	NA
SNP0293	Ca_04406	*Ca_Desi_Chr04*	[G/T]	8131383	CDS_NON_SYNONYMOUS	Transcriptional corepressor LEUNIG	CAAGTAACAAAGCCACGGACTAAT	GTATGGTTGATGACAACATGGAAT	NA
SNP0294	Ca_04679	*Ca_Desi_Chr04*	[C/T]	11399103	CDS_NON_SYNONYMOUS	NAC domain-containing protein 78	CAAGGACATCCAATGATATACGTC	TGAAAATCAGAGTTACCAGCTGAC	NA
SNP0295	Ca_05622	*Ca_Desi_Chr05*	[A/G]	5207791	CDS_NON_SYNONYMOUS	BEACH domain-containing protein lvsC	TTCATTCTTACGAGAGTTGTGGAA	CCATTAGAAAGCCACCTTAATTGT	NA
SNP0296	Ca_05724	*Ca_Desi_Chr05*	[G/C]	6320247	CDS_NON_SYNONYMOUS	Protein ETHYLENE INSENSITIVE 3	GTATATTTCCAGTTCCACCAGCTT	GGAAACCTGAGACTCTTCTTCATC	NA

**SNP IDs**	***Kabuli*/*desi* gene accession IDs**	***Kabuli*/*desi* chromosomes**	**SNPs**	**Physical positions (bp)**	**Sequence components of genome**	**Putative functions**	**Forward primer sequences (5'-3')**	**Reverse primer sequences (5'-3')**	**Primers used in Figure 2A**
SNP0297	Ca_05955	*Ca_Desi_Chr05*	[G/A]	8681026	CDS_NON_SYNONYMOUS	Two-component response regulator-like PRR95	ACTTCCCTGATCAACAAAGAGAAC	TTATCGAATCAGAGTGTGTCAGGT	NA
SNP0298	Ca_06336	*Ca_Desi_Chr05*	[G/A]	13173580	CDS_NON_SYNONYMOUS	Protein of unknown function (DUF399 and DUF3411)	GTCTACATCTGCATTTTGTCTGCT	ACTAATTTTGACATGACCCCACTT	NA
SNP0299	Ca_07258	*Ca_Desi_Chr06*	[A/C]	10171978	CDS_NON_SYNONYMOUS	Homeobox protein 10	GTTGAGGAACTTCTACAGGTAGCC	AGGTAATAAAACCTTTGGTCGTTG	NA
SNP0300	Ca_07873	*Ca_Desi_Chr07*	[C/A]	7266216	CDS_NON_SYNONYMOUS	SBP (S-ribonuclease binding protein) family protein	GGATAGAGAAGGAACAGAAACAGG	TTCTATATTTAACCCATCCGCTTC	NA
SNP0301	Ca_08579	*Ca_Desi_Chr08*	[T/G]	8124369	CDS_NON_SYNONYMOUS	Transcription factor MYB98	TGGCATTCACAGACTAGTTTCATT	GTCCATATGTCCTTCTGCAAAAAT	NA
SNP0302	Ca_08625	*Ca_Desi_Chr08*	[C/A]	8581939	CDS_NON_SYNONYMOUS	Scarecrow-like protein 28	ATTGTTGTCACCAAGTCACAGATT	CCATCATAAAGTGTCTTGTGAAGC	NA
SNP0303	Ca_15118	*Ca_Desi_Scaffold_1166*	[C/G]	34273	CDS_NON_SYNONYMOUS	Dof zinc finger protein DOF5.2	ACTTTACTTACTTGCTGGCCAAAC	GCTTGCCATTGAATGTCTAAATCT	NA
SNP0304	Ca_15434	*Ca_Desi_Scaffold_1290*	[C/A]	33887	CDS_NON_SYNONYMOUS	Transcription initiation factor TFIID subunit 7	GCAATTTAGTGAGGATGGAAGAAG	AACCCATCAAACACCTATTTTAGC	NA
SNP0305	Ca_15904	*Ca_Desi_Scaffold_1484*	[A/T]	25898	CDS_NON_SYNONYMOUS	BTB/POZ domain-containing protein NPY1	TCTATTTTAGGCAATAGGGCGATA	TATGAGATTAGCCGTGATTCTTTG	NA
SNP0306	Ca_16252	*Ca_Desi_Scaffold_1667*	[T/G]	33602	CDS_NON_SYNONYMOUS	Zinc finger CCCH domain-containing protein 53	CTCAGTCACCTTCACCTTTTCTTT	ATCTTGAACAGGTCCATAAATGCT	NA
SNP0307	Ca_16417	*Ca_Desi_Scaffold_1774*	[G/T]	44332	CDS_NON_SYNONYMOUS	Scarecrow-like protein 13	CGTTCTCCTACAATCTGAAGTCCT	GAGGGACCAGAAAGTTTGATCTTA	NA
SNP0308	Ca_08976	*Ca_Desi_Scaffold_233*	[C/A]	4551	CDS_NON_SYNONYMOUS	Transcription activator GLK1	GAGACATGAAAAAGTTCTCCACAA	ATTAGCTCGACAAAGTTCCTCAGT	NA
SNP0309	Ca_11984	*Ca_Desi_Scaffold_310*	[C/G]	49502	CDS_NON_SYNONYMOUS	Zinc finger CCCH domain-containing protein 48	CTCTCTTGCTTCTGAAACAGATCA	AAGCATCACAAATTGCACAGTTAC	NA
SNP0310	Ca_18179	*Ca_Desi_Scaffold_3151*	[T/C]	27219	CDS_NON_SYNONYMOUS	SET domain-containing protein	TGCCATTTCGATTTCGAGGTTG	TTAATGGAAAGCAGGAGAG	NA
SNP0311	Ca_10618	*Ca_Desi_Scaffold_32*	[G/T]	210202	CDS_NON_SYNONYMOUS	Probable WRKY transcription factor 19	GACGATGTATATCTCATGGAGGTG	AGAATACATGGCACACTACAGTCC	NA
SNP0312	Ca_18943	*Ca_Desi_Scaffold_4448*	[G/A]	15163	CDS_NON_SYNONYMOUS	Protein of unknown function (DUF810)	TGGTATTGCAGTTGTAGAGACCAT	ATCCCTCTCTCTTTTTCTCCTCAA	NA
SNP0313	Ca_12689	*Ca_Desi_Scaffold_450*	[C/G]	42711	CDS_NON_SYNONYMOUS	Transcription factor TCP7	GCACTCTCAAGTCTCAATCAGAAG	TAGAGGGGTGTGAGCTGTGTAATA	NA
SNP0314	Ca_12770	*Ca_Desi_Scaffold_473*	[G/C]	13637	CDS_NON_SYNONYMOUS	SWI/SNF complex subunit SWI3C	TAAAACTGCACCATTCTAGTTCCA	CTTTTGATGCGATTCTTACACATC	NA
SNP0315	Ca_19525	*Ca_Desi_Scaffold_5938*	[G/C]	6461	CDS_NON_SYNONYMOUS	Transcriptional corepressor LEUNIG	TACCCATTCATTATGTCCAACAAG	CTCATTACTGTTTCATGGTTTGGA	NA
SNP0316	Ca_13464	*Ca_Desi_Scaffold_624*	[A/G]	69711	CDS_NON_SYNONYMOUS	WRKY transcription factor 6	AAATATGCGTACTTTCCTGAATCC	ATTAATTTGATACCGTGAGGTTGC	NA
SNP0317	Ca_13667	*Ca_Desi_Scaffold_680*	[G/A]	76290	CDS_NON_SYNONYMOUS	Protein of unknown function (DUF604)	ATGAGCATTCACCAATGAGTACAG	CTCTGCATGCTATCTATCTCCTCA	NA
SNP0318	Ca_14026	*Ca_Desi_Scaffold_782*	[A/C]	33500	CDS_NON_SYNONYMOUS	Unknown expressed protein	TAGCATATGAGTGAGCATTTAGGG	CAGAATGTGCAGTTTCTAAGGCTA	NA
SNP0319	Ca_00096	*Ca_Kabuli_Chr01*	[G/A]	777240	CDS_SYNONYMOUS	Tubby, C-terminal	CGGCTACAATAAAGCTGGTTATTC	GCAACTGTTATGACCAAAGGTGTA	NA
SNP0320	Ca_00149	*Ca_Kabuli_Chr01*	[T/C]	1215024	CDS_SYNONYMOUS	BTB/POZ-like	GCGAACCGAATTTGAATAAGATAG	GAGTTTTTGACAGTCCATTACACG	NA
SNP0321	Ca_00180	*Ca_Kabuli_Chr01*	[A/G]	1467056	CDS_SYNONYMOUS	Zinc finger, RING-type	ACTAGATACATTGCCGTCAAACAA	GACCACATAAGGAATTGAACACAA	NA
SNP0322	Ca_00224	*Ca_Kabuli_Chr01*	[T/C]	1784006	CDS_SYNONYMOUS	Zinc finger, RING-type	GTCTTTTTATTCCAAAGCTGCAAG	TATTCGTGTAACCGAACAAAGATG	NA
SNP0323	Ca_00347	*Ca_Kabuli_Chr01*	[G/A]	2832969	CDS_SYNONYMOUS	Transcriptional factor B3	ATCTCAGGTTCAAACCTTAACCAC	AGGAAATATCAGAATGCCACAAGT	NA

SNP IDs	*Kabuli* /*desi* gene accession IDs	*Kabuli* /*desi* chromosomes	SNPs	Physical positions (bp)	Sequence components of genome	Putative functions	Forward primer sequences (5'-3')	Reverse primer sequences (5'-3')	Primers used in Figure 2A
SNP0324	Ca_00431	*Ca_Kabuli_Chr01*	[A/T]	3544948	CDS_SYNONYMOUS	Zinc finger, FYVE-type	ATTATTGAAAAGATGGAGGTGTGC	GGTATTACTTATGCAACATGTCACG	NA
SNP0325	Ca_00467	*Ca_Kabuli_Chr01*	[T/C]	3853408	CDS_SYNONYMOUS	AUX/IAA protein	GTGGCAATGTAATAGCATTTTGAG	TTGCGTTTTAGCTGTGAAGTTTAG	NA
SNP0326	Ca_00512	*Ca_Kabuli_Chr01*	[T/C]	4302751	CDS_SYNONYMOUS	Zinc finger, CCCH-type	GTTGCATATGCTTGAAATCTCTTG	CTTTTGATTGTCACAACATCACTG	NA
SNP0327	Ca_00536	*Ca_Kabuli_Chr01*	[T/C]	4512566	CDS_SYNONYMOUS	Tubby, C-terminal	ATTGGGTCTACTTCAACCCTACAA	CTCTGTGGAAAATTGGTATTTTGG	NA
SNP0328	Ca_00562	*Ca_Kabuli_Chr01*	[C/T]	4781362	CDS_SYNONYMOUS	Zinc finger, RING-type	AACATCATGGCACTACTCACTCAT	AGCAGCACTAAGAGGGACTACATT	NA
SNP0329	Ca_00613	*Ca_Kabuli_Chr01*	[A/C]	5249846	CDS_SYNONYMOUS	Protein of unknown function DUF584	AAGTGCTTCACGTCATGATTGTAT	TGAGTTGGACTCTCTTCAGACAAG	NA
SNP0330	Ca_00628	*Ca_Kabuli_Chr01*	[A/C]	5430700	CDS_SYNONYMOUS	Protein of unknown function DUF584	GACCAACTATTTCAATACGAACGA	CAACCACCAAGTGGAATATGATTA	NA
SNP0331	Ca_00656	*Ca_Kabuli_Chr01*	[A/C]	5666178	CDS_SYNONYMOUS	DNA-binding WRKY	CTAAGCGGGACAGATGTAATTTTG	GAACACATAACCATCCACTACCAC	NA
SNP0332	Ca_07916	*Ca_Kabuli_Chr01*	[C/T]	6521670	CDS_SYNONYMOUS	Protein of unknown function DUF827, plant	CTGAACAATTCAAGGTAGCCACTA	AGCGTCATAATCATTGTATGTGGT	NA
SNP0333	Ca_07932	*Ca_Kabuli_Chr01*	[A/G]	6679253	CDS_SYNONYMOUS	WD40 repeat	TTTTAGAGACCACCTCAACAAGTG	TGGAATGACTCTGTCATAGCTGTT	NA
SNP0334	Ca_07994	*Ca_Kabuli_Chr01*	[G/A]	7417699	CDS_SYNONYMOUS	Homeobox	CATCATCATCATTGAGAACTTTCC	ATACGATTGAGCACGAAGAGGTAT	NA
SNP0335	Ca_07995	*Ca_Kabuli_Chr01*	[C/T]	7439421	CDS_SYNONYMOUS	CCAAT-binding transcription factor, subunit B	CAGTTCAACATGGAGAAGAAGGTA	TACACATGACCCCACAGGTATTTA	NA
SNP0336	Ca_02909	*Ca_Kabuli_Chr01*	[A/G]	8942198	CDS_SYNONYMOUS	Zinc finger, UBP-type	GAATATCTTTAGTGCCTGCCAGAT	CAATGAATTGCTTGCTACTCAACT	NA
SNP0337	Ca_02837	*Ca_Kabuli_Chr01*	[T/A]	9603896	CDS_SYNONYMOUS	WD40 repeat	CATCCACTACAACAGCCTCTTCTT	ATGACCTCCTAGTCACTCCAACTC	NA
SNP0338	Ca_02827	*Ca_Kabuli_Chr01*	[A/C]	9705162	CDS_SYNONYMOUS	Domain of unknown function DUF632	TGTCAGCAAAATTGGAGTGATAGT	CTTGTCAAGAAGAAGGGGATTTAT	NA
SNP0339	Ca_02741	*Ca_Kabuli_Chr01*	[G/A]	10366383	CDS_SYNONYMOUS	Lateral organ boundaries, LOB	TGTTGTTGTGGTTGTGACATACTC	GTTCGCATGCATTCACTTTATTAC	NA
SNP0340	Ca_02713	*Ca_Kabuli_Chr01*	[T/G]	10560690	CDS_SYNONYMOUS	CCAAT-binding transcription factor, subunit B	AATCAAGCAGTGGTCTTCATAACA	TTATCAGAGCTACGTCCCCATATT	NA
SNP0341	Ca_02692	*Ca_Kabuli_Chr01*	[T/G]	10710672	CDS_SYNONYMOUS	Basic-leucine zipper (bZIP) transcription factor	ACTTCAACTTTACGCTGCTTTCTT	TTTAACAACTTGACCGGTGACTAC	NA
SNP0342	Ca_02626	*Ca_Kabuli_Chr01*	[T/A]	11277703	CDS_SYNONYMOUS	Transcription factor GRAS	GGTGCACTATAACTTCCATTGACA	CACCTTCTTCAATCATAAGCTCAA	NA
SNP0343	Ca_02625	*Ca_Kabuli_Chr01*	[G/A]	11285192	CDS_SYNONYMOUS	Transcription factor GRAS	AGCATCCAGTTGTTATCTTCATCA	AAACTCAGCTTTTTACTGGTTTGG	NA
SNP0344	Ca_07131	*Ca_Kabuli_Chr01*	[T/G]	13991356	CDS_SYNONYMOUS	Homeobox	AATCTGGCAGTGAAAATCAAGAAG	TGTCATTGCTCTTGAAACAATACC	NA
SNP0345	Ca_07113	*Ca_Kabuli_Chr01*	[G/T]	14225210	CDS_SYNONYMOUS	DNA-binding WRKY	TTACAAAACTTCACAGAGGGTGAG	ATGATATTGGTGTGGTGCTTACAA	NA
SNP0346	Ca_07091	*Ca_Kabuli_Chr01*	[T/C]	14459908	CDS_SYNONYMOUS	Helix-loop-helix DNA-binding domain	AGCTGGTAGCTCGAATCAAATATC	GTTCACCTTTGGAACTTCACCTAT	NA
SNP0347	Ca_07050	*Ca_Kabuli_Chr01*	[A/G]	14801406	CDS_SYNONYMOUS	Zinc finger, AN1-type	CCTTAACAAATCGGATATCAACTAC	TCTCAATTTCTCTCGTGTATCTGC	NA
SNP0348	Ca_07018	*Ca_Kabuli_Chr01*	[T/C]	15167641	CDS_SYNONYMOUS	Protein of unknown function DUF3411	CATACGGGTTATTCAACATGTCTC	CAACATCTGATCCACCACTAAAAC	NA
SNP0349	Ca_07002	*Ca_Kabuli_Chr01*	[T/C]	15319181	CDS_SYNONYMOUS	Zinc finger, PHD-type	TAGCCTCTTCAAAGGATGTAGGTC	GCACTCAAGCGAAACATATGATAA	NA
SNP0350	Ca_06945	*Ca_Kabuli_Chr01*	[G/A]	16061541	CDS_SYNONYMOUS	BTB/Kelch-associated	TATCAACTACCCACCAAAAGGATT	AGTTGATTTTGGGTTTGCTCTAGT	NA

**SNP IDs**	***Kabuli*/*desi* gene accession IDs**	***Kabuli*/*desi* chromosomes**	**SNPs**	**Physical positions (bp)**	**Sequence components of genome**	**Putative functions**	**Forward primer sequences (5'-3')**	**Reverse primer sequences (5'-3')**	**Primers used in Figure 2A**
SNP0351	Ca_06943	*Ca_Kabuli_Chr01*	[T/C]	16081438	CDS_SYNONYMOUS	Transcription factor GRAS	GCATAAGACAACTGATTCATCACC	GACGTTAATTCTTTCCTTGCTGTT	NA
SNP0352	Ca_06913	*Ca_Kabuli_Chr01*	[G/A]	16512171	CDS_SYNONYMOUS	Protein of unknown function DUF3411	AAAACTACCGCAAGGACTATCAAG	GCTTGGAAGTCTCTCCTAATTGAA	NA
SNP0353	Ca_06901	*Ca_Kabuli_Chr01*	[G/A]	16636587	CDS_SYNONYMOUS	No apical meristem (NAM) protein	GATGACAAAGTAGCAACGGTATTG	GGCCTGAATTCTAGTTAACGTCAT	NA
SNP0354	Ca_06894	*Ca_Kabuli_Chr01*	[C/T]	16743569	CDS_SYNONYMOUS	Protein of unknown function DUF1218	TACCATCAACACTCCTATGCATTC	AACTGTGAGTTACATTGACCAGGA	NA
SNP0355	Ca_06867	*Ca_Kabuli_Chr01*	[T/C]	17006391	CDS_SYNONYMOUS	Protein of unknown function DUF616	TCTATAGTGTCTCGAAATGGCAAA	GTTATGTTCCTGTGAAGCAAGCTA	NA
SNP0356	Ca_22780	*Ca_Kabuli_Chr01*	[T/C]	17314945	CDS_SYNONYMOUS	WD40 repeat	CTTCCATATCTGTTCAAGCCTTTT	CTATCTCATGAGTTGGTTGGTCTG	NA
SNP0357	Ca_22781	*Ca_Kabuli_Chr01*	[G/A]	17328721	CDS_SYNONYMOUS	Bromodomain	TGACCCAAGTAATGAGCAGAGTAA	TGCCTAGACCTTAATACTCGCCTA	NA
SNP0358	Ca_14778	*Ca_Kabuli_Chr01*	[T/G]	17576793	CDS_SYNONYMOUS	Protein of unknown function DUF789	AGGTTGGATATGTCGCTATCTTTC	GTCTCCTTGTCTGCTATTCAGCTT	NA
SNP0359	Ca_18326	*Ca_Kabuli_Chr01*	[A/C]	24828245	CDS_SYNONYMOUS	ARID/BRIGHT DNA-binding domain	GAACTCCTACAAACCTAGGGATCA	TATTTTAGGTGGTTTCGAGAGGAG	NA
SNP0360	Ca_18325	*Ca_Kabuli_Chr01*	[A/C]	24845148	CDS_SYNONYMOUS	Zinc finger, RING-type	CTAAACGACATCAAACTCGAGAGA	CCTGAGGCTTAAACCAAATAAAAG	NA
**SNP0361**	**Ca_18591**	***Ca_Kabuli_Chr01***	**[C/T]**	**26026817**	**CDS_SYNONYMOUS**	**Transcription factor, SBP-box**	**TGTCGTACACTAGACACACCTTCA**	**TACTTGTCTGTGGTGGACTTGACT**	NA
SNP0362	Ca_21419	*Ca_Kabuli_Chr01*	[T/G]	34727251	CDS_SYNONYMOUS	Zinc finger, CCCH-type	ATCCAATGTTTCTGTTGAAGTTCC	CAAAAAGTGGATCATATTGCTCAG	NA
SNP0363	Ca_24110	*Ca_Kabuli_Chr01*	[C/T]	35708841	CDS_SYNONYMOUS	Mediator complex, subunit Med10	AGATGAGGATACCGTAAAAATTGG	AACGTGTGCTTAAATTGTGATGAG	NA
SNP0364	Ca_23534	*Ca_Kabuli_Chr01*	[G/A]	37722095	CDS_SYNONYMOUS	Protein of unknown function DUF620	AGGTCGATGTTACATTCAACACAT	TAGAGTTCAGCACAAATGGAAGAA	NA
SNP0365	Ca_23539	*Ca_Kabuli_Chr01*	[T/G]	37862342	CDS_SYNONYMOUS	Zinc finger, PHD-type	AAAGATGAGCTTGAAAGAGAAGGA	GTTGAATTGAATAAGACGGCTACC	NA
SNP0366	Ca_13548	*Ca_Kabuli_Chr01*	[C/T]	43800367	CDS_SYNONYMOUS	BTB/POZ-like	CATGACACTACTTTTGCAAGGTTT	GAAACTCATTGGTGCATATTCTTG	NA
SNP0367	Ca_16982	*Ca_Kabuli_Chr02*	[T/C]	354795	CDS_SYNONYMOUS	Lateral organ boundaries, LOB	TTCTCTCCTATATATTCGCCTCCA	AGACATCACCACATTCACATTTTC	NA
SNP0368	Ca_12605	*Ca_Kabuli_Chr02*	[A/G]	1797399	CDS_SYNONYMOUS	Domain of unknown function DUF3730	TTCGTCAATTTAGTCTCTGCTGTT	ATGCTCAAGTTCTGATTGAACAAG	NA
SNP0369	Ca_12602	*Ca_Kabuli_Chr02*	[T/C]	1828318	CDS_SYNONYMOUS	Protein of unknown function DUF1421	AGTCAACTGACTGACCATTCTCTG	GGTCAACACTTGAGACTACTCAGC	NA
SNP0370	Ca_10514	*Ca_Kabuli_Chr02*	[C/A]	3450904	CDS_SYNONYMOUS	ZF-HD homeobox protein, Cys/His-rich dimerisation domain	AATTGAACTTGGAGAAGAGAAACG	TTAACAAGTTCGAAATCACCAGAG	NA
SNP0371	Ca_10463	*Ca_Kabuli_Chr02*	[A/G]	3930182	CDS_SYNONYMOUS	Zinc finger, PHD-type	TCTTCTTGATTGAATCTCTCGTCA	TGTTAGCTTTCGTGTTCAGTTAGG	NA
SNP0372	Ca_20383	*Ca_Kabuli_Chr02*	[G/A]	10025451	CDS_SYNONYMOUS	Protein of unknown function DUF810	AAGGTCAGGTTCTATCTGTCCAGT	CCCTTGTTGGTCAAGTAAGACTTT	NA
SNP0373	Ca_11686	*Ca_Kabuli_Chr02*	[T/G]	13079445	CDS_SYNONYMOUS	Protein of unknown function DUF3110	AAGCTTGACAAACTCTTTTCCTGT	GACATAAATCAAATGTCGAACCAG	NA
SNP0374	Ca_11681	*Ca_Kabuli_Chr02*	[T/C]	13217781	CDS_SYNONYMOUS	Zinc finger, DHHC-type, palmitoyltransferase	AGCTCAAGCTCATTTCATCAGTTA	CACAATTATGGGGTTAGAATAGGC	NA
SNP0375	Ca_18564	*Ca_Kabuli_Chr02*	[C/A]	15234951	CDS_SYNONYMOUS	Protein of unknown function DUF1675	GACTATGTCGACACCGATTCATAC	GTTCAACTGGTGGCACTGATAATA	NA
SNP0376	Ca_14972	*Ca_Kabuli_Chr02*	[C/T]	23181353	CDS_SYNONYMOUS	Protein of unknown function DUF1423, plant	TCTGTTTGAAGGTCAGCTATTGAG	TAGAAACTATCCCCAGCCACTAAG	NA
SNP0377	Ca_14280	*Ca_Kabuli_Chr02*	[C/A]	25508579	CDS_SYNONYMOUS	Zinc finger, CCCH-type	TCTATCTTCAATCCAAGGAAGAGG	GTATTGCAGTTTCCTTTGCTGATA	NA

SNP IDs	*Kabuli* /*desi* gene accession IDs	*Kabuli* /*desi* chromosomes	SNPs	Physical positions (bp)	Sequence components of genome	Putative functions	Forward primer sequences (5'-3')	Reverse primer sequences (5'-3')	Primers used in Figure 2A
SNP0378	Ca_15674	*Ca_Kabuli_Chr02*	[G/T]	27724425	CDS_SYNONYMOUS	BTB/POZ-like	AGTAGTTCCAGTTGATTCGTTTTG	GGAGAGAACTGGACAATGGTAAAC	NA
SNP0379	Ca_15694	*Ca_Kabuli_Chr02*	[A/G]	28017163	CDS_SYNONYMOUS	AUX/IAA protein	TGGAATCGGAAGGAATACTTTATG	TTATCGACTAATATCCTGCAACCA	NA
SNP0380	Ca_16122	*Ca_Kabuli_Chr02*	[A/G]	29693107	CDS_SYNONYMOUS	Lateral organ boundaries, LOB	TCCATGTATGAGTTGAAACTTTGG	GATAATGACAATGTAGGCCACAAA	NA
SNP0381	Ca_12474	*Ca_Kabuli_Chr02*	[G/A]	30867547	CDS_SYNONYMOUS	Zinc finger, CCCH-type	GATCTGATTGTTCCTGCCTTTAAT	CCTTAACCAATGAATTAACCCAAG	NA
SNP0382	Ca_12464	*Ca_Kabuli_Chr02*	[C/T]	31005845	CDS_SYNONYMOUS	Lateral organ boundaries, LOB	GATGTCGTACCAAATAGGACATGA	CACCTAGGAATGATATCACACACG	NA
SNP0383	Ca_16883	*Ca_Kabuli_Chr02*	[C/A]	34847681	CDS_SYNONYMOUS	Bromodomain	TAATCCTCTCCTATCCAAAAGGTG	ACCCAGAGAGATGGTACCAAGTAA	NA
SNP0384	Ca_09731	*Ca_Kabuli_Chr02*	[C/A]	35612587	CDS_SYNONYMOUS	Protein of unknown function DUF827, plant	AAGCAAACAGCAGAAGCTAAAGAT	TGACAAAAATCTGATCACATAGGC	NA
SNP0385	Ca_09786	*Ca_Kabuli_Chr02*	[A/T]	36088577	CDS_SYNONYMOUS	Transcription factor GRAS	CAATTCTCCACTCAAAGTTCCTCT	AAGCAACCAAATCTCTATTCATCC	NA
SNP0386	Ca_09827	*Ca_Kabuli_Chr02*	[A/G]	36375224	CDS_SYNONYMOUS	Domain of unknown function DUF632	TAATGGCTATGCATTCAGTGACTT	TGACACCAGTGTCCTAATTTGACT	NA
SNP0387	Ca_22090	*Ca_Kabuli_Chr03*	[T/C]	4823828	CDS_SYNONYMOUS	DNA-binding WRKY	CAAAGAAGATCAATCTTGTGATGC	TCCAATTCTGTGTAACAACATCCT	NA
SNP0388	Ca_16550	*Ca_Kabuli_Chr03*	[C/T]	6840644	CDS_SYNONYMOUS	WD40 repeat	CAACCTAACAAGTAATGGATGCAA	TTCAGAGATGGAGTTTGTTCATGT	NA
SNP0389	Ca_16540	*Ca_Kabuli_Chr03*	[G/A]	7024620	CDS_SYNONYMOUS	Zinc finger, ZZ-type	CTGATTTTCGTAAAAGTCCCTTTC	CTCCTCCTCCTTCACATCTTCTAA	NA
SNP0390	Ca_19383	*Ca_Kabuli_Chr03*	[G/A]	11678625	CDS_SYNONYMOUS	Homeobox	GACACCTATACAACACCCACACAT	CAAGTAGAGTGCTTGTTTCCAAGA	NA
SNP0391	Ca_21021	*Ca_Kabuli_Chr03*	[A/C]	12599496	CDS_SYNONYMOUS	Zinc finger, Dof-type	AGTGATGAACCATTACCAAGTTGA	TAAGAAACACGTTTTGTGCTCTTC	NA
SNP0392	Ca_22696	*Ca_Kabuli_Chr03*	[T/C]	13420776	CDS_SYNONYMOUS	Zinc finger, C2H2-type	GCTTTTCTGGGACTTGGATAATAA	CACCTTTTTCTTTCCCTTGTACTC	NA
SNP0393	Ca_21130	*Ca_Kabuli_Chr03*	[T/C]	17936321	CDS_SYNONYMOUS	SANT domain, DNA binding	TAACTGACATGCTGAACTTGGTCT	GAAAGGTGGAAGAATTACCTCAAG	NA
SNP0394	Ca_19620	*Ca_Kabuli_Chr03*	[T/C]	18355427	CDS_SYNONYMOUS	Protein of unknown function DUF3411	ACTATGTGTGAACCTGCATTGTCT	CAAATGTCAACTATGGCTCTCTTG	NA
SNP0395	Ca_09406	*Ca_Kabuli_Chr03*	[T/C]	20030262	CDS_SYNONYMOUS	Protein of unknown function DUF688	ACCATGTATACTTGACCACCCTTT	CCAATGGGTCTTGCTAAAGAATTA	NA
SNP0396	Ca_09494	*Ca_Kabuli_Chr03*	[C/T]	21335892	CDS_SYNONYMOUS	Protein of unknown function DUF599	CAATGTCGTGTCTTTGATCTTCTC	ACGGATTTTAATCCTCTATTGCAC	NA
SNP0397	Ca_20431	*Ca_Kabuli_Chr03*	[A/G]	21950186	CDS_SYNONYMOUS	Domain of unknown function DUF296	TAGTCTACACTGATGGTGCATGTC	GAGCTTACACCGTCAAACTTAACA	NA
SNP0398	Ca_20412	*Ca_Kabuli_Chr03*	[T/C]	22169816	CDS_SYNONYMOUS	BEACH domain	GCGAAATCCAGTTTTCTATCTCTC	AAGATGCGTTCTGACCTAGATACC	NA
SNP0399	Ca_20411	*Ca_Kabuli_Chr03*	[T/C]	22188124	CDS_SYNONYMOUS	Domain of unknown function DUF1088	GAACAAAGTCCCAAAATAAGAGGA	GGGAACCCCTATAGATGTATGCTT	NA
SNP0400	Ca_06189	*Ca_Kabuli_Chr03*	[T/A]	23273057	CDS_SYNONYMOUS	SNF2-related	GTTGTGGATTGTAACCAAACAAGA	CACCTAAGTTCATGTTTGCAGAAG	NA
SNP0401	Ca_06124	*Ca_Kabuli_Chr03*	[G/A]	23966110	CDS_SYNONYMOUS	DNA-binding WRKY	TGTTTGCAATTACTATGAGGTGGT	TTCTCATCCCTCTTCTAGGAGCTA	NA
SNP0402	Ca_23882	*Ca_Kabuli_Chr03*	[C/T]	25757693	CDS_SYNONYMOUS	SET domain	TCATAATTGCCATCCTGAAGTAAG	AGTTATCCAATTCAAGCATGTCCT	NA
SNP0403	Ca_08296	*Ca_Kabuli_Chr03*	[T/G]	25987920	CDS_SYNONYMOUS	Protein of unknown function DUF677	AAATGAAACCTCTCTTGGGTTGTA	GAATACGATCTAGCAGTTCAAGCA	NA
SNP0404	Ca_08199	*Ca_Kabuli_Chr03*	[A/G]	27047840	CDS_SYNONYMOUS	BTB/POZ-like	TTTTAGCCCCCAATCTAACTGTAG	AAGGAAAATGCTAAGGGAAGAACT	NA

SNP IDs	*Kabuli* /*desi* gene accession IDs	*Kabuli* /*desi* chromosomes	SNPs	Physical positions (bp)	Sequence components of genome	Putative functions	Forward primer sequences (5'-3')	Reverse primer sequences (5'-3')	Primers used in Figure 2A
SNP0405	Ca_08186	*Ca_Kabuli_Chr03*	[C/T]	27194033	CDS_SYNONYMOUS	Zinc finger, Dof-type	ATATGAAGGAAAAGGAACCGAAAG	AACATCTCCTGTGTGAATCCAATA	NA
SNP0406	Ca_07151	*Ca_Kabuli_Chr03*	[C/T]	28374024	CDS_SYNONYMOUS	Transcription elongation factor, TFIIS/CRSP70, N-terminal, sub-type	TCGACGATATATAACTCGGGAAAT	TATGTTGACTTTCTAGTGGCTGGA	NA
SNP0407	Ca_07159	*Ca_Kabuli_Chr03*	[C/T]	28478044	CDS_SYNONYMOUS	Helix-loop-helix DNA-binding domain	GATGTATACGGTGTTTGAGAGTGG	TTGACAGTACTCCTGCAACTCTGT	NA
SNP0408	Ca_07170	*Ca_Kabuli_Chr03*	[A/G]	28614784	CDS_SYNONYMOUS	Basic-leucine zipper (bZIP) transcription factor	ACCACAAACCTCTCCAAGTAGAAC	AGAGATATCTGCTTTTTGCTTTGG	NA
SNP0409	Ca_07176	*Ca_Kabuli_Chr03*	[T/A]	28706106	CDS_SYNONYMOUS	BTB/POZ-like	TACTCGGATCAGCACATATGAGAG	CACCAAGGTTAAGATAAACCAACC	NA
SNP0410	Ca_07274	*Ca_Kabuli_Chr03*	[C/A]	29690732	CDS_SYNONYMOUS	Zinc finger, AN1-type	AAGGAATGCTGTCAGTTCTCTTTT	ATACTGCACGTGATTTGTGAATG	NA
SNP0411	Ca_07288	*Ca_Kabuli_Chr03*	[C/T]	29803866	CDS_SYNONYMOUS	Protein of unknown function DUF2048	GACCTTCCTGTTTTGTTTCAATCT	ATAATGTACAACCCCTTCCCTCTT	NA
SNP0412	Ca_07299	*Ca_Kabuli_Chr03*	[A/G]	29898396	CDS_SYNONYMOUS	Transcription factor, SBP-box	TTCCTTTACAAGTGCTCCATGATA	AGACAGAAAAGTTGGCAGAGAGAG	NA
SNP0413	Ca_07307	*Ca_Kabuli_Chr03*	[A/G]	29974532	CDS_SYNONYMOUS	Zinc finger, CCCH-type	TTGTTTGAAGCTGATCTGTTTGTC	GTTTCAAATGATAATAGGCCTTGG	NA
SNP0414	Ca_12181	*Ca_Kabuli_Chr03*	[A/G]	30837147	CDS_SYNONYMOUS	Domain of unknown function DUF828	ATCTTAAGCCAACCTCATGCTAAC	TTCCATACACACGCTTAGACATTT	NA
SNP0415	Ca_12224	*Ca_Kabuli_Chr03*	[C/T]	31279502	CDS_SYNONYMOUS	Protein of unknown function DUF647	CCAATAGTGGCTATTTGACAACAC	TTATTCACCTCCTTGCTTGTGATA	NA
SNP0416	Ca_12234	*Ca_Kabuli_Chr03*	[T/C]	31341323	CDS_SYNONYMOUS	Domain of unknown function DUF640	CATGTTATTCCCTCATTTTTACCC	AAAGGATTAGCTTCTGGTTTTCCT	NA
SNP0417	Ca_12236	*Ca_Kabuli_Chr03*	[A/T]	31357787	CDS_SYNONYMOUS	Transcription factor jumonji/aspartyl beta-hydroxylase	ACCAAAATGTAGGAGCATTAGAGG	GCCTATCAAAGAAGTGGGATCTTA	NA
SNP0418	Ca_12258	*Ca_Kabuli_Chr03*	[G/A]	31591065	CDS_SYNONYMOUS	SET domain	AAAAGTTGAACGTAGAGCAGACCT	TGACCTAACAAAGGGAGAAGAAAA	NA
SNP0419	Ca_00791	*Ca_Kabuli_Chr03*	[G/T]	35185001	CDS_SYNONYMOUS	Basic-leucine zipper (bZIP) transcription factor	TATGTCTGGTGCATACAGTTGTGA	TGTTGTTTTTGAAAGTACCAGTGC	NA
SNP0420	Ca_00881	*Ca_Kabuli_Chr03*	[G/C]	35898320	CDS_SYNONYMOUS	WD40 repeat	AAATGTGGCTGAAGTTGAGTGATA	TTATTCATCATACTATGGCGCTGT	NA
SNP0421	Ca_00936	*Ca_Kabuli_Chr03*	[C/A]	36260865	CDS_SYNONYMOUS	BTB/POZ-like	CACCTTTACAAGAATTTGATGCTG	TTTGATTTGTGGTGTAGAATGCTC	NA
SNP0422	Ca_00939	*Ca_Kabuli_Chr03*	[A/G]	36293589	CDS_SYNONYMOUS	Zinc finger, CW-type	CTTGGACACTTTCAGAAGGATTTT	ATCTCCCAGAAGATTGTCTTTCAC	NA
SNP0423	Ca_00942	*Ca_Kabuli_Chr03*	[T/C]	36330815	CDS_SYNONYMOUS	Homeobox	ACAATATGGCTTACTTTGCTGACA	CACTGGACCTGGAAAATAAGAATC	NA
SNP0424	Ca_00947	*Ca_Kabuli_Chr03*	[A/T]	36387615	CDS_SYNONYMOUS	WD40 repeat	TGCTCCTTCAACTTTCTTTTAACC	TTTGTCATTTCCTCACATCATACC	NA
SNP0425	Ca_01033	*Ca_Kabuli_Chr03*	[T/G]	37067095	CDS_SYNONYMOUS	Zinc finger, RING-type	TGGTATTGCTAGTTTCCAAAGACA	CCCGTCCTCTTTATTTTGATTAGA	NA
SNP0426	Ca_01036	*Ca_Kabuli_Chr03*	[A/G]	37101409	CDS_SYNONYMOUS	Transcription factor, SBP-box	ATGAAAAATCCAGACCAAGAACTC	CCAATTTTACTCGCACACTTCATA	NA
SNP0427	Ca_01047	*Ca_Kabuli_Chr03*	[G/T]	37160822	CDS_SYNONYMOUS	Zinc finger, Sec23/Sec24-type	CAAGAGTCAACAATTTTCACCATC	CAGACATTCTGGAATCTGAAACTG	NA
SNP0428	Ca_01064	*Ca_Kabuli_Chr03*	[G/A]	37303478	CDS_SYNONYMOUS	WD40 repeat	GCGTTTAGTCATTTGCTCTGTTTA	TATTGAACTAGCCAACAAAGTGGA	NA
SNP0429	Ca_01180	*Ca_Kabuli_Chr03*	[G/T]	38315560	CDS_SYNONYMOUS	Zinc finger, RING-type	GCTACTATTCACCTTTGGCTTTGT	CAAACATGTCTCCATATTTTACGG	NA
SNP0430	Ca_01208	*Ca_Kabuli_Chr03*	[G/A]	38555941	CDS_SYNONYMOUS	WD40 repeat	CAGAACACTTCCAGTGCAATTTAC	TAGCACACTCGTTTCTTGTTCTTC	NA
SNP0431	Ca_01236	*Ca_Kabuli_Chr03*	[A/G]	38771817	CDS_SYNONYMOUS	Mediator complex, subunit Med15	GGAGAAGGGGGAAAATGATATAAG	TACTTCAGTGGCAATAAGAAACCA	NA

SNP IDs	*Kabuli* /*desi* gene accession IDs	*Kabuli* /*desi* chromosomes	SNPs	Physical positions (bp)	Sequence components of genome	Putative functions	Forward primer sequences (5'-3')	Reverse primer sequences (5'-3')	Primers used in Figure 2A
SNP0432	Ca_01271	*Ca_Kabuli_Chr03*	[A/C]	39084979	CDS_SYNONYMOUS	Domain of unknown function DUF296	TCAATGGCAGTAGAAAGCATTAAC	ATCTAACAAATGGCAAGAGGTTTG	NA
SNP0433	Ca_01317	*Ca_Kabuli_Chr03*	[A/C]	39481371	CDS_SYNONYMOUS	Protein of unknown function DUF2043	CTGAACACTCAACTCAGCAATTTT	ATATGCTAATGCTACCGGTTCTGT	NA
SNP0434	Ca_01368	*Ca_Kabuli_Chr03*	[G/A]	39817122	CDS_SYNONYMOUS	Transcriptional factor B3	GTAGGTGATGAGTGAGGTCAACTG	GATAGTAGAATTGATCGGGAGGAG	NA
SNP0435	Ca_27693	*Ca_Kabuli_Chr04*	[A/G]	20297	CDS_SYNONYMOUS	Basic-leucine zipper (bZIP) transcription factor	TTAATCCTTCACAAATGCGTAGAC	GACAGAAATACCATCTCGGAGTTT	NA
SNP0436	Ca_07691	*Ca_Kabuli_Chr04*	[C/G]	595982	CDS_SYNONYMOUS	Helix-loop-helix DNA-binding domain	GCCAAACTATGTTAACTCACACCA	AGTTCAGATCAACACAAACAGAGG	NA
SNP0437	Ca_07738	*Ca_Kabuli_Chr04*	[G/T]	1047639	CDS_SYNONYMOUS	SNF2-related	CTTGCCAAGTCCCAACTATAGAAT	TCAGAGGCATTAGACTTCAGAGTG	NA
SNP0438	Ca_07779	*Ca_Kabuli_Chr04*	[T/C]	1493927	CDS_SYNONYMOUS	DNA-binding WRKY	GGCACCTTAATTGATCTCTTCACT	AATTCTCAAGGGAACAACAAAGTC	NA
SNP0439	Ca_07797	*Ca_Kabuli_Chr04*	[C/T]	1708370	CDS_SYNONYMOUS	BEACH domain	ACCAAAAGATCATTACATCCATCC	TTCAAGCATCTTCTGGTACATAGG	NA
SNP0440	Ca_07804	*Ca_Kabuli_Chr04*	[T/C]	1833570	CDS_SYNONYMOUS	Domain of unknown function DUF828	CTCTTGATTCTGTCTTCAATCCAA	GAAAAATCACAGAACACAGATTGC	NA
SNP0441	Ca_12126	*Ca_Kabuli_Chr04*	[T/C]	3264351	CDS_SYNONYMOUS	Domain of unknown function DUF640	ATACGAGTCTTCAGCACAACATTT	AGGTGTTGCCTTGTAGGAAAATAG	NA
SNP0442	Ca_03781	*Ca_Kabuli_Chr04*	[T/C]	4757357	CDS_SYNONYMOUS	SET domain	CAAACCTTGTGAAGAGTAAGCAAA	ATGGAGAAAGACCAAAACAGAAAC	NA
SNP0443	Ca_03753	*Ca_Kabuli_Chr04*	[G/A]	5018591	CDS_SYNONYMOUS	Basic-leucine zipper (bZIP) transcription factor	CGGCTTCCTTTCTTCTATCTTTCT	ACGAGCAAAGGTAAAGTCTTGATT	NA
SNP0444	Ca_03733	*Ca_Kabuli_Chr04*	[A/C]	5222669	CDS_SYNONYMOUS	Domain of unknown function DUF1981, SEC7 associated	TTCAGAGTTGGGATAAAAGCGTAT	ACAGATGACTGTATGAAGCCAAAA	NA
SNP0445	Ca_03702	*Ca_Kabuli_Chr04*	[T/G]	5528421	CDS_SYNONYMOUS	Zinc finger, RING-type	GGTAATCGAGACTTTTCCAACATT	GAAGGTGGAGACCATGTAAGCTAT	NA
SNP0446	Ca_03621	*Ca_Kabuli_Chr04*	[G/A]	6238354	CDS_SYNONYMOUS	High mobility group, HMG1/HMG2	ACTGTGGCCCTATAGTTGATTCAG	CGTTTTCAAGATAAGCCCAAATAC	NA
SNP0447	Ca_03591	*Ca_Kabuli_Chr04*	[G/A]	6507540	CDS_SYNONYMOUS	Helix-loop-helix DNA-binding domain	CAATGAATGTTTCTAGGGGTTCTT	GATACTCAATTGCTTCGTCTAGCA	NA
SNP0448	Ca_03510	*Ca_Kabuli_Chr04*	[T/G]	7239917	CDS_SYNONYMOUS	WD40 repeat	CCTCCTCTTCCTCTCTCTCTCTCT	CCTTTCCACCATAGTAGATGGTCT	NA
SNP0449	Ca_08313	*Ca_Kabuli_Chr04*	[C/A]	8236449	CDS_SYNONYMOUS	Zinc finger, RING-type	TCCTTCAGATAGCAAGACTTAGCA	AGTAACTGAAGCAATGTGGGAGTT	NA
SNP0450	Ca_08340	*Ca_Kabuli_Chr04*	[T/C]	8483482	CDS_SYNONYMOUS	WD40 repeat	AGGAGAGAGTTTTGAGGACAAATG	ATCTGGTTCTTTCTTCCCTCTTTT	NA
SNP0451	Ca_08341	*Ca_Kabuli_Chr04*	[C/A]	8496641	CDS_SYNONYMOUS	WD40 repeat	GCGAAGAAGCTGTATTGGAAATAG	AGGCTACTAACCAGAAGATCAAGC	NA
SNP0452	Ca_08371	*Ca_Kabuli_Chr04*	[T/C]	8751629	CDS_SYNONYMOUS	No apical meristem (NAM) protein	ACCTGAATGCCAAACCATCTATAC	GAAGAAAGCCCTTACTAACTGCAA	NA
SNP0453	Ca_08372	*Ca_Kabuli_Chr04*	[A/C]	8772215	CDS_SYNONYMOUS	No apical meristem (NAM) protein	CAAGATGGAGACTATGGTGAGCTA	TCCAGAACTTTATCTCACTTCACG	NA
SNP0454	Ca_08381	*Ca_Kabuli_Chr04*	[A/G]	8844846	CDS_SYNONYMOUS	Zinc finger, RING-type	CCTCTCATCTCTCTTCTTCCTTTG	CTGCGATTGTTATTTCTCTGACTG	NA
SNP0455	Ca_08385	*Ca_Kabuli_Chr04*	[G/T]	8873494	CDS_SYNONYMOUS	WD40 repeat	TAACAACCCTCAAGATACGAAATG	TATGGATGCAGAAATCATCACTTG	NA
SNP0456	Ca_05487	*Ca_Kabuli_Chr04*	[T/A]	16278671	CDS_SYNONYMOUS	Zinc finger, B-box	TTCACCGTAAGGATAAAGAACCTC	CACAAAGAAGGTGAATTGTGTGAT	NA
SNP0457	Ca_05477	*Ca_Kabuli_Chr04*	[T/C]	16501716	CDS_SYNONYMOUS	Domain of unknown function DUF292, eukaryotic	CAGGGGTAATTTTCGGTAAACATA	GGGTCACTCTTAAGGAAAAGGAGT	NA
SNP0458	Ca_05430	*Ca_Kabuli_Chr04*	[T/A]	16926527	CDS_SYNONYMOUS	Zinc finger, C2H2-type	CATGAGAAATTATATGGTCCATGC	AACAACCTCTTCAACATTCCTTTC	NA

SNP IDs	*Kabuli* /*desi* gene accession IDs	*Kabuli* /*desi* chromosomes	SNPs	Physical positions (bp)	Sequence components of genome	Putative functions	Forward primer sequences (5'-3')	Reverse primer sequences (5'-3')	Primers used in Figure 2A
SNP0459	Ca_05404	*Ca_Kabuli_Chr04*	[T/C]	17199316	CDS_SYNONYMOUS	Domain of unknown function DUF632	AAAAGGAGTGCTAGTCTGTTCGAT	TTTTCTTCTCAGTGTCTCCAAATG	NA
SNP0460	Ca_05403	*Ca_Kabuli_Chr04*	[A/G]	17202770	CDS_SYNONYMOUS	Protein of unknown function DUF3353	AGTGAAGGGTCAAAAACACCTTTA	ATGCTAACAACACACCCTCTCATA	NA
SNP0461	Ca_05384	*Ca_Kabuli_Chr04*	[A/T]	17429456	CDS_SYNONYMOUS	Transcription factor jumonji/aspartyl beta-hydroxylase	ATTTAAGACGCTTGGATGAAGAAC	GGACTTCAACCTTGAACTGGTATC	NA
SNP0462	Ca_05364	*Ca_Kabuli_Chr04*	[T/C]	17652735	CDS_SYNONYMOUS	Protein of unknown function DUF829, TMEM53	CACAGGTAAAGGCTTAGCAACTTT	CCAAAGAAATGAGTTAGTGGGATT	NA
SNP0463	Ca_18633	*Ca_Kabuli_Chr04*	[G/A]	17931955	CDS_SYNONYMOUS	Protein of unknown function DUF674	CCCAAATATTAGGCCATAGATGAG	TACAACTTTGAATTGGACCATACG	NA
SNP0464	Ca_17152	*Ca_Kabuli_Chr04*	[G/A]	20436136	CDS_SYNONYMOUS	Transcription factor, TCP	AGGGAACGTCATCATCATTCATTA	ATCGTGTCAACTTTTGTGTCTCAT	NA
SNP0465	Ca_14466	*Ca_Kabuli_Chr04*	[G/A]	22859422	CDS_SYNONYMOUS	Protein of unknown function DUF408	TCTATATTTCTCATGCATGCCACT	CCATTTCTCAAAATTTAGGTCCTC	NA
SNP0466	Ca_21317	*Ca_Kabuli_Chr04*	[G/T]	27493010	CDS_SYNONYMOUS	Protein of unknown function DUF716, TMEM45	CTTTTCATCCTCTCTCTACCTCCA	TTTCTATAATGCTCCTTCATGCAC	NA
SNP0467	Ca_21312	*Ca_Kabuli_Chr04*	[G/A]	27626559	CDS_SYNONYMOUS	Protein of unknown function DUF810	GTGAAACTGTTGTAGAGGAACCAA	CTGGTAGCATATCCCAGCTATACA	NA
SNP0468	Ca_14192	*Ca_Kabuli_Chr04*	[T/G]	30301002	CDS_SYNONYMOUS	SNF2-related	AATATTCTCCTCAATGGTGCTTTC	GTTTTCCAGTCTCACCAAGAATTT	NA
SNP0469	Ca_15459	*Ca_Kabuli_Chr04*	[T/G]	32042488	CDS_SYNONYMOUS	Domain of unknown function DUF828	TTTACAAGGTTAGCCACAACTTCA	GCTGCAATGTTCCATACTTCTTTA	NA
SNP0470	Ca_14853	*Ca_Kabuli_Chr04*	[T/C]	36154771	CDS_SYNONYMOUS	Protein of unknown function DUF827, plant	ATGACAGAACTTGATGCTGCTAAG	CACCTTCTGCACAAACTATACAGG	NA
SNP0471	Ca_14840	*Ca_Kabuli_Chr04*	[T/C]	36368713	CDS_SYNONYMOUS	Domain of unknown function DUF1084	ATACATGTTGCAATAGGGGTTTCT	AAATATGTCAGGAATTCCACCACT	NA
SNP0472	Ca_14825	*Ca_Kabuli_Chr04*	[C/T]	36537118	CDS_SYNONYMOUS	Transcriptional factor B3	AACCATCTAATCCTTTCTGTCGAG	ATTTCCGACACTAACACAGATTCA	NA
SNP0473	Ca_15144	*Ca_Kabuli_Chr04*	[T/A]	37316574	CDS_SYNONYMOUS	Zinc finger, RING-type	GGTTGAGTGATTTTGATTTGCCTA	CGACGATGTAAAGAGATTTTGTATTG	NA
SNP0474	Ca_13141	*Ca_Kabuli_Chr04*	[T/C]	38039371	CDS_SYNONYMOUS	Protein of unknown function DUF3326	GGCTGATAATATAGCATGGGTTTC	CAACAACTACAAACGAACCATTTC	NA
SNP0475	Ca_13128	*Ca_Kabuli_Chr04*	[G/T]	38288823	CDS_SYNONYMOUS	Protein of unknown function DUF869, plant	CCCCCTAAGTTTCTCCTCATAAAT	CCTCACTCAACTGAAGTTGTGACT	NA
SNP0476	Ca_13102	*Ca_Kabuli_Chr04*	[T/C]	38659377	CDS_SYNONYMOUS	Transcription factor GRAS	ATGCCAAGCTATACTCGTTAAAGG	AACATTCACCCTATCCTGACTCTC	NA
SNP0477	Ca_14925	*Ca_Kabuli_Chr04*	[T/C]	39803305	CDS_SYNONYMOUS	SANT domain, DNA binding	TGCAAAGAGATGGTACAGATCCTA	GGACACTCTGGTTCAAAGCTATTT	NA
SNP0478	Ca_14868	*Ca_Kabuli_Chr04*	[G/A]	40583219	CDS_SYNONYMOUS	Zinc finger, RING-type	TCACAGTGACAACTGCAATACATC	TTGATGACCAAATTGTTAGGAGTC	NA
SNP0479	Ca_10978	*Ca_Kabuli_Chr04*	[T/C]	41319598	CDS_SYNONYMOUS	Zinc finger, U1-type	TCATTCTGATGGGAAGGCTATAAT	GGCTAAACTTTGCATATGATTTCC	NA
SNP0480	Ca_10932	*Ca_Kabuli_Chr04*	[G/A]	41864826	CDS_SYNONYMOUS	WD40 repeat	AACTCAACCTTACAAAACCAGCTT	ATATTTATTGCCAGGACAAACGAG	NA
SNP0481	Ca_09060	*Ca_Kabuli_Chr04*	[C/T]	43807399	CDS_SYNONYMOUS	WD40 repeat	TAATGATCTCCAGTCTTCGCAGTA	AAATCATCAAGGCTAACACACTCA	NA
SNP0482	Ca_09097	*Ca_Kabuli_Chr04*	[A/C]	44239783	CDS_SYNONYMOUS	BTB/POZ-like	TCTATTTTAGGCAATAGGGCGATA	TATGAGATTAGCCGTGATTCTTTG	NA
SNP0483	Ca_09101	*Ca_Kabuli_Chr04*	[C/T]	44266758	CDS_SYNONYMOUS	DNA-binding WRKY	CTGGTTGGATGAAGAAAACAAAC	GATTTCAAGAGAGATTGCAAGGTT	NA
SNP0484	Ca_09120	*Ca_Kabuli_Chr04*	[G/A]	44472282	CDS_SYNONYMOUS	Protein of unknown function DUF1350	CTATGTTTGATGGTTTGAGACACC	AATATAGGTGTCAGTGTCGTGTCG	NA
SNP0485	Ca_09153	*Ca_Kabuli_Chr04*	[A/G]	44906255	CDS_SYNONYMOUS	Protein of unknown function DUF803	AGATACTGCTTAATGGATGGCTTC	GGTATCAATGTGACATGGTAAGGA	NA

SNP IDs	*Kabuli* /*desi* gene accession IDs	*Kabuli* /*desi* chromosomes	SNPs	Physical positions (bp)	Sequence components of genome	Putative functions	Forward primer sequences (5'-3')	Reverse primer sequences (5'-3')	Primers used in Figure 2A
SNP0486	Ca_18384	*Ca_Kabuli_Chr04*	[A/T]	45782453	CDS_SYNONYMOUS	WD40 repeat	ACTGCTCAATAGCTTTGGGTACAT	TACTACCAACCAAGAGGAGTCACA	NA
SNP0487	Ca_19258	*Ca_Kabuli_Chr04*	[T/A]	46605131	CDS_SYNONYMOUS	Protein of unknown function DUF827, plant	CTTTGGAGGATGAAAGATGGTAAT	TTAAGCACGCAGTTTCTAAGTTTG	NA
SNP0488	Ca_19256	*Ca_Kabuli_Chr04*	[G/A]	46612175	CDS_SYNONYMOUS	Protein of unknown function DUF827, plant	CTTCTTTTCTCACCAAGAGTGATG	ACATATGGAAGCAGAAGAACACAG	NA
SNP0489	Ca_10847	*Ca_Kabuli_Chr04*	[A/G]	47419514	CDS_SYNONYMOUS	Protein of unknown function DUF616	TAACTGGCAAGGCCAATCTTATAG	CGTAATCTAGCAATTCTGGGTTTT	NA
SNP0490	Ca_10812	*Ca_Kabuli_Chr04*	[T/C]	47819943	CDS_SYNONYMOUS	Protein of unknown function DUF248, methyltransferase putative	AAACATCCTTATCAAGGAGAGCAG	GGAAGATGGAAACATTGAAGAGTT	NA
SNP0491	Ca_10755	*Ca_Kabuli_Chr04*	[C/T]	48322502	CDS_SYNONYMOUS	Basic-leucine zipper (bZIP) transcription factor	TACTATATGGATGCGACAATAGCC	ACTTTGGAGTGTTTGTGTTTGTGT	NA
SNP0492	Ca_10748	*Ca_Kabuli_Chr04*	[G/C]	48382666	CDS_SYNONYMOUS	AUX/IAA protein	GGATCATCACCAACAAGTAGAACA	CGAATCTCTACCAAGAACAAATGA	NA
SNP0493	Ca_10746	*Ca_Kabuli_Chr04*	[G/C]	48464434	CDS_SYNONYMOUS	Zinc finger, CCHC-type	AAATAAGACATGGTGTTGGAGTCA	CAGCAGACAAATACCCAATAACAG	NA
SNP0494	Ca_23018	*Ca_Kabuli_Chr04*	[T/C]	48678278	CDS_SYNONYMOUS	Transcription factor IIS, N-terminal	CCTTGCAGTCAACACATCTCTATT	CGGATTCTGTAAGGAAAAATGAAC	NA
SNP0495	Ca_18171	*Ca_Kabuli_Chr05*	[T/G]	166216	CDS_SYNONYMOUS	No apical meristem (NAM) protein	AGGAGCTGATAGGATGATCAGAAC	TGAGGAATAATGAGAGTGACAAGC	NA
SNP0496	Ca_18177	*Ca_Kabuli_Chr05*	[C/G]	223384	CDS_SYNONYMOUS	ZF-HD homeobox protein, Cys/His-rich dimerisation domain	CTATTTGTTGACACAACACCACAA	ACCCTCTTCTTGTTCATCAGATTC	NA
SNP0497	Ca_26279	*Ca_Kabuli_Chr05*	[A/C]	1281006	CDS_SYNONYMOUS	Transcription factor GRAS	AAATCTGATTCCGAAGAACAAGTC	CTATGGAAATGGACATGGATACAA	NA
SNP0498	Ca_20508	*Ca_Kabuli_Chr05*	[T/C]	9961273	CDS_SYNONYMOUS	DNA-binding WRKY	GGAATCATATGCAACAGAGATGAG	GATTAATGTGTGTGAAGTCCACCT	NA
SNP0499	Ca_21977	*Ca_Kabuli_Chr05*	[A/T]	23161492	CDS_SYNONYMOUS	Zinc finger, PHD-type	GGATGATGCATAACAGAATCCTTA	ATTTACCCCTAACCAACCCTTTTA	NA
SNP0500	Ca_17665	*Ca_Kabuli_Chr05*	[G/T]	23934198	CDS_SYNONYMOUS	WD40 repeat	AAAAGTGCTGGCAGAAAACTACAT	GAGTTGGTTGCTTAAACATGACAG	NA
SNP0501	Ca_09042	*Ca_Kabuli_Chr05*	[T/G]	25399115	CDS_SYNONYMOUS	Zinc finger, CCCH-type	CAAATATAAAACGCTCCCAAAGTC	AATCATGAGAATAAGCCCTTGAAC	NA
SNP0502	Ca_08907	*Ca_Kabuli_Chr05*	[C/T]	27361515	CDS_SYNONYMOUS	BTB/POZ-like	ATTTGCAACTCCAATTCTATAGGC	AGAGTCATCACAGGTATGAAGCAG	NA
SNP0503	Ca_08872	*Ca_Kabuli_Chr05*	[C/T]	27750155	CDS_SYNONYMOUS	AUX/IAA protein	GAATATTGTGCATGTGACATTGTG	TGTATGGCACACTAGAAGGGTTTA	NA
SNP0504	Ca_13388	*Ca_Kabuli_Chr05*	[C/T]	28346497	CDS_SYNONYMOUS	Lateral organ boundaries, LOB	TGACACATTAGGTAACGGGTGAAT	ACATAGTTTTTCTCAATGCGAGAG	NA
SNP0505	Ca_13361	*Ca_Kabuli_Chr05*	[A/C]	28682973	CDS_SYNONYMOUS	BTB/POZ-like	AGGATTCAACATAGAGGAGTTTCT	TACCTGCACATAAATCTGTTTTGG	NA
SNP0506	Ca_16670	*Ca_Kabuli_Chr05*	[T/A]	29886086	CDS_SYNONYMOUS	Bromodomain	CTAAGAGGCAAGTCTCTCCTGAAA	CACACGAAATAGACTGTCAAAAGG	NA
SNP0507	Ca_04764	*Ca_Kabuli_Chr05*	[G/A]	30697511	CDS_SYNONYMOUS	Zinc finger, RING-type	TCAGCCTCTACTTTTGTTTTGTTG	AGATCAATCCATTTCAAGGTCTGT	NA
SNP0508	Ca_04906	*Ca_Kabuli_Chr05*	[G/A]	32019864	CDS_SYNONYMOUS	Protein of unknown function DUF726	GTGACATCTGTTTGCTTGAAATTG	CCTGTCATAGCATTCAAACACCTA	NA
SNP0509	Ca_04996	*Ca_Kabuli_Chr05*	[A/T]	32978975	CDS_SYNONYMOUS	Domain of unknown function DUF676, hydrolase-like	CAGAAACAGTTGCGATAGATGAAA	ACAAAGGTTCAGATCAAATGACAC	NA
SNP0510	Ca_01919	*Ca_Kabuli_Chr05*	[A/G]	33379904	CDS_SYNONYMOUS	Homeobox	TGCATAAGCAGGAGAACATAATTC	GGAGGTCAGGTTATTCTTCCACTA	NA
SNP0511	Ca_01913	*Ca_Kabuli_Chr05*	[T/C]	33431897	CDS_SYNONYMOUS	Protein of unknown function DUF810	ATTGAAGGCATCTATCGAGGTTAT	AGGTCATCTCTACGTACTGCCTTT	NA
SNP0512	Ca_01882	*Ca_Kabuli_Chr05*	[A/G]	33721544	CDS_SYNONYMOUS	Protein of unknown function DUF81	ATGAGAAGTGCCAAATCATAGTCA	CTGTGAATTTCTTATGGCAAACAG	NA

SNP IDs	*Kabuli* /*desi* gene accession IDs	*Kabuli* /*desi* chromosomes	SNPs	Physical positions (bp)	Sequence components of genome	Putative functions	Forward primer sequences (5'-3')	Reverse primer sequences (5'-3')	Primers used in Figure 2A
SNP0513	Ca_01824	*Ca_Kabuli_Chr05*	[C/G]	34241269	CDS_SYNONYMOUS	WD40 repeat	TAAAACTGCAGCTTCAGAGGTATG	CATAGTGATATGCCCATTAGATGC	NA
SNP0514	Ca_01790	*Ca_Kabuli_Chr05*	[T/C]	34511678	CDS_SYNONYMOUS	WD40 repeat	CAAGTAACAAAGCCACGGACTAAT	GTATGGTTGATGACAACATGGAAT	NA
SNP0515	Ca_01595	*Ca_Kabuli_Chr05*	[C/G]	36292611	CDS_SYNONYMOUS	WD40 repeat	AAGGGTAAAAGTGTAAAAGCATGG	GACCAGACAAAGTATGAGCAAATG	NA
SNP0516	Ca_01585	*Ca_Kabuli_Chr05*	[A/C]	36397489	CDS_SYNONYMOUS	Transcription initiation factor TFIID component TAF4	TGAACAACCTAGTAGCCAAATCAA	CGATGCATATACTATAACGCCAAC	NA
SNP0517	Ca_01576	*Ca_Kabuli_Chr05*	[C/T]	36441063	CDS_SYNONYMOUS	Protein of unknown function DUF620	CTTCCTCAATTATCCATGCTTCTT	TGTAGCTTCTCTCTTTCCTCCTCT	NA
SNP0518	Ca_01540	*Ca_Kabuli_Chr05*	[G/T]	36800497	CDS_SYNONYMOUS	Protein of unknown function DUF827, plant	GGTCCACTTCATTTTTGCTTCTAT	GCATGTTAAAGCTCTTTTGGAGAT	NA
SNP0519	Ca_01510	*Ca_Kabuli_Chr05*	[G/A]	37040045	CDS_SYNONYMOUS	Protein of unknown function DUF2053, membrane	AGCTTTCTCACAGTGAAGGTCTCT	TTCACCTAAAAGCTTGTGCTTATG	NA
SNP0520	Ca_01503	*Ca_Kabuli_Chr05*	[C/T]	37147572	CDS_SYNONYMOUS	ZF-HD homeobox protein, Cys/His-rich dimerisation domain	GAAGCAAAATAGCTTAACCCAAAC	ATATTAGAGCGTAGAAGCGGTGAT	NA
SNP0521	Ca_01389	*Ca_Kabuli_Chr05*	[A/C]	38347201	CDS_SYNONYMOUS	WD40 repeat	GGTAACTTTTCGGAAATTGGAAG	TCTGACGTGGGTTCTGACTTAATA	NA
SNP0522	Ca_07497	*Ca_Kabuli_Chr05*	[G/A]	39713021	CDS_SYNONYMOUS	Protein of unknown function DUF3506	AAAAAGATAAGCCAACTCACCATC	ACAGGTCATGGTCTGAATGTAAAA	NA
SNP0523	Ca_07571	*Ca_Kabuli_Chr05*	[A/G]	40507107	CDS_SYNONYMOUS	Zinc finger, CCHC-type	TCACATCCAATGTAAGCCATATTC	TAAATTTTGAGGGGTGTGAATAGC	NA
SNP0524	Ca_11315	*Ca_Kabuli_Chr05*	[T/G]	42928986	CDS_SYNONYMOUS	SANT domain, DNA binding	AAAGATACACTTTGTGCCTGATTG	GGGAATAGGATCATTTAGTGCGTA	NA
SNP0525	Ca_12651	*Ca_Kabuli_Chr05*	[G/A]	44064495	CDS_SYNONYMOUS	Domain of unknown function DUF632	ATTATCCAGTTCCACTTTCTTTGG	ATGCAAGTTCTTAGTTCAGGTGGT	NA
SNP0526	Ca_03899	*Ca_Kabuli_Chr05*	[T/C]	44561095	CDS_SYNONYMOUS	Protein of unknown function DUF248, methyltransferase putative	TCACCAGGATAAGGATTAAGGAAA	GAAGAGAAGTCGTGTGATATTGGA	NA
SNP0527	Ca_03920	*Ca_Kabuli_Chr05*	[A/G]	44760403	CDS_SYNONYMOUS	Transcription factor GRAS	AGTTCAGGAGCTTCACTGCTTACT	AGACTATCTTGTTCACCTCCAAGC	NA
SNP0528	Ca_03969	*Ca_Kabuli_Chr05*	[C/T]	45254102	CDS_SYNONYMOUS	Zinc finger, C2H2-type matrin	TTAAGGCTCGTTTGATTCTCATTC	ACAACAAGCACTGGTGTTTTCAT	NA
SNP0529	Ca_04012	*Ca_Kabuli_Chr05*	[T/C]	45661109	CDS_SYNONYMOUS	Protein of unknown function DUF791	GTAACAACGTTGGAGTAGCAAATG	AATCGGGCTTAACATGTCTGTAAT	NA
SNP0530	Ca_04097	*Ca_Kabuli_Chr05*	[G/T]	46403922	CDS_SYNONYMOUS	Protein of unknown function DUF3133	GGTGTTCTTGGAATAGGAAAACTG	AGACATATTGGAGGAAAATTCAGC	NA
SNP0531	Ca_04110	*Ca_Kabuli_Chr05*	[A/C]	46526963	CDS_SYNONYMOUS	Protein of unknown function DUF2044, membrane	ATGTAGAAAAGGGCTGTTACTTGG	CACAATTAGAAGCTGGAACTCTCA	NA
SNP0532	Ca_04172	*Ca_Kabuli_Chr05*	[C/G]	47151660	CDS_SYNONYMOUS	WD40 repeat	TCTCCGATGAAAGAGATAATGATG	GAATTGAATGCAAGTCACTGTTTC	NA
SNP0533	Ca_04239	*Ca_Kabuli_Chr05*	[C/T]	47775175	CDS_SYNONYMOUS	Protein of unknown function DUF869, plant	CTCTACTATCTCCAACTCCGCTTC	AGCTAAAATGCTATGGCTTCAAAC	NA
SNP0534	Ca_13320	*Ca_Kabuli_Chr06*	[C/T]	1130293	CDS_SYNONYMOUS	Domain of unknown function DUF296	CCACCAACAAAGTTTATCTCATCA	CGATTCAGCTGCAGAGTTAGATAG	NA
SNP0535	Ca_10329	*Ca_Kabuli_Chr06*	[T/A]	2078416	CDS_SYNONYMOUS	Protein of unknown function DUF760	CTAAAATAGGGTGACAGCGGTAGT	CCACTGATCTTGTTTTGTACAGGT	NA
SNP0536	Ca_10387	*Ca_Kabuli_Chr06*	[A/G]	2548412	CDS_SYNONYMOUS	Zinc finger, RING-type	CAATAGTTGCACCATTAAACCATC	TGGTTCCTAGGGAAACAGACTTAG	NA
SNP0537	Ca_10405	*Ca_Kabuli_Chr06*	[T/C]	2714894	CDS_SYNONYMOUS	Domain of unknown function DUF125, transmembrane	TAATCAAGTCGTTGTCACTGTCCT	TGGAGTCTATAATCACGTCCACAC	NA
SNP0538	Ca_10423	*Ca_Kabuli_Chr06*	[G/A]	2864713	CDS_SYNONYMOUS	SET domain	AATCACGTTAGGGGTTTTCAACTA	TTGTGATCTTATGGTTCTTGTGCT	NA
SNP0539	Ca_10431	*Ca_Kabuli_Chr06*	[T/C]	2926006	CDS_SYNONYMOUS	Tify	TTCTCTCTTCTGTTCACGTTTTTG	GTAAGTGAAAAATGAAAGCCACCT	NA

SNP IDs	*Kabuli* /*desi* gene accession IDs	*Kabuli* /*desi* chromosomes	SNPs	Physical positions (bp)	Sequence components of genome	Putative functions	Forward primer sequences (5'-3')	Reverse primer sequences (5'-3')	Primers used in Figure 2A
SNP0540	Ca_05731	*Ca_Kabuli_Chr06*	[C/A]	5628948	CDS_SYNONYMOUS	Nin one binding (NOB1) Zn-ribbon-like	AGCTCTTAATGAAGAAAACGCACT	ACACCGAACTAGTAGGCCAATAAA	NA
SNP0541	Ca_16913	*Ca_Kabuli_Chr06*	[A/G]	6444169	CDS_SYNONYMOUS	SANT domain, DNA binding	GCAGGTTGTTGAATAGTAGCAGAA	GGGCAGAGATGTTGAGAATTTATTA	NA
SNP0542	Ca_08667	*Ca_Kabuli_Chr06*	[A/G]	9323775	CDS_SYNONYMOUS	Homeobox	CTAGTGACGTGTTTTCTCCTCAGA	TTCTGCAGGTAAAGTCATTTGTGT	NA
SNP0543	Ca_08608	*Ca_Kabuli_Chr06*	[A/T]	9834184	CDS_SYNONYMOUS	WD40 repeat	TATGGTGAAACAGAGTTTGAAGGA	CCAGGTCTGTGCATAAAACTACAA	NA
SNP0544	Ca_08563	*Ca_Kabuli_Chr06*	[C/T]	10260274	CDS_SYNONYMOUS	Mediator complex, subunit Med10	GATCTCTCAAACAAACGACACAAC	AACGTAAAAGTTATGGGCTAGGTG	NA
SNP0545	Ca_20467	*Ca_Kabuli_Chr06*	[A/C]	10991737	CDS_SYNONYMOUS	Zinc finger, MYND-type	CCCTTTTGCCTTAGTTTTTGTATC	ACCGTTAACCATAGCCATAATACC	NA
SNP0546	Ca_05025	*Ca_Kabuli_Chr06*	[A/G]	12437285	CDS_SYNONYMOUS	AUX/IAA protein	ACTCACAACATTTCCAATGTATCC	TACAATGTAGCAAGGGAGTGAAAG	NA
SNP0547	Ca_05057	*Ca_Kabuli_Chr06*	[G/A]	12844524	CDS_SYNONYMOUS	Protein of unknown function DUF593	CAGGAACAAGTGGATGAGTATGAA	ACCTCCTCTTCTAAACCAACCTTT	NA
SNP0548	Ca_05097	*Ca_Kabuli_Chr06*	[A/G]	13243282	CDS_SYNONYMOUS	Zinc finger, CCCH-type	ATCCACTGTTAATGGCTTGTTTTC	TGTCAGATCTCAGACACCGTTTAT	NA
SNP0549	Ca_05213	*Ca_Kabuli_Chr06*	[A/G]	14416981	CDS_SYNONYMOUS	Domain of unknown function DUF676, hydrolase-like	TCATATTGTCCTGTCCACTTTGAT	GTGCTATACCTCGCCTATGAAGTT	NA
SNP0550	Ca_05273	*Ca_Kabuli_Chr06*	[T/C]	14982998	CDS_SYNONYMOUS	BTB/POZ-like	AATGCAGATATAGTAGCGCCTTTT	AATGATTGTTGTCTGCTTGGACTA	NA
SNP0551	Ca_05286	*Ca_Kabuli_Chr06*	[T/C]	15051094	CDS_SYNONYMOUS	Protein of unknown function DUF3506	TTTGATTTCTCAGGAATAGTCACG	AAAAACAGATGCTACAGACAGTGC	NA
SNP0552	Ca_05300	*Ca_Kabuli_Chr06*	[T/C]	15185723	CDS_SYNONYMOUS	Protein of unknown function DUF593	AACCTAAATCTTGAGGCTAAGCAA	CTCTCTCTCTCTCTAACCCAATGC	NA
SNP0553	Ca_05318	*Ca_Kabuli_Chr06*	[A/C]	15328948	CDS_SYNONYMOUS	Zinc finger, DHHC-type, palmitoyltransferase	GTTTGTTGTAATGATTGAGGTTCC	TACCCGGTATCCATTTATATCAGC	NA
SNP0554	Ca_06343	*Ca_Kabuli_Chr06*	[C/T]	17262283	CDS_SYNONYMOUS	Homeobox	TTTACCTCTTCGGAATCAATAAGC	GTAGCAAAAATGCAAGAGAGAGGT	NA
SNP0555	Ca_06352	*Ca_Kabuli_Chr06*	[A/G]	17369613	CDS_SYNONYMOUS	Transcriptional factor B3	TGTAGGTCCAGTCCTTGTTACTCA	GTGTATTGTCAGGTGTTGTTGGTT	NA
SNP0556	Ca_06536	*Ca_Kabuli_Chr06*	[A/G]	19304834	CDS_SYNONYMOUS	Zinc finger, ZPR1-type	ATCTCGATACTTGCCTTCTTTTTG	GCATTATGTTTGTCCAGTCCATAG	NA
SNP0557	Ca_16355	*Ca_Kabuli_Chr06*	[T/A]	20766342	CDS_SYNONYMOUS	Helix-loop-helix DNA-binding domain	TACTTGGCACTTGCTATTTGAATC	GACACAATTCCTCTTTTCCTTGAT	NA
SNP0558	Ca_11272	*Ca_Kabuli_Chr06*	[G/T]	21675762	CDS_SYNONYMOUS	Homeobox	CTCTTCTTCTTCGTTTGTTTTTGG	AGCAATTCTCTCTGACTTCACTTG	NA
SNP0559	Ca_11216	*Ca_Kabuli_Chr06*	[G/A]	22217107	CDS_SYNONYMOUS	Zinc finger, CHCC-type	AAATGTGAGATTGGAGAAAGTTCC	ATAGTGGAAGAATTCTGCATTGAG	NA
SNP0560	Ca_11200	*Ca_Kabuli_Chr06*	[C/T]	22370242	CDS_SYNONYMOUS	Protein of unknown function DUF1218	ACTTGGTGCTCAGTATCAGATTCA	CAAACCAAATCAAACTAACACG	NA
SNP0561	Ca_16676	*Ca_Kabuli_Chr06*	[C/T]	26349321	CDS_SYNONYMOUS	Transcription factor GRAS	GAACTTAACAATTCTGTGCGTTTG	ACTTCTAGGACTGTTCGCTTCAAT	NA
SNP0562	Ca_16677	*Ca_Kabuli_Chr06*	[C/T]	26353512	CDS_SYNONYMOUS	Transcription factor GRAS	GAAATCCGTGTAAAGGGAACTCTA	AACAAACAGACAAACATACGATGG	NA
SNP0563	Ca_16678	*Ca_Kabuli_Chr06*	[C/G]	26357149	CDS_SYNONYMOUS	Transcription factor GRAS	AACTCTTTCTCGAACATCAACCTC	GCCTTCTATATTGCAAACCACTTT	NA
SNP0564	Ca_16690	*Ca_Kabuli_Chr06*	[A/G]	26567360	CDS_SYNONYMOUS	Zinc finger, Dof-type	TCTGTTTCGTTTCTGTCAAAAGAG	GAATAAGCCCAAAGTAAACCAAAC	NA
SNP0565	Ca_14604	*Ca_Kabuli_Chr06*	[A/G]	27537433	CDS_SYNONYMOUS	Zinc finger, RING-type	CCCTGTGTATAATTGAAACCGATA	CAATGTGACACTGTTATGGAACAA	NA
SNP0566	Ca_24048	*Ca_Kabuli_Chr06*	[T/G]	30127756	CDS_SYNONYMOUS	Domain of unknown function DUF296	CTCATCATCCCCAATTTCATCTAT	GTGGTCAAATTTCCGTACAAATATC	NA

SNP IDs	*Kabuli* /*desi* gene accession IDs	*Kabuli* /*desi* chromosomes	SNPs	Physical positions (bp)	Sequence components of genome	Putative functions	Forward primer sequences (5'-3')	Reverse primer sequences (5'-3')	Primers used in Figure 2A
SNP0567	Ca_16493	*Ca_Kabuli_Chr06*	[A/T]	31024728	CDS_SYNONYMOUS	Zinc finger, Sec23/Sec24-type	GAGGATTACAAATCCATCCCATAC	CTTCAGCCACAAACTAGGACTGTA	NA
SNP0568	Ca_22063	*Ca_Kabuli_Chr06*	[A/G]	33733259	CDS_SYNONYMOUS	Zinc finger, RING-type	TGTCTTCACAGAGTTTGGATCTTC	GTTGCAACTACTACAGCTGGACAT	NA
SNP0569	Ca_15788	*Ca_Kabuli_Chr06*	[G/A]	35129588	CDS_SYNONYMOUS	Zinc finger, PHD-type	TAGAATCTGGTTTGACTGTTTCCA	GACATTCCTTTTTGTGTGTGTTGT	NA
SNP0570	Ca_15898	*Ca_Kabuli_Chr06*	[C/T]	38816345	CDS_SYNONYMOUS	Transcription factor GRAS	AGCCCTAGTCCTCCTATTTCATCT	AAGAAATGACCAGGTAGAGGACAC	NA
SNP0571	Ca_13854	*Ca_Kabuli_Chr06*	[T/C]	45709318	CDS_SYNONYMOUS	DNA-binding WRKY	TTGGGTCTAAGTCAACCCTAAAAC	AAGGGTTCTTGTTGAAGCAACTAT	NA
SNP0572	Ca_13499	*Ca_Kabuli_Chr06*	[C/G]	49664132	CDS_SYNONYMOUS	Domain of unknown function DUF632	CCTTTACAGCATCTAGTTGTCCAC	ACACATTAAATTGAAGGCGTGTC	NA
SNP0573	Ca_17511	*Ca_Kabuli_Chr06*	[A/G]	52059750	CDS_SYNONYMOUS	Auxin responsive SAUR protein	TTATGAAGGGAAGGTACCTCATGT	GGAAGTTAAATGGGATTACATGCT	NA
SNP0574	Ca_21219	*Ca_Kabuli_Chr06*	[G/A]	55332515	CDS_SYNONYMOUS	Homeobox	ATAGGTCAGGTTTTCTTGATCAGC	CTTGAAACTACCATAATGGGCAAT	NA
SNP0575	Ca_13700	*Ca_Kabuli_Chr06*	[A/G]	57437904	CDS_SYNONYMOUS	Bromodomain	TGGCAATTCTTCTAGCATAGTCAG	GTTTTGATGATATAGGTGGGCTCT	NA
SNP0576	Ca_13682	*Ca_Kabuli_Chr06*	[C/T]	57632486	CDS_SYNONYMOUS	Protein of unknown function DUF936, plant	TGATTTTATGGTGTGTCCTTGAAC	GGACTTCACTCCCTTCATCTATTG	NA
SNP0577	Ca_13671	*Ca_Kabuli_Chr06*	[T/C]	57720446	CDS_SYNONYMOUS	Transcriptional factor B3	CAGTGTATGGCGTGTAGGATTAAA	ACCGACAACATGCTAAAAACTATG	NA
SNP0578	Ca_15410	*Ca_Kabuli_Chr06*	[A/C]	58650318	CDS_SYNONYMOUS	WD40 repeat	TAGGTTGCTCCACAGACTACAAAC	AGCAATTGATGAGAAAGATCACTG	NA
SNP0579	Ca_03393	*Ca_Kabuli_Chr07*	[A/G]	845488	CDS_SYNONYMOUS	Domain of unknown function DUF221	AGATATCCCATAACAATTGGCACT	ACACGTTGTGGTATATTGCATCTT	NA
SNP0580	Ca_03388	*Ca_Kabuli_Chr07*	[G/A]	888900	CDS_SYNONYMOUS	DNA-binding WRKY	ATGAAGAGAACGAAAATCTGAAGG	CATCTGCAAGTAAGAGCAGTGTTT	NA
SNP0581	Ca_03275	*Ca_Kabuli_Chr07*	[G/T]	1991642	CDS_SYNONYMOUS	Zinc finger, B-box	ATAAGGAGAGATGAAGGGACAGTG	AAACACGATGACAACTACAACTCC	NA
SNP0582	Ca_03202	*Ca_Kabuli_Chr07*	[C/T]	2757260	CDS_SYNONYMOUS	Transcription factor jumonji/aspartyl beta-hydroxylase	ACAACTTTGCAAGAGTAACATTGG	CACCTACTGTGAAAACTTTGATGC	NA
SNP0583	Ca_03193	*Ca_Kabuli_Chr07*	[C/T]	2832369	CDS_SYNONYMOUS	Zinc finger, GATA-type	ACGAAAGTCCACTTCAAAATTAGG	GAGAGTGCGGATACAAAGATGATA	NA
SNP0584	Ca_03139	*Ca_Kabuli_Chr07*	[G/A]	3297560	CDS_SYNONYMOUS	Domain of unknown function DUF231, plant	AGCTCCAACTTTCCCACCTATATT	TATCAAAGTACCCTTTGGCTTCTC	NA
SNP0585	Ca_03128	*Ca_Kabuli_Chr07*	[A/G]	3355763	CDS_SYNONYMOUS	AUX/IAA protein	AGTCATAACCCCTTGAAAGCATAG	CATCCATGCTCTCATCAAATACTC	NA
SNP0586	Ca_03115	*Ca_Kabuli_Chr07*	[T/C]	3536233	CDS_SYNONYMOUS	Zinc finger, FYVE-type	TGTCTCACCTGCTACTTCATCTTC	GCCTTCATATAGTTCTTGGTCACA	NA
SNP0587	Ca_03114	*Ca_Kabuli_Chr07*	[A/G]	3543765	CDS_SYNONYMOUS	Zinc finger, FYVE-type	ATGTGTGAGCATGTTTGAGCTATT	TTGGTATTGTAGGGAGAAGGGTAA	NA
SNP0588	Ca_03113	*Ca_Kabuli_Chr07*	[C/T]	3560250	CDS_SYNONYMOUS	Zinc finger, FYVE-type	CATTCTGTTTATCTTCCTCCACCT	GGCTAGTGTTTTCCAAAAGAAAGA	NA
SNP0589	Ca_06816	*Ca_Kabuli_Chr07*	[A/C]	5508162	CDS_SYNONYMOUS	SNF2-related	GAAACAGTTCTACGAAGCAATGAA	GAAGGGATAGCTGAGAATTCAAAA	NA
SNP0590	Ca_06681	*Ca_Kabuli_Chr07*	[G/T]	6815151	CDS_SYNONYMOUS	YABBY protein	ACTGCAGCAATCTATGGTCTGTTA	GCCCTTAAAACTCATCAAAAGTGA	NA
SNP0591	Ca_06642	*Ca_Kabuli_Chr07*	[A/G]	7147557	CDS_SYNONYMOUS	Protein of unknown function DUF1644	GTTGAATTGCCGATGTATTACAAG	CCCAGAGTCTAGATGGTCTCCTAA	NA
SNP0592	Ca_06627	*Ca_Kabuli_Chr07*	[T/G]	7304614	CDS_SYNONYMOUS	Myb, DNA-binding	TGAAAGGGCAAATATGTCTAACTG	GGAGTCCAGTAGCATCATCTCATA	NA
SNP0593	Ca_13149	*Ca_Kabuli_Chr07*	[C/T]	8535189	CDS_SYNONYMOUS	ZF-HD homeobox protein, Cys/His-rich dimerisation domain	CGCTTTGTACGTATCCTCTCTTTC	CATGCTAAGAACTTTGGTCATTCA	NA

SNP IDs	*Kabuli* /*desi* gene accession IDs	*Kabuli* /*desi* chromosomes	SNPs	Physical positions (bp)	Sequence components of genome	Putative functions	Forward primer sequences (5'-3')	Reverse primer sequences (5'-3')	Primers used in Figure 2A
SNP0594	Ca_15363	*Ca_Kabuli_Chr07*	[C/T]	8553114	CDS_SYNONYMOUS	ZF-HD homeobox protein, Cys/His-rich dimerisation domain	TGAAGAACACAACACTATCTGACATC	GACAATGGAAGAGTTGTATGGCTA	NA
SNP0595	Ca_15359	*Ca_Kabuli_Chr07*	[T/C]	8583890	CDS_SYNONYMOUS	Zinc finger, C2HC5-type	AATCGGGATATACGAGTCACATTT	AACCTTGCGGTTGTGTAAGTTAAT	NA
SNP0596	Ca_15325	*Ca_Kabuli_Chr07*	[T/C]	8895087	CDS_SYNONYMOUS	Domain of unknown function DUF629	ATTTTCATGCCAGTGTGATGTTAG	TTGATTTCAGGACCTTGACTGATA	NA
SNP0597	Ca_17298	*Ca_Kabuli_Chr07*	[C/G]	9555319	CDS_SYNONYMOUS	Protein of unknown function DUF1645	CACAAAAGATAAGCCACCCATATT	CCTTTTTATGTCACCCAAAACTCT	NA
SNP0598	Ca_12811	*Ca_Kabuli_Chr07*	[G/T]	10521423	CDS_SYNONYMOUS	Zinc finger, RING-type	AGGGATAGAGAAGGAACAGAAACA	TATATTTAACCCATCCGCTTCTTC	NA
SNP0599	Ca_12840	*Ca_Kabuli_Chr07*	[G/T]	10859842	CDS_SYNONYMOUS	Zinc finger, RING-type	CAATATAGATTGGTGGCAACATGA	CAACCCTTGATGATCCTAGCTTAT	NA
SNP0600	Ca_09371	*Ca_Kabuli_Chr07*	[G/A]	11192667	CDS_SYNONYMOUS	Homeobox	TAGAAAGGCACCTTCCACTATTTC	ACACAAACTTCACCTCATCACAGT	NA
SNP0601	Ca_09222	*Ca_Kabuli_Chr07*	[G/A]	13059852	CDS_SYNONYMOUS	BTB/POZ-like	CAGAACAGAAAGAGATTCTGGAAA	AATTTCATGTCTTGTGCTTCTCAG	NA
SNP0602	Ca_16024	*Ca_Kabuli_Chr07*	[C/T]	13262430	CDS_SYNONYMOUS	SET domain	ATTCCTTTGCCAACAAGAAACTAC	GAAGATGAGCAAGAGCATCTCAG	NA
SNP0603	Ca_16026	*Ca_Kabuli_Chr07*	[A/G]	13319871	CDS_SYNONYMOUS	Homeobox	CTGAGATATTAGGAATGGCAATCA	GAATTACTAGAGCAGCAGCAACAG	NA
SNP0604	Ca_16044	*Ca_Kabuli_Chr07*	[A/C]	13605188	CDS_SYNONYMOUS	Zinc finger, C2H2-type	TTTGCAAATGGTAGTACTTGGCTA	CCAGTCTGCAATCTCAAACATTAG	NA
SNP0605	Ca_16064	*Ca_Kabuli_Chr07*	[T/C]	13904163	CDS_SYNONYMOUS	Tubby, C-terminal	ACCCATAAGTCCAAATGAGATTGT	GCACTATTGCATAGAGGAATTTGA	NA
SNP0606	Ca_23041	*Ca_Kabuli_Chr07*	[T/C]	14233368	CDS_SYNONYMOUS	Protein of unknown function DUF248, methyltransferase putative	GATAATCACCCCTTCAACATCAAT	TAATATCGAAGTTTGCAACGTCAC	NA
SNP0607	Ca_23042	*Ca_Kabuli_Chr07*	[T/C]	14282088	CDS_SYNONYMOUS	Protein of unknown function DUF702	CTCAGAGAGAGAGGTGTGACTTTG	CCATTGTTAGCAGGTATAGTTCCA	NA
SNP0608	Ca_09969	*Ca_Kabuli_Chr07*	[C/G]	14775135	CDS_SYNONYMOUS	Mediator complex, subunit Med4	CAAAGCAGTAACTAGGTGCTGAAA	CCCTGCTAGGTTAGGTCTTACAAA	NA
SNP0609	Ca_15849	*Ca_Kabuli_Chr07*	[A/G]	17597421	CDS_SYNONYMOUS	Protein of unknown function DUF1639	GCTTCTCATTTTCTGCTTGATGTA	TGCTACTCTGAATCACTTTCCAAG	NA
SNP0610	Ca_12308	*Ca_Kabuli_Chr07*	[A/G]	18796768	CDS_SYNONYMOUS	ZF-HD homeobox protein, Cys/His-rich dimerisation domain	TCTGTCATTTGTCGTAGGACTCAC	CAAAGTAGGCATGAACTCAAATGT	NA
SNP0611	Ca_12344	*Ca_Kabuli_Chr07*	[T/C]	19470527	CDS_SYNONYMOUS	AUX/IAA protein	ATACTTGCGATGATGTCATATTCC	TCTACTTTTGCGGATGAACTTACA	NA
SNP0612	Ca_10028	*Ca_Kabuli_Chr07*	[A/G]	32522201	CDS_SYNONYMOUS	SANT domain, DNA binding	CAGCTTTTCCATCTTGGACTTAAT	GTGTCAGTTTGCTCTTATCTTCCA	NA
SNP0613	Ca_10015	*Ca_Kabuli_Chr07*	[T/G]	32784903	CDS_SYNONYMOUS	Transcription initiation factor TFIID	GAGGTGTTTTACCATATCATGCAA	CAACACCAATGACATAGTGAAGGT	NA
SNP0614	Ca_10004	*Ca_Kabuli_Chr07*	[T/G]	32942951	CDS_SYNONYMOUS	Transcription factor GRAS	AGTACTACCACAAGCACAACATCC	GTACTGAATGGCAAGTCATGTTTC	NA
SNP0615	Ca_16177	*Ca_Kabuli_Chr07*	[C/T]	33573605	CDS_SYNONYMOUS	CCAAT-binding factor	TCTATGCATTCGACAACTAAAAGG	TAAAACGGACCAAAGTCTTAAACC	NA
SNP0616	Ca_17639	*Ca_Kabuli_Chr07*	[T/C]	35181661	CDS_SYNONYMOUS	Helix-loop-helix DNA-binding domain	AAACTTGCTGTTAGGAAAATGGAG	GGCTCAAAGTAGAAAAGTTGCATT	NA
SNP0617	Ca_17781	*Ca_Kabuli_Chr07*	[T/A]	35799083	CDS_SYNONYMOUS	BTB/POZ-like	CCTGAAAATCAAGATGAAGAGGTT	AGAACTGCAACAAAAGAAACAGGT	NA
SNP0618	Ca_13756	*Ca_Kabuli_Chr07*	[T/C]	36715249	CDS_SYNONYMOUS	Mediator complex, subunit Med12	AGACAGTTCATCAGTTCTGTCTGC	TCATCCAGATCAAGCACATAACTT	NA
SNP0619	Ca_19592	*Ca_Kabuli_Chr07*	[T/C]	39399280	CDS_SYNONYMOUS	Domain of unknown function DUF296	TTTGGATGTAATGATGGGTGTAAG	CTTAGTCGTGGTCAGAAACGAGAT	NA
SNP0620	Ca_25098	*Ca_Kabuli_Chr07*	[T/C]	39768248	CDS_SYNONYMOUS	Zinc finger, CCCH-type	ATGAGATGGAGACATGGAAGAGAC	CGAAATCCAGCTTTATATTTAGGG	NA

SNP IDs	*Kabuli* /*desi* gene accession IDs	*Kabuli* /*desi* chromosomes	SNPs	Physical positions (bp)	Sequence components of genome	Putative functions	Forward primer sequences (5'-3')	Reverse primer sequences (5'-3')	Primers used in Figure 2A
SNP0621	Ca_17996	*Ca_Kabuli_Chr07*	[A/C]	41387509	CDS_SYNONYMOUS	WD40 repeat	TCATGATACCACTTTGGCTTTTAG	CATCATACCCTACACCTCCAACTT	NA
SNP0622	Ca_17969	*Ca_Kabuli_Chr07*	[C/T]	42166004	CDS_SYNONYMOUS	Protein of unknown function DUF810	GAGAATCCTTTAGAGACCAGAACG	TTTAGGAATCACAGAGACAATCCA	NA
SNP0623	Ca_11909	*Ca_Kabuli_Chr08*	[A/C]	281630	CDS_SYNONYMOUS	Helix-loop-helix DNA-binding domain	GGCTGACTTTATGAACCACTTTCT	GTACGTTACGGAAGGTAAAATTGC	NA
SNP0624	Ca_11886	*Ca_Kabuli_Chr08*	[G/A]	510824	CDS_SYNONYMOUS	SNF2-related	TCTCATACTTTTGCTATGCCGATA	GTTACATTACAACTGCCAGAAGGA	NA
SNP0625	Ca_11866	*Ca_Kabuli_Chr08*	[A/C]	649491	CDS_SYNONYMOUS	Protein of unknown function DUF1423, plant	AGAGGATTTACCATTTGCATTCAG	TCATCAATCTCTGCTTCAAGTTTC	NA
SNP0626	Ca_11856	*Ca_Kabuli_Chr08*	[A/T]	709124	CDS_SYNONYMOUS	Protein of unknown function DUF1950	CGGTGGTATAGAGTGAAATGGAAT	AAATTGAGGAGAGACCACTGAAAC	NA
SNP0627	Ca_11855	*Ca_Kabuli_Chr08*	[G/A]	714974	CDS_SYNONYMOUS	Protein of unknown function DUF1950	GGGACCATCTTATTTTAAACATGC	CCTTAGGCTTAGTGCCTTATTGAA	NA
SNP0628	Ca_11839	*Ca_Kabuli_Chr08*	[C/T]	822976	CDS_SYNONYMOUS	Zinc finger, PHD-type	CTGTTGAAGATCAAGAAACAGGTG	TGGATTCACTCATAACCTAACCAA	NA
SNP0629	Ca_15077	*Ca_Kabuli_Chr08*	[C/T]	1077334	CDS_SYNONYMOUS	WD40 repeat	ATGGAACAACCACATACTCACAAC	TCAATGGTAATGGTGTACCTTGAG	NA
SNP0630	Ca_15049	*Ca_Kabuli_Chr08*	[C/T]	1262561	CDS_SYNONYMOUS	Zinc finger, CCCH-type	AGAAACCCTTCTTCACCTTCTTCT	AGGATGGCAAAAGACACATTACTT	NA
SNP0631	Ca_15035	*Ca_Kabuli_Chr08*	[A/C]	1382454	CDS_SYNONYMOUS	Zinc finger, PHD-type	TTCTGATGAGGTTGGTAATTGGTA	TCGACAGTTTGAAAGTTAAGACCA	NA
SNP0632	Ca_15024	*Ca_Kabuli_Chr08*	[T/C]	1477523	CDS_SYNONYMOUS	WD40 repeat	CTATTGATATGGGGTCCAAGAAAC	CTCTGAATCATTAAAGCAACCTCA	NA
SNP0633	Ca_02400	*Ca_Kabuli_Chr08*	[T/C]	2029384	CDS_SYNONYMOUS	Protein of unknown function DUF544	AAATTAGGATATCGCACAAAGAGG	CTTATTTCTCTGTCCCTGCATTTT	NA
SNP0634	Ca_02336	*Ca_Kabuli_Chr08*	[A/G]	2488709	CDS_SYNONYMOUS	Domain of unknown function DUF828	AGTGTCAAAAGAAAGGACAATGTG	CCTCTTGAGCCTATGGAGTTTTTA	NA
SNP0635	Ca_02278	*Ca_Kabuli_Chr08*	[A/G]	3041947	CDS_SYNONYMOUS	Protein of unknown function DUF947	AATGGGACAAATTATGACGTAACC	CATTGGACTGTAGCTTCCACTAAA	NA
SNP0636	Ca_02251	*Ca_Kabuli_Chr08*	[A/C]	3269085	CDS_SYNONYMOUS	Homeobox	TGAGCTAGATCAACCAAGTACGTC	GAAACTTAGGCTTACCAAACAACAA	NA
SNP0637	Ca_02217	*Ca_Kabuli_Chr08*	[T/C]	3601517	CDS_SYNONYMOUS	Zinc finger, TAZ-type	GGAGATTGAGAGAGTAAAGGTGCT	GCCTGTTCATGTAGTATGAAGTGA	NA
SNP0638	Ca_02182	*Ca_Kabuli_Chr08*	[G/C]	3911876	CDS_SYNONYMOUS	SANT domain, DNA binding	ATTAAACTGTTGCTCTGTCGTTTG	CCTCTTCCACCGTCTATCATAACT	NA
SNP0639	Ca_02167	*Ca_Kabuli_Chr08*	[A/C]	4042166	CDS_SYNONYMOUS	Protein of unknown function DUF869, plant	CCTATGTGTGCTCATGTATTTTCC	TGGAGGCTTAAATGAGAAGGATTA	NA
SNP0640	Ca_02166	*Ca_Kabuli_Chr08*	[C/T]	4052702	CDS_SYNONYMOUS	SET domain	AATGTCCAAGTCCCAGTATACCTC	TCCAACTGTGTTTGACACTACTGA	NA
SNP0641	Ca_02163	*Ca_Kabuli_Chr08*	[T/C]	4074135	CDS_SYNONYMOUS	Zinc finger, RING-type	GAGGAAGCAGCAGTAGTAACAACA	CCTTTTCCCACTCCTAATACAAAA	NA
SNP0642	Ca_02135	*Ca_Kabuli_Chr08*	[A/G]	4322058	CDS_SYNONYMOUS	Protein of unknown function DUF3651, TMEM131	TTCTCTCTCTCTTCCACCATTTTT	GGCAATCTTTATATTGGGTGAGAC	NA
SNP0643	Ca_02124	*Ca_Kabuli_Chr08*	[T/A]	4421629	CDS_SYNONYMOUS	Lateral organ boundaries, LOB	AATCCTCAAAGGTTCACATACGTT	TTTCACAAGATGCTAGGACAGCTA	NA
SNP0644	Ca_02034	*Ca_Kabuli_Chr08*	[G/A]	5197254	CDS_SYNONYMOUS	Homeobox	AGATCTCACACACACAATGGAAAT	TAGGTTCAATAACCGAGGCTTCTA	NA
SNP0645	Ca_02004	*Ca_Kabuli_Chr08*	[T/C]	5412489	CDS_SYNONYMOUS	Protein of unknown function DUF702	AGCTCATAAACTCTCTCTGCACAA	ACATCTAAACTCCGCTTGAGAACT	NA
SNP0646	Ca_10735	*Ca_Kabuli_Chr08*	[G/T]	6544248	CDS_SYNONYMOUS	Zinc finger, C2H2-type	CCCGGCTTATAGATATTTTCTTATC	TCTATCTTGGGGGATCAATATTCT	NA
SNP0647	Ca_10698	*Ca_Kabuli_Chr08*	[A/G]	6943710	CDS_SYNONYMOUS	ARID/BRIGHT DNA-binding domain	ACACTGGTCATCACAATTACCATC	TGTAGGAGGTTGTTGTGTTTTCAT	NA

SNP IDs	*Kabuli* /*desi* gene accession IDs	*Kabuli* /*desi* chromosomes	SNPs	Physical positions (bp)	Sequence components of genome	Putative functions	Forward primer sequences (5'-3')	Reverse primer sequences (5'-3')	Primers used in Figure 2A
SNP0648	Ca_10687	*Ca_Kabuli_Chr08*	[G/A]	7037715	CDS_SYNONYMOUS	Protein of unknown function DUF647	TGGAGAACTTGACTGGTTCTATGA	AACAGGCTCTTAGAATTTGGATTG	NA
SNP0649	Ca_10614	*Ca_Kabuli_Chr08*	[A/G]	7726879	CDS_SYNONYMOUS	Zinc finger, CCHC-type	TGAATCCGTATCAGAGTAATTGGA	GAAGCAACAGAACTGCTATACTGG	NA
SNP0650	Ca_10613	*Ca_Kabuli_Chr08*	[T/C]	7729693	CDS_SYNONYMOUS	Zinc finger, CCHC-type	TCGTTAGAGAGTAGGAGGGAAGAA	TCTAACTATTGGAACCCAACTTCC	NA
SNP0651	Ca_10604	*Ca_Kabuli_Chr08*	[T/C]	7807065	CDS_SYNONYMOUS	SET domain	CGTAACGCAAAGCTTCAAATAAAC	CATTTCCAACAAAACAAGTCCTTC	NA
SNP0652	Ca_11534	*Ca_Kabuli_Chr08*	[A/C]	9112172	CDS_SYNONYMOUS	Protein of unknown function DUF1399	ATTTAGAATGTGAGTGCAGTTGCT	TATGATCTGAGCATGGAACTCATT	NA
SNP0653	Ca_18416	*Ca_Kabuli_Chr08*	[T/G]	10258892	CDS_SYNONYMOUS	Zinc finger, PHD-type	GTCATGGTGTAAGTCTGATGCTTT	AAACAAAGCTAGAACCTGAAATGC	NA
SNP0654	Ca_16839	*Ca_Kabuli_Chr08*	[A/C]	11186986	CDS_SYNONYMOUS	Zinc finger, RanBP2-type	CCTAGATGCAATACTCGATTGGTC	TTTTCCAGAAGCAGTAACAAACAG	NA
SNP0655	Ca_16820	*Ca_Kabuli_Chr08*	[G/A]	11403805	CDS_SYNONYMOUS	SANT domain, DNA binding	ACGGCGTCTTTTAGTAGTTTGACT	TGTAGGGACAGGTAATGCATTTTA	NA
SNP0656	Ca_17888	*Ca_Kabuli_Chr08*	[A/C]	15555771	CDS_SYNONYMOUS	Protein of unknown function DUF1675	GAGCTCAATCTGTGAGTAATGGAA	ACAGGACCTCAAAACAACATTTCT	NA
SNP0657	Ca_15527	*Ca_Kabuli_Chr08*	[A/G]	16145419	CDS_SYNONYMOUS	Domain of unknown function DUF292, eukaryotic	GCAGCCAGTATCTTTATTTTGGTT	CGAGGATAAAGCTCTTGAGGAATA	NA
SNP0658	Ca_21518	*Ca_Kabuli_Scaffold_1281*	[G/A]	258565	CDS_SYNONYMOUS	Transcriptional factor B3	CCTATAGCGCATATTCCAAACTCT	AATGGTTGGATATGTTGGTAGTGA	NA
SNP0659	Ca_21523	*Ca_Kabuli_Scaffold_1281*	[A/G]	316037	CDS_SYNONYMOUS	Zinc finger, RING-type	GCAAAACTCACCCACCATTATATC	CCGAAAATCCCTTCTATTAATCAC	NA
SNP0660	Ca_22581	*Ca_Kabuli_Scaffold_134*	[C/T]	218290	CDS_SYNONYMOUS	SET domain	TTCGTCAAAACAGAGTCTCTTCAT	AAACCAGGATAATCTCCATACTGC	NA
SNP0661	Ca_08786	*Ca_Kabuli_Scaffold_1348_1*	[A/G]	950995	CDS_SYNONYMOUS	Zinc finger, CCCH-type	GATTCCGTTGCAGTTTCATACTC	AGACTGCTGAATTGTCACATCATT	NA
SNP0662	Ca_08787	*Ca_Kabuli_Scaffold_1348_1*	[G/A]	959780	CDS_SYNONYMOUS	Transcription factor jumonji/aspartyl beta-hydroxylase	GAGGATCATGATTTGCATAGTTTG	CTCATAATCGATTCCCATTTCTTC	NA
SNP0663	Ca_21995	*Ca_Kabuli_Scaffold_1351*	[G/A]	172111	CDS_SYNONYMOUS	Protein of unknown function DUF2419	ACTGCACAAGCATAATTCAATCAC	AGCAGCTGATCTATTTGAAGAACA	NA
SNP0664	Ca_21070	*Ca_Kabuli_Scaffold_157*	[T/A]	151131	CDS_SYNONYMOUS	Zinc finger, DHHC-type, palmitoyltransferase	AAAAACTTGACCTAGAAGGCTCAC	AAAATAGCAGATTGGGATTCAGAG	NA
SNP0665	Ca_26451	*Ca_Kabuli_Scaffold_2516*	[T/C]	58947	CDS_SYNONYMOUS	Domain of unknown function DUF125, transmembrane	TGCCCCAAATCTTGATTAGAGTAT	CATTAATTCAACTACGGATTCTGC	NA
SNP0666	Ca_26161	*Ca_Kabuli_Scaffold_2569*	[T/G]	58152	CDS_SYNONYMOUS	WD40 repeat	CAGTCAAACGAGCTTTGATTGTAG	TACATAAATTCAGCCCACCAAATC	NA
SNP0667	Ca_26888	*Ca_Kabuli_Scaffold_4011*	[G/C]	96223	CDS_SYNONYMOUS	Protein of unknown function DUF1296	ATGTGAAACGCACATAAATGACTC	TTCCTTAACTGCCTAGTGCCTACT	NA
SNP0668	Ca_22883	*Ca_Kabuli_Scaffold_421_2*	[C/A]	341095	CDS_SYNONYMOUS	Helix-loop-helix DNA-binding domain	AGTCTACTTCGCTTACCGATCAAC	GCAGTAGTAGGCTTTTCAGACACA	NA
SNP0669	Ca_22606	*Ca_Kabuli_Scaffold_451*	[A/G]	117231	CDS_SYNONYMOUS	HSA	AGCCTAAATCATTCTTCTCCTGTG	TAAGCATGTGCCAGAAGAACTAAC	NA
SNP0670	Ca_24566	*Ca_Kabuli_Scaffold_50*	[T/A]	153593	CDS_SYNONYMOUS	Protein of unknown function DUF3527	TCTGTGTAACAAATGGTTTTCGTC	GTGACTTCTTCGGTGTACCACTTT	NA
SNP0671	Ca_23923	*Ca_Kabuli_Scaffold_545*	[T/C]	138848	CDS_SYNONYMOUS	Zinc finger, C2H2-type	AAATTCCATGCACATATACCACTG	CCGAGACTATTATCGAAGAACCAG	NA
SNP0672	Ca_28031	*Ca_Kabuli_Scaffold_7355*	[C/G]	1606	CDS_SYNONYMOUS	CCT domain	CACCGCTATTTAAAAACACCGTA	TCAGTCTTTGCCTCCCTATTTATC	NA
SNP0673	Ca_23375	*Ca_Kabuli_Scaffold_812*	[T/C]	332746	CDS_SYNONYMOUS	Protein of unknown function DUF642	AGGACAAGGAGTCAAAACAGGTAA	GACGGCCATACCTGAATCTATATC	NA
SNP0674	Ca_22417	*Ca_Kabuli_Scaffold_845*	[G/A]	139268	CDS_SYNONYMOUS	Zinc finger, RING-type	TATTTTGTATCCTCCCACATAGCA	ACCGGTTGTGTGAAAAAGAAATAG	NA

SNP IDs	*Kabuli* /*desi* gene accession IDs	*Kabuli* /*desi* chromosomes	SNPs	Physical positions (bp)	Sequence components of genome	Putative functions	Forward primer sequences (5'-3')	Reverse primer sequences (5'-3')	Primers used in Figure 2A
SNP0675	Ca_27726	*Ca_Kabuli_Scaffold_C11175430*	[A/G]	987	CDS_SYNONYMOUS	Protein of unknown function DUF3743	AAATATGTCAGGAATTCCACCACT	ATACATGTTGCAATAGGGGTTTCT	NA
SNP0676	Ca_27691	*Ca_Kabuli_Scaffold_C11181782*	[A/G]	6215	CDS_SYNONYMOUS	Domain of unknown function DUF1084	CCTTGGATCACTTGTTCTATTTGA	TCGGGTTTATGTTTTAGGGTTAAG	NA
SNP0677	Ca_00372	*Ca_Desi_Chr01*	[T/C]	3675786	CDS_SYNONYMOUS	Auxin response factor 6	GTGGCAATGTAATAGCATTTTGAG	TTGCGTTTTAGCTGTGAAGTTTAG	NA
SNP0678	Ca_00550	*Ca_Desi_Chr01*	[C/T]	6399989	CDS_SYNONYMOUS	Homeobox-leucine zipper protein ATHB-14	ATACGATTGAGCACGAAGAGGTAT	CATCATCATCATTGAGAACTTTCC	NA
SNP0679	Ca_01851	*Ca_Desi_Chr02*	[A/C]	13100484	CDS_SYNONYMOUS	Scarecrow-like protein 3	TTTCTTTACTTGTCTTTGCTGTGC	GAGGTTCTTGAAACTGTGGATTCT	NA
SNP0680	Ca_01922	*Ca_Desi_Chr02*	[A/C]	14163542	CDS_SYNONYMOUS	Lysine-specific histone demethylase 1 homolog 3	GGAAAGAGCATAGCAACCTTATTC	GAAAATATCGGAGTGAGTGTGATG	NA
SNP0681	Ca_02202	*Ca_Desi_Chr03*	[G/A]	698068	CDS_SYNONYMOUS	Protein LHY	CATCAGTCTCTTCACTGCTAGAGG	ATTACACAGTGGAGCAAAGTATGG	NA
SNP0682	Ca_02392	*Ca_Desi_Chr03*	[T/C]	2986233	CDS_SYNONYMOUS	WD repeat-containing protein 48	TAGCACACTCGTTTCTTGTTCTTC	GAACACTTCCAGTGCAATTTACAG	NA
SNP0683	Ca_02541	*Ca_Desi_Chr03*	[A/T]	4356878	CDS_SYNONYMOUS	WD repeat domain phosphoinositide-interacting protein 3	TGCTCCTTCAACTTTCTTTTAACC	TTTGTCATTTCCTCACATCATACC	NA
SNP0684	Ca_02773	*Ca_Desi_Chr03*	[C/T]	8499689	CDS_SYNONYMOUS	Auxin response factor 8	GAATATTGTGCATGTGACATTGTG	TGTATGGCACACTAGAAGGGTTTA	NA
SNP0685	Ca_02786	*Ca_Desi_Chr03*	[G/C]	8826351	CDS_SYNONYMOUS	Transcription elongation factor SPT6	AAAACAGGTTAATAGAACGCCAGA	TATATTGTGGGTCTCATCTGTTGG	NA
SNP0686	Ca_03197	*Ca_Desi_Chr03*	[T/C]	15386074	CDS_SYNONYMOUS	Zinc finger CCCH domain-containing protein 5	GTTGCATATGCTTGAAATCTCTTG	CTTTTGATTGTCACAACATCACTG	NA
SNP0687	Ca_03610	*Ca_Desi_Chr03*	[C/T]	19827573	CDS_SYNONYMOUS	Bromodomain adjacent to zinc finger domain protein 1A	CTGTTGAAGATCAAGAAACAGGTG	TGGATTCACTCATAACCTAACCAA	NA
SNP0688	Ca_03691	*Ca_Desi_Chr03*	[C/T]	20603607	CDS_SYNONYMOUS	Transcription activator BRG1	GTTACATTACAACTGCCAGAAGGA	TCTCATACTTTTGCTATGCCGATA	NA
SNP0689	Ca_03895	*Ca_Desi_Chr04*	[C/T]	198665	CDS_SYNONYMOUS	Transcription factor RF2a	TACTATATGGATGCGACAATAGCC	ACTTTGGAGTGTTTGTGTTTGTGT	NA
SNP0690	Ca_04140	*Ca_Desi_Chr04*	[G/C]	3766974	CDS_SYNONYMOUS	CCT motif family protein	CCGAGACTATTATCGAAGAACCAG	AAATTCCATGCACATATACCACTG	NA
SNP0691	Ca_04363	*Ca_Desi_Chr04*	[T/C]	7647362	CDS_SYNONYMOUS	Lysine-specific demethylase 3B	GGACTTCAACCTTGAACTGGTATC	TCCGTATTTAAGACAGTTGGATGA	NA
SNP0692	Ca_04406	*Ca_Desi_Chr04*	[T/C]	8131392	CDS_SYNONYMOUS	Transcriptional corepressor LEUNIG	CAAGTAACAAAGCCACGGACTAAT	GTATGGTTGATGACAACATGGAAT	NA
SNP0693	Ca_04552	*Ca_Desi_Chr04*	[T/C]	9855603	CDS_SYNONYMOUS	Protein TIME FOR COFFEE	TAGCAGCATCCACTTTTTCTATTG	CAGAAGTGTGTCTTTGAGGAAGAA	NA
SNP0694	Ca_05622	*Ca_Desi_Chr05*	[A/G]	5193039	CDS_SYNONYMOUS	BEACH domain-containing protein lvsC	TGGTAAGTTTCATCTTCCTGCATA	AATGAGTCCAACATCATGTCCTTA	NA
SNP0695	Ca_05623	*Ca_Desi_Chr05*	[C/G]	5232897	CDS_SYNONYMOUS	GATA transcription factor 25	GAAGATGTTTATGTTTCCGGTGAT	GAAGGACAGCTGCTACAACATAGA	NA
SNP0696	Ca_05955	*Ca_Desi_Chr05*	[T/C]	8680914	CDS_SYNONYMOUS	Two-component response regulator-like PRR95	ACTTCCCTGATCAACAAAGAGAAC	TTATCGAATCAGAGTGTGTCAGGT	NA
SNP0697	Ca_07258	*Ca_Desi_Chr06*	[C/T]	10167738	CDS_SYNONYMOUS	Homeobox protein 10	CCTTGTACAGGGTTAGGAATGAAT	AAGTGCATTTAATCGCTCCTCTAC	NA
SNP0698	Ca_07479	*Ca_Desi_Chr07*	[T/A]	1467358	CDS_SYNONYMOUS	AP2-like ethylene-responsive transcription factor AIL1	GGATAATAGCTGCAGAAAAGAAGG	GAAGCTTGAAACAAAGGCAATAGT	NA
SNP0699	Ca_07522	*Ca_Desi_Chr07*	[A/G]	2069964	CDS_SYNONYMOUS	Transcription factor RF2a	TTAATCCTTCACAAATGCGTAGAC	GACAGAAATACCATCTCGGAGTTT	NA
SNP0700	Ca_07849	*Ca_Desi_Chr07*	[G/A]	6950249	CDS_SYNONYMOUS	Homeobox-leucine zipper protein HAT22	TAGAAAGGCACCTTCCACTATTTC	ACACAAACTTCACCTCATCACAGT	NA
SNP0701	Ca_07873	*Ca_Desi_Chr07*	[G/T]	7266190	CDS_SYNONYMOUS	SBP (S-ribonuclease binding protein) family protein	GGATAGAGAAGGAACAGAAACAGG	TTCTATATTTAACCCATCCGCTTC	NA

**SNP IDs**	***Kabuli* /*desi* gene accession IDs**	***Kabuli* /*desi* chromosomes**	**SNPs**	**Physical positions (bp)**	**Sequence components of genome**	**Putative functions**	**Forward primer sequences (5'-3')**	**Reverse primer sequences (5'-3')**	**Primers used in Figure 2A**
SNP0702	Ca_08285	*Ca_Desi_Chr08*	[C/T]	4722955	CDS_SYNONYMOUS	Two-component response regulator ARR2	AGTAGCAGACATTTCAGTGCTTTG	GCTAATGAAGGAACTTGGAGAAAC	NA
SNP0703	Ca_15142	*Ca_Desi_Scaffold_1174*	[C/T]	36613	CDS_SYNONYMOUS	NAC domain-containing protein 18	GGCCTGAATTCTAGTTAACGTCAT	GATGACAAAGTAGCAACGGTATTG	NA
SNP0704	Ca_09051	*Ca_Desi_Scaffold_1236*	[C/T]	338504	CDS_SYNONYMOUS	BEL1-like homeodomain protein 9	CTTGAAACTACCATAATGGGCAAT	ATAGGTCAGGTTTTCTTGATCAGC	NA
SNP0705	Ca_15415	*Ca_Desi_Scaffold_1282*	[C/T]	75640	CDS_SYNONYMOUS	BTB/POZ domain-containing protein At3g50780	AGGTGGCATTTAAGGGTTGTATAG	GGTTGGAGATGAAGAAGAAGAAAA	NA
SNP0706	Ca_15434	*Ca_Desi_Scaffold_1290*	[G/A]	33898	CDS_SYNONYMOUS	Transcription initiation factor TFIID subunit 7	GCAATTTAGTGAGGATGGAAGAAG	AACCCATCAAACACCTATTTTAGC	NA
SNP0707	Ca_15776	*Ca_Desi_Scaffold_1426*	[C/T]	64665	CDS_SYNONYMOUS	Zinc finger CCCH domain-containing protein 6	AAGACAAAGTTGCCCAGGTATAAG	TTGATATGCACAGTTAGCTCCATT	NA
SNP0708	Ca_15813	*Ca_Desi_Scaffold_1441*	[T/G]	27770	CDS_SYNONYMOUS	MATH domain-containing protein At5g43560	GATCTGCTACTGTCTACTGGCTTG	AAGTGTGGAAAATACTTGTGCTGA	NA
SNP0709	Ca_15904	*Ca_Desi_Scaffold_1484*	[A/C]	25798	CDS_SYNONYMOUS	BTB/POZ domain-containing protein NPY1	AGTTAGATGGTTGCCTGATTCTGT	CCTACCACTTTACACTTCAAAGCA	NA
SNP0710	Ca_11082	*Ca_Desi_Scaffold_158*	[G/A]	221533	CDS_SYNONYMOUS	Protein of unknown function (DUF620)	TCGTCCATTCATTGGTTACACTAT	AGAATGAAACAGTCCTCTTCGTTT	NA
SNP0711	Ca_16252	*Ca_Desi_Scaffold_1667*	[T/C]	33508	CDS_SYNONYMOUS	Zinc finger CCCH domain-containing protein 53	CTCAGTCACCTTCACCTTTTCTTT	ATCTTGAACAGGTCCATAAATGCT	NA
SNP0712	Ca_16997	*Ca_Desi_Scaffold_2142*	[T/A]	30207	CDS_SYNONYMOUS	Zinc finger CCCH domain-containing protein 7	ACTACCTGACTGATTTTTGCTTCC	CTAGGATTCAGGATGGTCAAGATT	NA
SNP0713	Ca_11558	*Ca_Desi_Scaffold_252*	[C/T]	87593	CDS_SYNONYMOUS	B3 domain-containing transcription factor ABI3	AATGGTTGGATATGTTGGTAGTGA	CCTATAGCGCATATTCCAAACTCT	NA
SNP0714	Ca_17653	*Ca_Desi_Scaffold_2603*	[C/T]	4601	CDS_SYNONYMOUS	Transcriptional corepressor SEUSS	GCCTTCTCTTTTGAGATCAAATTC	CAGTGCCATACATCCTGAAAATAA	NA
SNP0715	Ca_11735	*Ca_Desi_Scaffold_271*	[C/T]	33434	CDS_SYNONYMOUS	Tubby-like F-box protein 8	TGTTTCTTTGAAGCAGGTGAGATA	TAGGGTATCGATAGTCCATTGTGA	NA
SNP0716	Ca_17918	*Ca_Desi_Scaffold_2850*	[C/A]	6581	CDS_SYNONYMOUS	Mediator of RNA polymerase II transcription subunit 18	TGTATAAAGTGCTTGCCTCAGTGT	CACATAATAACCATTTCCGACTGA	NA
SNP0717	Ca_10605	*Ca_Desi_Scaffold_32*	[G/C]	80728	CDS_SYNONYMOUS	Probable WRKY transcription factor 19	ATCTCTGCTGATTTTCTCTCCAAT	CTACAAGCAGCAACAGATGAACTT	NA
SNP0718	Ca_18458	*Ca_Desi_Scaffold_3563*	[G/C]	1235	CDS_SYNONYMOUS	C2H2-like zinc finger protein	GAAAGGTACCAACTTTCCTTTGTC	ACGCCACTGTCTCTAAAACTTACC	NA
SNP0719	Ca_12314	*Ca_Desi_Scaffold_364*	[T/C]	38866	CDS_SYNONYMOUS	Myb-related protein 3R-1	TGCAAAGAGATGGTACAGATCCTA	GGACACTCTGGTTCAAAGCTATTT	NA
SNP0720	Ca_18943	*Ca_Desi_Scaffold_4448*	[C/T]	15603	CDS_SYNONYMOUS	Protein of unknown function (DUF810)	CTTGTGGTGCAAATATATCAGAGG	GCGGTTTTAATTGCTATACGAGAC	NA
SNP0721	Ca_13425	*Ca_Desi_Scaffold_614*	[A/G]	105277	CDS_SYNONYMOUS	Protein of unknown function, DUF593	CACCCAATTGAGTAAGTTGTTTCA	CACTATATTCACCCCACAGCATAA	NA
SNP0722	Ca_19827	*Ca_Desi_Scaffold_7085*	[A/G]	5369	CDS_SYNONYMOUS	Auxin response factor 2	TGTAGGTCCAGTCCTTGTTACTCA	GTGTATTGTCAGGTGTTGTTGGTT	NA
SNP0723	Ca_10810	*Ca_Desi_Scaffold_93*	[T/C]	155942	CDS_SYNONYMOUS	Protein of unknown function (DUF1644)	CCCAGAGTCTAGATGGTCTCCTAA	GTTGAATTGCCGATGTATTACAAG	NA
SNP0724	Ca_14531	*Ca_Desi_Scaffold_940*	[A/C]	27874	CDS_SYNONYMOUS	Protein of unknown function (duplicated DUF1399)	ATTTAGAATGTGAGTGCAGTTGCT	TATGATCTGAGCATGGAACTCATT	NA
SNP0725	Ca_28178	*Ca_Kabuli_Scaffold_3337*	[C/T]	18022	DRR	Domain of unknown function DUF828	GACCGATTTTCTCCATTTAGTGAC	GCTGTGGAACCTTGGTTATAGATT	NA
SNP0726	Ca_27768	*Ca_Kabuli_Scaffold_799*	[C/T]	3928	DRR	Domain of unknown function DUF828	GTTACCTAGTGAACAGGGGAAAGA	TGTATTTAGTGGTTGAAAGCGTCT	NA
SNP0727	Ca_28165	*Ca_Kabuli_Scaffold_C11181840*	[A/G]	19192	DRR	Domain of unknown function DUF547	GTTACCTAGTGAACAGGGGAAAGA	TGTATTTAGTGGTTGAAAGCGTCT	NA
SNP0728	Ca_00149	*Ca_Desi_Chr01*	[T/C]	1480187	DRR	Transcription factor GTE7	AACTCCAACTTGTATTGGTTGGTC	GATGAAGATGACGATGATGAAGAC	NA

**SNP IDs**	***Kabuli* /*desi* gene accession IDs**	***Kabuli* /*desi* chromosomes**	**SNPs**	**Physical positions (bp)**	**Sequence components of genome**	**Putative functions**	**Forward primer sequences (5'-3')**	**Reverse primer sequences (5'-3')**	**Primers used in Figure 2A**
SNP0729	Ca_00159	*Ca_Desi_Chr01*	[C/T]	1598211	DRR	Protein FAR1-RELATED SEQUENCE 11	GGATGCGTGTATGATCTCATCTAA	ACTGAAGACACCATTCCTAAAAGG	NA
SNP0730	Ca_01546	*Ca_Desi_Chr02*	[C/G]	7082948	DRR	ABSCISIC ACID-INSENSITIVE 5-like protein 2	CGATCTACAATCTTGTTGAAAACG	GAGATACTCACCGAAACCATCATT	NA
SNP0731	Ca_02518	*Ca_Desi_Chr03*	[T/G]	4174824	DRR	Unknown expressed protein	GCGTGCACAGAGTTATGTATTGAT	ATCATATTTGTTGCTTGCTGTGAG	NA
SNP0732	Ca_03068	*Ca_Desi_Chr03*	[T/G]	13208922	DRR	far-red elongated hypocotyl 1	CCTACGATGATCTCGATACACAAG	AACGTGACACTTTATAGCCCTTTT	NA
SNP0733	Ca_04017	*Ca_Desi_Chr04*	[T/C]	2042496	DRR	Protein of unknown function (DUF620)	AGCTAAGATTTGCTTCGGTTAGTG	GTGATTCGTGGATCAAAAGTGTAA	NA
SNP0734	Ca_06184	*Ca_Desi_Chr05*	[C/T]	11335385	DRR	SMAD/FHA domain-containing protein	TGGAAACTTAGTTGCAGAAATCAG	AGGCACTTGTTATTACATTGACCA	NA
SNP0735	Ca_07787	*Ca_Desi_Chr07*	[C/G]	6252949	DRR	zinc finger (Ran-binding) family protein	TAATTCAAATGTCTGTTCCACGTC	CAACTTCTTGTTAGATGCAACCAT	NA
SNP0736	Ca_08198	*Ca_Desi_Chr08*	[A/G]	3940158	DRR	SWI/SNF-related matrix-associated actin-dependent regulator of chromatin subfamily A-like protein 6	ATTTCTTAGGGACGTAGTGCAAAG	GCTAGGTTTTTAGGGGAGAATCAT	NA
SNP0737	Ca_17150	*Ca_Desi_Scaffold_2238*	[A/G]	37374	DRR	Zinc finger with UFM1-specific peptidase domain protein	TCAACCATGCTATCTATGGCTCTA	AGGTGTTGGGTTGAAGTAAAGAAG	NA
SNP0738	Ca_17182	*Ca_Desi_Scaffold_2259*	[T/A]	28398	DRR	Transcription factor MYB86	TTTAACGGTGCATCACTAAGAAAG	ATGGTATCACAACTGCAATTTAGG	NA
SNP0739	Ca_08979	*Ca_Desi_Scaffold_233*	[A/C]	50084	DRR	Protein of unknown function (DUF679)	ACTTGCATCCCTAAGTTGTCTTTC	CTAACCACACATCTCAATCACACA	NA
SNP0740	Ca_10605	*Ca_Desi_Scaffold_32*	[T/C]	78989	DRR	Probable WRKY transcription factor 19	TTTAACTCCTGAAGAACCATCTCC	ATTGGAGAGAAGATTAGCAGCAAG	NA
SNP0741	Ca_19685	*Ca_Desi_Scaffold_6521*	[T/C]	5184	DRR	WD40 repeat-containing protein SMU1	ATTCATATCCTCTGGTTTGATTCC	TCACATATGATAATGGTTGCACTG	NA
SNP0742	Ca_04140	*Ca_Desi_Chr04*	[T/C]	3764973	URR	CCT motif family protein	TTCCACCTTGAACTTAGTGTATGC	ATGAGTCTATGAAGCACTGACACC	NA
SNP0743	Ca_04272	*Ca_Desi_Chr04*	[T/G]	5949743	URR	BED zinc finger; hAT family dimerisation domain	TTTGTTAGAGTGAATGTTGGCTGT	GATGCATGATGTTAACCACACTTT	NA
SNP0744	Ca_05237	*Ca_Desi_Chr04*	[C/A]	22037221	URR	Protein FAR1-RELATED SEQUENCE 11	TCCCGATAAATTCAACTGTCACTA	GCTTCACATTGACAATTTATGTCC	NA
SNP0745	Ca_17178	*Ca_Desi_Scaffold_2257*	[A/G]	32638	URR	Protein of unknown function, DUF617	CCACAGAAAAAGGAAGATCTCAAT	GGCATACATGGCTTTGTAGTTAGA	NA
SNP0746	Ca_12419	*Ca_Desi_Scaffold_385*	[T/C]	167051	URR	High mobility group B protein 7	CCATCAAGAACAGATATTGCAAAG	CGTGTCTAGAGAGTCCAAAAACAG	NA
SNP0747	Ca_13660	*Ca_Desi_Scaffold_680*	[C/T]	16012	URR	Lysine-specific demethylase 5D	ATTCAGGTTCCATGGGTTAGTAGA	TTGTTATCTTTCCCAGACACTACC	NA
**SNP0748**	**Ca_20116**	***Ca_Desi_Scaffold_8872***	**[C/T]**	**3778**	**URR**	**Zinc finger CCCH domain-containing protein**	**CTGAGGTAGCATCAACAAAAACAT**	**CTGTAAAAGAAAGGGCAAATGAAG**	NA
SNP0749	Ca_10810	*Ca_Desi_Scaffold_93*	[G/C]	157340	URR	Protein of unknown function (DUF1644)	TTGCAAACTACTACTCCAGGAATG	TCAGTCCTCAACACCCCTACTAAT	NA
SNP0750	Ca_TC07446	*Ca_Desi_Chr01*	[C/T]	1661476	CDS_NON_SYNONYMOUS	GeBP	GCTCAATCCGTTTAAGCCAGT	CCCTAACACCAATTCTCGATG	NA
SNP0751	Ca_TC05193	*Ca_Desi_Chr01*	[T/C]	1827593	CDS_NON_SYNONYMOUS	Jumonji	TGAGCACACAGATGACAGTGA	TACGAACACAGGCTCCAAAG	NA
SNP0752	Ca_TC04526	*Ca_Desi_Chr01*	[G/A]	2188544	CDS_NON_SYNONYMOUS	SNF2	ACTGCTGGCAGTTCCTCTGT	CTGTTCAGTGTGGCTTTGGA	NA
SNP0753	Ca_TC17243	*Ca_Desi_Chr01*	[A/T]	2602506	CDS_NON_SYNONYMOUS	SBP	AGTGATCATTGGTCATTAAAGCA	AAAGGTGGGGTGAGGAAAAG	NA
SNP0754	Ca_TC11301	*Ca_Desi_Chr01*	[G/A]	2631901	CDS_NON_SYNONYMOUS	C3H	TCTAGGGCCACCAAGTCATC	TATCTTCGGTGGTCGGAATC	NA
SNP0755	Ca_TC17851	*Ca_Desi_Chr01*	[G/C]	3650162	CDS_NON_SYNONYMOUS	MADS	TGTTTCCTCATCAAAGCTTGC	CAAGTGAAGGTGTCTTCTCCA	NA

SNP IDs	*Kabuli* /*desi* gene accession IDs	*Kabuli* /*desi* chromosomes	SNPs	Physical positions (bp)	Sequence components of genome	Putative functions	Forward primer sequences (5'-3')	Reverse primer sequences (5'-3')	Primers used in Figure 2A
SNP0756	Ca_TC03663	*Ca_Desi_Chr01*	[G/A]	3734533	CDS_NON_SYNONYMOUS	Bromodomain	AGGCTGTCCAGGTGATCTGT	GGGCAGCTTCTCTTTCCTTT	NA
SNP0757	Ca_TC11111	*Ca_Desi_Chr01*	[G/C]	4325063	CDS_NON_SYNONYMOUS	mTERF	TCTCCAATTTTCCCCACAAA	GCAACTTGCACATCAGTGCT	NA
SNP0758	Ca_TC15990	*Ca_Desi_Chr01*	[T/C]	4379384	CDS_NON_SYNONYMOUS	SNF2	CGTCTGGGACACCTCAAAAT	CAAGTTGATTTGCAGGCTCA	NA
SNP0759	Ca_TC07591	*Ca_Desi_Chr01*	[G/T]	4866342	CDS_NON_SYNONYMOUS	BSD	GCGATCTCGAAGATTTCAGC	AGCCACATAACATGGTGCAA	NA
SNP0760	Ca_TC17358	*Ca_Desi_Chr01*	[C/G]	5452945	CDS_NON_SYNONYMOUS	MYB-related	CCCATTAAAAGAGCTCATATAGCAA	AACCTATGCTAGTTTGCTTATCCCTA	NA
SNP0761	Ca_TC08361	*Ca_Desi_Chr01*	[T/C]	5666051	CDS_NON_SYNONYMOUS	WRKY	CTTGTTTGCGAACTGGACAA	CGATCCAAGTTTCCACCATT	NA
SNP0762	Ca_TC14813	*Ca_Desi_Chr01*	[G/A]	6592726	CDS_NON_SYNONYMOUS	FHA	TCTCTCTCTGCTCCGCTTCT	AGTCACCCGAACAAATCGTC	NA
SNP0763	Ca_TC01171	*Ca_Desi_Chr01*	[A/T]	7417345	CDS_NON_SYNONYMOUS	HB	TGCAAAACCCTGTTGCATTA	GTCCAACAGGACCTCCTCAA	NA
SNP0764	Ca_TC15349	*Ca_Desi_Chr01*	[G/A]	7436472	CDS_NON_SYNONYMOUS	CCAAT	CTCAAGATTTGGGTGGGTGT	GGGGTTGTACAAAGCCAGAA	NA
SNP0765	Ca_TC11727	*Ca_Desi_Chr01*	[G/C]	8284578	CDS_NON_SYNONYMOUS	MYB-related	TGTTGTATCTAAGCCAATTCACATCC	GCTTCAAGTACTTTATTGCATCTC	NA
SNP0766	Ca_TC04704	*Ca_Desi_Chr01*	[G/A]	9654075	CDS_NON_SYNONYMOUS	Trihelix	GTTACCCTCACCCCAAAGGT	CAATCTGCTGCCATTTTTCA	NA
SNP0767	Ca_TC13639	*Ca_Desi_Chr01*	[G/C]	10455472	CDS_NON_SYNONYMOUS	MYB	ACTTGCTGGATTGCAAAGGT	CCCTTCAAATGCATACATGG	NA
SNP0768	Ca_TC10361	*Ca_Desi_Chr01*	[G/A]	11285630	CDS_NON_SYNONYMOUS	GRAS	AGGCATGCTAAATTGCCAAC	CGCTGTGTGCTGAAGAAAAA	NA
SNP0769	Ca_TC04427	*Ca_Desi_Chr01*	[G/A]	12530205	CDS_NON_SYNONYMOUS	AP2-EREBP	GGGAGCTTGTGAGGATTTGA	CTCGAATTCGCAACAACAAA	NA
SNP0770	Ca_TC10826	*Ca_Desi_Chr01*	[G/C]	12928919	CDS_NON_SYNONYMOUS	G2-like	TGTGGCTGTGAGAGATCCAA	TCCCGTGTACTCGTGCATAA	NA
SNP0771	Ca_TC19580	*Ca_Desi_Chr01*	[C/T]	13679988	CDS_NON_SYNONYMOUS	AP2-EREBP	GGCACAACACAAGAATCCAG	CTTTACCAACGCAACGCAAT	NA
SNP0772	Ca_TC14890	*Ca_Desi_Chr01*	[T/C]	13801427	CDS_NON_SYNONYMOUS	bHLH	ACACTCTCCCCTGCAAAGAA	CACGACGCTTAGTGTTCCAA	NA
SNP0773	Ca_TC08193	*Ca_Desi_Chr01*	[T/C]	13994815	CDS_NON_SYNONYMOUS	HB	ATGCCTTCAATGATCGGAAC	CACCAAATGCATGTCCAGAG	NA
SNP0774	Ca_TC11910	*Ca_Desi_Chr01*	[A/C]	15378650	CDS_NON_SYNONYMOUS	bHLH	TGGACGAAATGAATGTTCACC	TTTCTAGCAATCTTCACGTGGT	NA
SNP0775	Ca_TC18264	*Ca_Desi_Chr01*	[A/C]	15606770	CDS_NON_SYNONYMOUS	WRKY	CCAATCCCAATCCCTTCTTT	CAATGCAAAGCTGGACAAAA	NA
SNP0776	Ca_TC14070	*Ca_Desi_Chr01*	[G/T]	16080907	CDS_NON_SYNONYMOUS	GRAS	CAGGAGCAACACAACTCAGG	CAACATTGCCCCATCTTTCT	NA
**SNP0777**	**Ca_TC09197**	***Ca_Desi_Chr01***	**[T/C]**	**16680465**	**CDS_NON_SYNONYMOUS**	**NAC**	**TGCAGCCAATTGCTATTTCA**	**AGGTTCACCCCAGCTACAAC**	NA
SNP0778	Ca_TC08115	*Ca_Desi_Chr01*	[T/C]	17405553	CDS_NON_SYNONYMOUS	CAMTA	AGGCTGAAGTTTTTGCCTCA	GCATCAGAAGCATCCATCAA	NA
SNP0779	Ca_TC05008	*Ca_Desi_Chr01*	[A/C]	18460430	CDS_NON_SYNONYMOUS	mTERF	CGCAAAAATCATCTTCCGTA	ATGACATGCTGGTTTTGTCC	NA
SNP0780	Ca_TC10403	*Ca_Desi_Chr01*	[T/C]	20598392	CDS_NON_SYNONYMOUS	C3H	TGTGGTGGAAGAATGGAACA	TACTTCCACCCATCCCAGTC	NA
SNP0781	Ca_TC11725	*Ca_Desi_Chr01*	[C/A]	21135977	CDS_NON_SYNONYMOUS	MADS	AGCTTTTGAGTACGTCCGTGA	TCCCTTTTCACCACAAAAGC	NA
SNP0782	Ca_TC17860	*Ca_Desi_Chr01*	[T/C]	23850218	CDS_NON_SYNONYMOUS	AP2-EREBP	TCCGGCACATAGAAATCACC	CCGAAAGCAAAGATCACATTC	NA

**SNP IDs**	***Kabuli*/*desi* gene accession IDs**	***Kabuli*/*desi* chromosomes**	**SNPs**	**Physical positions (bp)**	**Sequence components of genome**	**Putative functions**	**Forward primer sequences (5'-3')**	**Reverse primer sequences (5'-3')**	**Primers used in Figure 2A**
SNP0783	Ca_TC08151	*Ca_Desi_Chr01*	[G/A]	24068638	CDS_NON_SYNONYMOUS	ARF	GCCCCTGTGAATTTGTGATT	TATTCTGCGGATGGTTCACA	NA
SNP0784	Ca_TC18345	*Ca_Desi_Chr01*	[T/C]	25186105	CDS_NON_SYNONYMOUS	Tify	GTGGTTTGGCCACTACGAAT	TTGGAACATCAACGCAACAT	NA
SNP0785	Ca_TC02024	*Ca_Desi_Chr01*	[A/C]	26026317	CDS_NON_SYNONYMOUS	SBP	CCTGTACCGTCCTTTGCATT	CAGTTGCCGCAGAGACTACA	NA
SNP0786	Ca_TC03684	*Ca_Desi_Chr01*	[C/T]	33623744	CDS_NON_SYNONYMOUS	SBP	TGGAGATGCAATTGGTTCAA	TGTCACACTTTGATACATCTCAGG	NA
SNP0787	Ca_TC04386	*Ca_Desi_Chr01*	[G/T]	37691837	CDS_NON_SYNONYMOUS	G2-like	CATCCAAATGGTGAGCCTCT	TCGTCTTCGTTGGACTGTTG	NA
SNP0788	Ca_TC17995	*Ca_Desi_Chr01*	[G/T]	41981594	CDS_NON_SYNONYMOUS	AP2-EREBP	CCAGCCCAATCAGACATTG	ACAAAGCGTGCTCGTTATCA	NA
SNP0789	Ca_TC15541	*Ca_Desi_Chr01*	[G/C]	43957759	CDS_NON_SYNONYMOUS	NAC	AAGCATGGTAGAGGCAAAGC	CCTTGCATGTTTTCTCATCG	NA
**SNP0790**	**Ca_TC09680**	***Ca_Desi_Chr01***	**[C/G]**	**46718906**	**CDS_NON_SYNONYMOUS**	**mTERF**	**GGCCTCAGAACCTCCTCTATGCTG**	**CAAGATTGAGGGTCGTTTTGTGG**	**Pr4**
SNP0791	Ca_TC02465	*Ca_Desi_Chr01*	[T/C]	48002347	CDS_NON_SYNONYMOUS	PHD	CAAGCTTGAAGGAGGCAAAG	ACACCGAAGGGAAAGCCTAT	NA
SNP0792	Ca_TC06936	*Ca_Desi_Chr01*	[T/C]	48218880	CDS_NON_SYNONYMOUS	Alfin-like	CAAATGTTGAACAAGGTTTGATGT	CCATTTTCAAAAGCTTCAACC	NA
SNP0793	Ca_TC05036	*Ca_Desi_Chr01*	[C/A]	48249846	CDS_NON_SYNONYMOUS	bZIP	GATTTGCGGTTGTTTGTGTG	AAGCTTGTCCTGAGCCAATG	NA
SNP0794	Ca_TC16216	*Ca_Desi_Chr02*	[G/A]	355965	CDS_NON_SYNONYMOUS	LOB	CCCTTAAGCCGTTTATGGAA	CGCCTCCATCAAAATCTCAT	NA
SNP0795	Ca_TC12565	*Ca_Desi_Chr02*	[A/T]	3202163	CDS_NON_SYNONYMOUS	HB	TCATGACTCAAGTAGATTTGTCTCAAC	CATCATCACTGCCATCCCTA	NA
SNP0796	Ca_TC17714	*Ca_Desi_Chr02*	[G/A]	3353616	CDS_NON_SYNONYMOUS	MYB	TCTAGAAGCCATTTCTCAAGCA	GGACCATGGACTCCTGAAGA	NA
SNP0797	Ca_TC14300	*Ca_Desi_Chr02*	[G/A]	3410435	CDS_NON_SYNONYMOUS	zf-HD	CACAGAGGTTTGAAGCAAGAAA	CACTCCCAAACTCTCAAGTAGCA	NA
SNP0798	Ca_TC12111	*Ca_Desi_Chr02*	[A/C]	4324332	CDS_NON_SYNONYMOUS	SAP	CATGAAACCACAATGTGCAA	TTCAATTCCACCACCAAACC	NA
SNP0799	Ca_TC01787	*Ca_Desi_Chr02*	[A/T]	5886240	CDS_NON_SYNONYMOUS	PHD	TAAAACCTCGCGGAAGAGAA	AAGATTCCCGAATCCAAACC	NA
SNP0800	Ca_TC05633	*Ca_Desi_Chr02*	[T/C]	6181000	CDS_NON_SYNONYMOUS	SET	AACAACTCGCCAACATTTCC	CCCGAATCTTGTTTCACGTT	NA
SNP0801	Ca_TC23674	*Ca_Desi_Chr02*	[C/T]	6743024	CDS_NON_SYNONYMOUS	FAR1	ACGACGTTCACTTTCCATCC	GTCGGATTTGTCTCCGAAAG	NA
SNP0802	Ca_TC07875	*Ca_Desi_Chr02*	[G/A]	9152974	CDS_NON_SYNONYMOUS	bHLH	CTACACCGGAACCGTGTCTC	CGGATCACTTCCACATACCA	NA
SNP0803	Ca_TC23383	*Ca_Desi_Chr02*	[T/C]	9266035	CDS_NON_SYNONYMOUS	GNAT	ATGCCTGAAACCAATCTCCA	GTGGACCCCATCGTATGTTT	NA
**SNP0804**	**Ca_TC11796**	***Ca_Desi_Chr02***	**[T/C]**	**10330226**	**CDS_NON_SYNONYMOUS**	**bHLH**	**TGTGAAGATTGTCTGAAGTGGCGC**	**TCAGGTGGCATGGTGAGCTCT**	**Pr5**
SNP0805	Ca_TC06938	*Ca_Desi_Chr02*	[G/A]	14248647	CDS_NON_SYNONYMOUS	GNAT	TGCTTATCGTCAAATTCAATAAAAA	AATTCAATTCAACTCAACTCCAC	NA
SNP0806	Ca_TC17363	*Ca_Desi_Chr02*	[G/A]	15817338	CDS_NON_SYNONYMOUS	C2C2-YABBY	CAACGAGGAGAAACTCACCA	TGCTCCAATATTCTCTCCCAAT	NA
SNP0807	Ca_TC06463	*Ca_Desi_Chr02*	[G/A]	16132823	CDS_NON_SYNONYMOUS	SBP	GAGCTTGCATATTTGAACCCTA	TCATGTTGACTCACGCTCTG	NA
SNP0808	Ca_TC06445	*Ca_Desi_Chr02*	[G/C]	16353473	CDS_NON_SYNONYMOUS	GeBP	ACCCTCTTCCAAAACCCTTG	TTCAGTCGTGGTTACATCCAA	NA
SNP0809	Ca_TC17258	*Ca_Desi_Chr02*	[T/C]	18418063	CDS_NON_SYNONYMOUS	GNAT	TGCGAGCGCATGATACATA	CCACAACACACACTTTTGCAG	NA

SNP IDs	*Kabuli* /*desi* gene accession IDs	*Kabuli* /*desi* chromosomes	SNPs	Physical positions (bp)	Sequence components of genome	Putative functions	Forward primer sequences (5'-3')	Reverse primer sequences (5'-3')	Primers used in Figure 2A
SNP0810	Ca_TC05208	*Ca_Desi_Chr02*	[T/C]	18878024	CDS_NON_SYNONYMOUS	bHLH	GCTACAAAAGCCGTGTCTCC	TAAAATATGGCCAGCCCAAC	NA
SNP0811	Ca_TC10019	*Ca_Desi_Chr02*	[C/G]	21714168	CDS_NON_SYNONYMOUS	zf-HD	TTGGTGAAAGTGTGAAACTATGTG	TCCTCAATTAGGGTTTCTTACCA	NA
SNP0812	Ca_TC00533	*Ca_Desi_Chr02*	[T/C]	24236619	CDS_NON_SYNONYMOUS	Alfin-like	GGGGGAATATGTCAGCAAGA	GCACACCGAAGATCAGCATA	NA
SNP0813	Ca_TC14524	*Ca_Desi_Chr02*	[G/C]	25291561	CDS_NON_SYNONYMOUS	C3H	CATTGCTGAACCAATTGCAG	TCGACTGAACACAACCTGCT	NA
SNP0814	Ca_TC11707	*Ca_Desi_Chr02*	[T/C]	26158345	CDS_NON_SYNONYMOUS	FHA	CCACCACGCTCGTATCTTCT	GCAGAAGGCAACCAAGTGTT	NA
SNP0815	Ca_TC11204	*Ca_Desi_Chr02*	[A/G]	27361120	CDS_NON_SYNONYMOUS	bZIP	ATGTCGAGTCCCTGAAAACG	CAAACACCGCGTCACAGTTA	NA
SNP0816	Ca_TC06288	*Ca_Desi_Chr02*	[G/C]	27694185	CDS_NON_SYNONYMOUS	AP2-EREBP	ACTCTCTTTCCCGCCAAATC	CAATAAATCACCGCTTCATGG	NA
SNP0817	Ca_TC12651	*Ca_Desi_Chr02*	[G/A]	30377757	CDS_NON_SYNONYMOUS	MYB	GCTTCCTTAAGCTTCAAGTCATCT	AAACAACCTTTTTGTGGTTTAAGG	NA
SNP0818	Ca_TC16553	*Ca_Desi_Chr02*	[G/A]	30698052	CDS_NON_SYNONYMOUS	HMG	CACCTTTCCTAATCCGAGCA	CTTACACCCAGGGGACGATA	NA
SNP0819	Ca_TC05305	*Ca_Desi_Chr02*	[C/T]	30867757	CDS_NON_SYNONYMOUS	C3H	TCACGTAGGAAGGTGTTGGA	GGCGCCATACATCTCAGAAT	NA
SNP0820	Ca_TC00798	*Ca_Desi_Chr02*	[G/A]	32769483	CDS_NON_SYNONYMOUS	FAR1	ATGCTGTGCTGGGACTCTCT	AACTTTTTGTGGCCTTCACG	NA
SNP0821	Ca_TC07826	*Ca_Desi_Chr02*	[G/C]	32958106	CDS_NON_SYNONYMOUS	bZIP	TCAAATTCGATACCCCACAAA	GCTGAAGCCTATGCACATCA	NA
SNP0822	Ca_TC03320	*Ca_Desi_Chr02*	[A/T]	33604180	CDS_NON_SYNONYMOUS	SRS	CACCGGAGTTTGGTCTAGGT	GCAGCTGCATAAACATCGTG	NA
SNP0823	Ca_TC11772	*Ca_Desi_Chr02*	[T/C]	33661127	CDS_NON_SYNONYMOUS	HB	ACTCCTGCGGAAGCAGTAAG	GCACAACAAACAGCAAATGG	NA
SNP0824	Ca_TC04916	*Ca_Desi_Chr02*	[T/C]	33727257	CDS_NON_SYNONYMOUS	SNF2	AGAACGTGGAGCAAGAGCAT	ACTCGTGCAAGTCGAGAGGT	NA
SNP0825	Ca_TC06616	*Ca_Desi_Chr02*	[T/C]	34132454	CDS_NON_SYNONYMOUS	NAC	TTGAAAAATGGATGTGAGCAA	TGGTTTGCTTTCATTTTGTTG	NA
SNP0826	Ca_TC08159	*Ca_Desi_Chr02*	[G/T]	34853120	CDS_NON_SYNONYMOUS	Bromodomain	ACAGGAACCTGTTGGCTCTG	CCAACTGGGTGAATGTGTTG	NA
SNP0827	Ca_TC06161	*Ca_Desi_Chr02*	[G/A]	35412866	CDS_NON_SYNONYMOUS	mTERF	GCACATGACATGGCTTTGTT	TTCCTTTTTCAAACGGACCA	NA
SNP0828	Ca_TC12031	*Ca_Desi_Chr02*	[G/A]	35556870	CDS_NON_SYNONYMOUS	CCAAT	TCCATTCCCTCCATCTTCAC	AAGCAGAGAAAGGATCGTTGA	NA
SNP0829	Ca_TC06005	*Ca_Desi_Chr02*	[G/A]	36087743	CDS_NON_SYNONYMOUS	GRAS	TCGCTTGAAATGGACAGTTG	TTGAATGCTTTGCAGTTTGC	NA
SNP0830	Ca_TC11918	*Ca_Desi_Chr02*	[G/A]	36337365	CDS_NON_SYNONYMOUS	GNAT	GAGTCGCACCCAATTCAACT	ACAGGACGCAAAAGCCATAC	NA
SNP0831	Ca_TC09339	*Ca_Desi_Chr02*	[G/T]	36520110	CDS_NON_SYNONYMOUS	FAR1	CGACTTTCAAGCGCTTTCTC	GGAGAGCAGCAAAGGTGAAG	NA
SNP0832	Ca_TC09302	*Ca_Desi_Chr03*	[C/T]	2706867	CDS_NON_SYNONYMOUS	C2C2-CO-like	TCTCTGCAATACTCACTTCACCT	GATCTTCTCGCCCCTTTTGT	NA
SNP0833	Ca_TC09302	*Ca_Desi_Chr03*	[T/C]	2709320	CDS_NON_SYNONYMOUS	C2C2-CO-like	CCATTCATCGCTCCACAAAT	TGGTCATGTTTCTCAACCTTGT	NA
SNP0834	Ca_TC14583	*Ca_Desi_Chr03*	[A/C]	3617757	CDS_NON_SYNONYMOUS	zf-HD	CCTCATGACTCATCATCACCA	ACCATTTCATCATCTCATCTCA	NA
SNP0835	Ca_TC10761	*Ca_Desi_Chr03*	[A/C]	4823888	CDS_NON_SYNONYMOUS	WRKY	CACGAAACCAAACTCCATCA	CTTTTGGGTCTGTTGATGCTC	NA
SNP0836	Ca_TC10557	*Ca_Desi_Chr03*	[C/A]	7436178	CDS_NON_SYNONYMOUS	FHA	GCGTGTTGGTGATGTCATTC	TAGTGCCACAGCATCCTCAG	NA

SNP IDs	*Kabuli* /*desi* gene accession IDs	*Kabuli* /*desi* chromosomes	SNPs	Physical positions (bp)	Sequence components of genome	Putative functions	Forward primer sequences (5'-3')	Reverse primer sequences (5'-3')	Primers used in Figure 2A
SNP0837	Ca_TC01788	*Ca_Desi_Chr03*	[A/G]	10493006	CDS_NON_SYNONYMOUS	PHD	TGTGTTTTGGGGTTTGGAAT	TCGTGCAGGCTTACTGATTG	NA
SNP0838	Ca_TC10092	*Ca_Desi_Chr03*	[G/T]	14026885	CDS_NON_SYNONYMOUS	ARF	ACAAAGGGGTGACAGCACAT	TGTTGAAGTTCCTCGTGCAG	NA
SNP0839	Ca_TC13458	*Ca_Desi_Chr03*	[G/A]	15739519	CDS_NON_SYNONYMOUS	FHA	GCTAGGGTGCAGTGAAAAGAA	AGCAACTAGAATATCCCCCTGT	NA
**SNP0840**	**Ca_TC08082**	***Ca_Desi_Chr03***	**[G/A]**	**17174233**	**CDS_NON_SYNONYMOUS**	**LUG**	**GCGAATGAAACACCCTCATT**	**TCAGAAGCTGCTGCGAGTAA**	NA
SNP0841	Ca_TC08082	*Ca_Desi_Chr03*	[C/T]	17181273	CDS_NON_SYNONYMOUS	LUG	TCCAGCTGTTCACTTTTTCTGA	CCTCCTGTTGCCTCTGTACC	NA
SNP0842	Ca_TC10875	*Ca_Desi_Chr03*	[T/C]	17936789	CDS_NON_SYNONYMOUS	MYB	TGTGGGATATGCTGTTGGAA	TTCACCTTCATAAAGATGGACGTA	NA
SNP0843	Ca_TC03750	*Ca_Desi_Chr03*	[G/A]	20174631	CDS_NON_SYNONYMOUS	S1Fa-like	CCTGGTACTTTGGCTTCAGG	AATGCCACTTCCCTTCACAG	NA
SNP0844	Ca_TC04172	*Ca_Desi_Chr03*	[T/C]	20843598	CDS_NON_SYNONYMOUS	SAP	GGCCAACTCCTAATGGTGAA	CCGTCTGAAGCAACACTCAA	NA
SNP0845	Ca_TC04619	*Ca_Desi_Chr03*	[G/A]	21776235	CDS_NON_SYNONYMOUS	GRAS	ACCCCACAGTGACACTCACA	TTGCTTCCACAAACCTTCCT	NA
SNP0846	Ca_TC25185	*Ca_Desi_Chr03*	[G/C]	22065431	CDS_NON_SYNONYMOUS	FHA	TTCTTCACGAGCTGCCTTTT	ATTCCCGCGAATTCTCTCTC	NA
SNP0847	Ca_TC00438	*Ca_Desi_Chr03*	[T/C]	23278789	CDS_NON_SYNONYMOUS	SNF2	CTGCTTTGGCAATTCAGTCA	TTAAAGAAGCCTGGGAAGCA	NA
SNP0848	Ca_TC24441	*Ca_Desi_Chr03*	[C/A]	23622995	CDS_NON_SYNONYMOUS	WRKY	GTTAGAGACCCGAAAACTAAACAAG	CGATCAAATCCTATAAAGTCTCCAC	NA
SNP0849	Ca_TC08198	*Ca_Desi_Chr03*	[G/A]	23898026	CDS_NON_SYNONYMOUS	C3H	AAGGTTAGGGCAGCCAGAAT	GGCTTGCCATTGAAACAGAT	NA
SNP0850	Ca_TC10203	*Ca_Desi_Chr03*	[G/A]	24542187	CDS_NON_SYNONYMOUS	mTERF	GCAAATTCAACCATCGAACA	AATCCAAATTCCATGCACCT	NA
SNP0851	Ca_TC10797	*Ca_Desi_Chr03*	[G/C]	25508396	CDS_NON_SYNONYMOUS	C2C2-CO-like	ATACACATTCAGGGGCAATG	GTGGAGAGGAGGGGAAGAGT	NA
SNP0852	Ca_TC14790	*Ca_Desi_Chr03*	[G/A]	25757540	CDS_NON_SYNONYMOUS	SET	TCAGCAGCAAAGAGCATCAC	CGAAAGCCGAATCTGAGAAG	NA
SNP0853	Ca_TC14504	*Ca_Desi_Chr03*	[C/T]	26544003	CDS_NON_SYNONYMOUS	NAC	GAATTTTTAAGTCCTTCATCTCTCG	AGGTCTTGATTTGCCACCAG	NA
SNP0854	Ca_TC06146	*Ca_Desi_Chr03*	[G/C]	26593875	CDS_NON_SYNONYMOUS	C2C2-GATA	CCCCTCTCTTGTTGTGTTCAA	TGGAAAGAGTAATTTTCCCCCTA	NA
SNP0855	Ca_TC05422	*Ca_Desi_Chr03*	[G/C]	28560960	CDS_NON_SYNONYMOUS	SAP	ATTGTTTATGTTGTGGCACACT	CCTTTTGTTCCAATCATATAATGC	NA
SNP0856	Ca_TC05450	*Ca_Desi_Chr03*	[G/A]	28615172	CDS_NON_SYNONYMOUS	bZIP	TGGTTAGGGTTTGCAGCTCT	GCTTCATGCAGAACGTTGAA	NA
SNP0857	Ca_TC04863	*Ca_Desi_Chr03*	[G/C]	29843867	CDS_NON_SYNONYMOUS	bZIP	CACCTTGCTGCTTGATGTGT	TCAAAGGCTAGCATGGGATT	NA
SNP0858	Ca_TC05709	*Ca_Desi_Chr03*	[T/C]	30320095	CDS_NON_SYNONYMOUS	BSD	CTGTGGAAACCATTGACGTG	CGTGTGTATGCACATGTATCTGTC	NA
SNP0859	Ca_TC07856	*Ca_Desi_Chr03*	[G/C]	30955375	CDS_NON_SYNONYMOUS	GNAT	TGCTTTGAAAGTGGGCTTCT	ATCCATCCACAAACGCAAAC	NA
SNP0860	Ca_TC01852	*Ca_Desi_Chr03*	[G/T]	31359638	CDS_NON_SYNONYMOUS	Jumonji	TCCCTTCCGAAGTATTTGATG	AGAAGCACTTGCACCCCTAA	NA
SNP0861	Ca_TC16101	*Ca_Desi_Chr03*	[A/C]	31566643	CDS_NON_SYNONYMOUS	G2-like	CGCCTCTGTCTCCATCAAAT	TGGCTTTTGGAGGTATCCTG	NA
SNP0862	Ca_TC11048	*Ca_Desi_Chr03*	[T/C]	32898333	CDS_NON_SYNONYMOUS	S1Fa-like	CACAGTTTCTCAGCGTCAGC	GCTTTCTGTCATTAGCATACCG	NA
SNP0863	Ca_TC17632	*Ca_Desi_Chr03*	[G/T]	32900447	CDS_NON_SYNONYMOUS	S1Fa-like	CCTTTTTCAAACCTCGGATTC	GAAAGGGGTCCAAGAAAAATG	NA

SNP IDs	*Kabuli*/*desi* gene accession IDs	*Kabuli*/*desi* chromosomes	SNPs	Physical positions (bp)	Sequence components of genome	Putative functions	Forward primer sequences (5'-3')	Reverse primer sequences (5'-3')	Primers used in Figure 2A
SNP0864	Ca_TC16716	*Ca_Desi_Chr03*	[T/C]	33155717	CDS_NON_SYNONYMOUS	Sigma70-like	CCCTCTACACCCTTCCCTTC	CCCTCCTTTCTTTTGGGTTC	NA
SNP0865	Ca_TC01728	*Ca_Desi_Chr03*	[G/C]	34594243	CDS_NON_SYNONYMOUS	Alfin-like	CGGATCCAATAATGGCTTTC	GAGGAAAATGGATTGCCTGA	NA
SNP0866	Ca_TC09243	*Ca_Desi_Chr03*	[T/C]	34848851	CDS_NON_SYNONYMOUS	C3H	CCAAAACTGTTTCCCATGCT	GCTCGGTGGAAATGTCAAAT	NA
SNP0867	Ca_TC03144	*Ca_Desi_Chr03*	[G/A]	35935079	CDS_NON_SYNONYMOUS	Jumonji	TCCAGGAAAAGAAGCCTCAA	TTTGTGTTGTGTGGGTTTGG	NA
SNP0868	Ca_TC11912	*Ca_Desi_Chr03*	[G/A]	36809912	CDS_NON_SYNONYMOUS	bZIP	TCAAAACCATGAATACATCAACAA	ACCCAATCTTTTCGCACAAC	NA
SNP0869	Ca_TC15928	*Ca_Desi_Chr03*	[A/T]	37102025	CDS_NON_SYNONYMOUS	SBP	ATGATGCTAGGGCTGCTGAT	TCTTTCCCGGCTGCTATATG	NA
SNP0870	Ca_TC14839	*Ca_Desi_Chr03*	[T/C]	38614598	CDS_NON_SYNONYMOUS	GeBP	ATCCACCAATTGATCGCAAC	CCTAGGAAGGGTGGTTGGTT	NA
SNP0871	Ca_TC04913	*Ca_Desi_Chr03*	[T/C]	38760068	CDS_NON_SYNONYMOUS	bZIP	AGTACCCACCCACAAAAGAA	GCCATGGATTAAAACACTCTCA	NA
SNP0872	Ca_TC00089	*Ca_Desi_Chr03*	[G/T]	38914646	CDS_NON_SYNONYMOUS	mTERF	ACATGCGGTACAGTTCACGA	TCTTTACCGCTGCTCCAGTT	NA
SNP0873	Ca_TC10811	*Ca_Desi_Chr03*	[G/C]	38969598	CDS_NON_SYNONYMOUS	MYB	CAGCGACTATTTGCTGGTCA	TTGCATCCTTCGGATTTTTC	NA
SNP0874	Ca_TC08980	*Ca_Desi_Chr03*	[G/A]	39312173	CDS_NON_SYNONYMOUS	bZIP	TCAAACCAACCAGACGAATG	GAAGTTGCACACCGACGTTA	NA
**SNP0875**	**Ca_TC17366**	***Ca_Desi_Chr03***	**[G/A]**	**39546588**	**CDS_NON_SYNONYMOUS**	**AP2-EREBP**	**TCCTGTAAGGCTGTAACTATTTGAG**	**CAACGTCCACCTCTTCTCTGC**	**Pr1**
SNP0876	Ca_TC02857	*Ca_Desi_Chr03*	[G/A]	39788772	CDS_NON_SYNONYMOUS	MYB-related	ATCAAATCATGGGGAGGTGA	TGATGAAAGGCCAACAACTG	NA
SNP0877	Ca_TC03090	*Ca_Desi_Chr03*	[T/C]	39816472	CDS_NON_SYNONYMOUS	ABI3VP1	GGAAGGAAAGGAAGGAAGGA	CCATGTTCACCCCAAATAGC	NA
SNP0878	Ca_TC03090	*Ca_Desi_Chr03*	[T/A]	39817437	CDS_NON_SYNONYMOUS	ABI3VP1	GGAAAGGAAGGAAAGGAAGG	GTTCACCCCAAATAGCCTCA	NA
SNP0879	Ca_TC05784	*Ca_Desi_Chr04*	[G/T]	19353	CDS_NON_SYNONYMOUS	bZIP	GAGCTGTGGATAGGGAATGC	ACCTTCCCACACTCGATCAC	NA
SNP0880	Ca_TC03717	*Ca_Desi_Chr04*	[A/C]	832308	CDS_NON_SYNONYMOUS	AP2-EREBP	TCTCAGGCTTGGGTAGAGGA	GGCTCTAGCAGCTTCCTTCA	NA
SNP0881	Ca_TC01002	*Ca_Desi_Chr04*	[G/T]	1047604	CDS_NON_SYNONYMOUS	SNF2	TGGTAGGAAATGGACCAAGC	TGATGACCCTATCTGCACCA	NA
SNP0882	Ca_TC14819	*Ca_Desi_Chr04*	[T/C]	1494086	CDS_NON_SYNONYMOUS	WRKY	GAGAGGCTAAAATGGAAGAGATTG	CATCAAACTTTTGGTCAATTCAA	NA
SNP0883	Ca_TC09464	*Ca_Desi_Chr04*	[T/C]	1619541	CDS_NON_SYNONYMOUS	bHLH	AAGCAGAGCAACATCAACACA	TTGCCACACCACCATTCTTA	NA
SNP0884	Ca_TC01744	*Ca_Desi_Chr04*	[T/C]	2005078	CDS_NON_SYNONYMOUS	PHD	CCCTACTACTATTGAAGAACTTGAGG	TTACAACATAGTGACATACCACCAC	NA
SNP0885	Ca_TC18030	*Ca_Desi_Chr04*	[G/T]	2168233	CDS_NON_SYNONYMOUS	SAP	TCTTCCTCTGCCCCACTATG	GAGCACCTTCCATGTCTTTCA	NA
SNP0886	Ca_TC02133	*Ca_Desi_Chr04*	[G/A]	4276290	CDS_NON_SYNONYMOUS	Jumonji	TTGAAATCGCATTCATCCAA	ATCATTTCCACCTGCACCAT	NA
SNP0887	Ca_TC04020	*Ca_Desi_Chr04*	[G/A]	4745377	CDS_NON_SYNONYMOUS	G2-like	ATGCTTCGATGATGCCTTTC	TGCTATCTCCTGGGGAACAG	NA
SNP0888	Ca_TC07000	*Ca_Desi_Chr04*	[T/C]	5004108	CDS_NON_SYNONYMOUS	bHLH	TGCTGGATAGTGGCTAGTGG	AGCGCACTCACCATTCTTTT	NA
SNP0889	Ca_TC05394	*Ca_Desi_Chr04*	[G/T]	5022053	CDS_NON_SYNONYMOUS	bZIP	AAATCCTTCTCCTCCCATCC	GCCGACGACTCCTCTAACTC	NA
SNP0890	Ca_TC04483	*Ca_Desi_Chr04*	[G/C]	5080383	CDS_NON_SYNONYMOUS	Trihelix	CAAAACCACCCTTCTCATCA	AGGCCCCACTTGAGAAGACT	NA

SNP IDs	*Kabuli /desi* gene accession IDs	*Kabuli /desi* chromosomes	SNPs	Physical positions (bp)	Sequence components of genome	Putative functions	Forward primer sequences (5'-3')	Reverse primer sequences (5'-3')	Primers used in Figure 2A
SNP0891	Ca_TC01764	*Ca_Desi_Chr04*	[T/C]	5677430	CDS_NON_SYNONYMOUS	MYB-related	TTTGGCGAACCATGGATAAT	GCAATGAGGCATCAGGATCT	NA
SNP0892	Ca_TC02989	*Ca_Desi_Chr04*	[A/T]	5747340	CDS_NON_SYNONYMOUS	Jumonji	TGGTCAATTGTGACCTTCCA	CCGAACCTATTGTCGCATTT	NA
SNP0893	Ca_TC06284	*Ca_Desi_Chr04*	[G/A]	6183406	CDS_NON_SYNONYMOUS	HB	TCCTCAGGTAGGTTTTCTTGGA	TGCTAACATTTTGATGGCAAAT	NA
SNP0894	Ca_TC07839	*Ca_Desi_Chr04*	[T/C]	6766479	CDS_NON_SYNONYMOUS	MYB	TGGGAAGACACTCTTGTTGCT	GCCAATCCCCAAGAAGAAA	NA
SNP0895	Ca_TC09768	*Ca_Desi_Chr04*	[T/C]	7394399	CDS_NON_SYNONYMOUS	AP2-EREBP	GGCGCTGGATTTCATTCTTA	TTCAAATGTTCCAAGCCACA	NA
SNP0896	Ca_TC17077	*Ca_Desi_Chr04*	[G/C]	7974685	CDS_NON_SYNONYMOUS	C3H	GGCCTACACAGGATTGGATG	GGGGGTACAGCCTTGTTCAT	NA
SNP0897	Ca_TC03473	*Ca_Desi_Chr04*	[T/C]	8390599	CDS_NON_SYNONYMOUS	BSD	CGTGGCTTAACCTTGGACAT	TGTAACAAGGCAGCATCCAG	NA
SNP0898	Ca_TC13618	*Ca_Desi_Chr04*	[G/C]	8471278	CDS_NON_SYNONYMOUS	AP2-EREBP	TGCTCATATTCGAGCCATTG	GAGCCAACACCTGTTAGAGGA	NA
SNP0899	Ca_TC08273	*Ca_Desi_Chr04*	[A/T]	8769821	CDS_NON_SYNONYMOUS	NAC	TTAGAAGTGTTTGGTCACATGC	GTACCTCATGGAAAACAAGTGG	Pr6
SNP0900	Ca_TC14853	*Ca_Desi_Chr04*	[G/A]	9471744	CDS_NON_SYNONYMOUS	AP2-EREBP	TGGAAGGAGGTAGTTGCACA	GACGGCACCATATCCATCTT	NA
SNP0901	Ca_TC18271	*Ca_Desi_Chr04*	[A/C]	10704805	CDS_NON_SYNONYMOUS	mTERF	ATGGTCCAGGACACTTCAGC	GGGGGCAGAAGAGTGATTTT	NA
SNP0902	Ca_TC14968	*Ca_Desi_Chr04*	[G/A]	10973664	CDS_NON_SYNONYMOUS	SAP	TTCCAGATTGTCTATGATCCTTCA	CTTACTTTGTTCCGCATCCA	NA
SNP0903	Ca_TC10063	*Ca_Desi_Chr04*	[G/T]	11054722	CDS_NON_SYNONYMOUS	NAC	CACAGATGAGGAGCTGGTGA	TTCTGCCTGCTCTCTTCACA	NA
SNP0904	Ca_TC12822	*Ca_Desi_Chr04*	[G/A]	11600593	CDS_NON_SYNONYMOUS	Trihelix	TGGGAATTTGTCACAAGGTG	TGGTACAAGCATGAGGGAAG	NA
SNP0905	Ca_TC03365	*Ca_Desi_Chr04*	[G/A]	12315577	CDS_NON_SYNONYMOUS	RWP-RK	TATTTTGGCACATGGTGGTG	TCCAAAATGGCCTCAGCTAC	NA
SNP0906	Ca_TC03365	*Ca_Desi_Chr04*	[G/T]	12318493	CDS_NON_SYNONYMOUS	RWP-RK	GAAAATGGCAGAATCTGAGGA	AAGTTCAACACCTGCAGCAA	NA
SNP0907	Ca_TC03365	*Ca_Desi_Chr04*	[G/C]	12319718	CDS_NON_SYNONYMOUS	RWP-RK	GAAACTTCATGGCCTTTGGA	GTGAGGCATCAGGTCTTGGT	NA
SNP0908	Ca_TC17891	*Ca_Desi_Chr04*	[G/A]	12964701	CDS_NON_SYNONYMOUS	MADS	AGAGGAAGCATGGGAAGAGG	CCACATTGTCATGCTGGAAG	NA
SNP0909	Ca_TC23817	*Ca_Desi_Chr04*	[G/A]	13328322	CDS_NON_SYNONYMOUS	Tify	CCTCCACAAGGGAATTTGAA	TGGCTGCTCCTCACTCTACA	NA
SNP0910	Ca_TC09766	*Ca_Desi_Chr04*	[T/C]	13727396	CDS_NON_SYNONYMOUS	Alfin-like	GAACGGCAACCGTATCCTTA	TGCCGAGAAACAGGGTTTAC	NA
SNP0911	Ca_TC00252	*Ca_Desi_Chr04*	[A/T]	14094264	CDS_NON_SYNONYMOUS	FHA	CAACTCCGGGATCGTAAAGA	CCAACACAGGCCCTATCACT	NA
SNP0912	Ca_TC08758	*Ca_Desi_Chr04*	[G/A]	14511914	CDS_NON_SYNONYMOUS	LIM	GAAATTGGAGCACCCATCAT	AGAGGGGGAACAAGAGAAGC	NA
SNP0913	Ca_TC04213	*Ca_Desi_Chr04*	[A/T]	15539515	CDS_NON_SYNONYMOUS	C3H	TCAAACTTATGGGGGAGGAA	ACGGTGTTGAAGTGGAGAGG	NA
SNP0914	Ca_TC34477	*Ca_Desi_Chr04*	[G/A]	16279070	CDS_NON_SYNONYMOUS	C2C2-CO-like	GCAAAGGAAATGTTGGAACTG	TCACACTCCCACTCCCTTTT	NA
SNP0915	Ca_TC16527	*Ca_Desi_Chr04*	[G/C]	16362489	CDS_NON_SYNONYMOUS	AP2-EREBP	TGTCGGAGGAATCGCTTTTA	TTCATCATCTGCACCCATGT	NA
SNP0916	Ca_TC02991	*Ca_Desi_Chr04*	[T/C]	17427472	CDS_NON_SYNONYMOUS	Jumonji	ATTACAGGCTCCCCTTTGCT	GTGCATGTCAATGGTGGAAG	NA
SNP0917	Ca_TC03801	*Ca_Desi_Chr04*	[G/T]	17722046	CDS_NON_SYNONYMOUS	SNF2	ATGAAAGGGCTTCTGGGAAT	AGACATGGCCACACATCGTA	NA

**SNP IDs**	***Kabuli*/*desi* gene accession IDs**	***Kabuli*/*desi* chromosomes**	**SNPs**	**Physical positions (bp)**	**Sequence components of genome**	**Putative functions**	**Forward primer sequences (5'-3')**	**Reverse primer sequences (5'-3')**	**Primers used in Figure 2A**
SNP0918	Ca_TC17980	*Ca_Desi_Chr04*	[G/T]	17862210	CDS_NON_SYNONYMOUS	HB	CATGAACATAAGCCCAAGCA	TGCGTGTGTATGTGTGAGAGAG	NA
SNP0919	Ca_TC07748	*Ca_Desi_Chr04*	[A/C]	19013085	CDS_NON_SYNONYMOUS	C3H	TGTTCGTTGTGTGTGCTTGA	CAATCCTCGCCTAAAAACCA	NA
SNP0920	Ca_TC07944	*Ca_Desi_Chr04*	[A/C]	19586476	CDS_NON_SYNONYMOUS	MYB-related	GGGTAACATCTAATTTCCCTCAAG	GTATCGTCTATGAGTGTGTATTGTGC	NA
SNP0921	Ca_TC16555	*Ca_Desi_Chr04*	[A/T]	20446814	CDS_NON_SYNONYMOUS	Sigma70-like	CGGGGTCAGAAAAATAACCA	GCCTCCAATACCTTGCTCAG	NA
SNP0922	Ca_TC34533	*Ca_Desi_Chr04*	[A/G]	23557644	CDS_NON_SYNONYMOUS	HB	AGGGGGTTGCTTGGTTTAAT	TTTCTTCCACCATTGGCTTC	NA
SNP0923	Ca_TC01664	*Ca_Desi_Chr04*	[C/T]	29778149	CDS_NON_SYNONYMOUS	HB	TTGTGGGCTTACGAAGAGAGA	CCCTTTTGCTTCCAATTTCA	NA
SNP0924	Ca_TC12581	*Ca_Desi_Chr04*	[G/A]	36535887	CDS_NON_SYNONYMOUS	ABI3VP1	GCCAAGATAAGCGTTTCGAC	GCCACGCCCTAGTTTTCATA	NA
SNP0925	Ca_TC02171	*Ca_Desi_Chr04*	[G/A]	36974817	CDS_NON_SYNONYMOUS	SNF2	AAAACGGTTCAGGCAATCAC	CTTCGCTGACCTCTTCCATC	NA
SNP0926	Ca_TC03468	*Ca_Desi_Chr04*	[T/C]	37061273	CDS_NON_SYNONYMOUS	SET	ACATTTTCGCGACCTTGTTC	GCTTGCCAGTCAACTCATCA	NA
SNP0927	Ca_TC00942	*Ca_Desi_Chr04*	[T/C]	37096147	CDS_NON_SYNONYMOUS	ABI3VP1	ACAATAAACGGAGGCGAAGA	TCTCCCCCTTGTGTGCTTAC	NA
SNP0928	Ca_TC00942	*Ca_Desi_Chr04*	[A/T]	37097065	CDS_NON_SYNONYMOUS	ABI3VP1	GTTGATATTGTCAAGCATATAG	TCCTTCTCGAGAATCAGATTCA	NA
SNP0929	Ca_TC00942	*Ca_Desi_Chr04*	[A/T]	37097068	CDS_NON_SYNONYMOUS	ABI3VP1	TTGTCAAGCATATAGTCTATAGTC	GCTTACAAGTTACAGCCTTC	NA
SNP0930	Ca_TC11159	*Ca_Desi_Chr04*	[G/A]	37514472	CDS_NON_SYNONYMOUS	Tify	AACTTGCTTTGAATTCTCCACTG	TGGGTTTCAATTCAAGGTGA	NA
SNP0931	Ca_TC18630	*Ca_Desi_Chr04*	[A/T]	38486601	CDS_NON_SYNONYMOUS	MADS	CAGTGTGCAAAGGAAACCAA	CCTCACAAAGCTTGCAGTTTC	NA
SNP0932	Ca_TC05974	*Ca_Desi_Chr04*	[G/C]	38659802	CDS_NON_SYNONYMOUS	GRAS	TTGAACACGAAGCAGAAGTTGATTG	TAGGCAACCCCACAAACCCAAGCAATTC	NA
SNP0933	Ca_TC04825	*Ca_Desi_Chr04*	[G/A]	39804794	CDS_NON_SYNONYMOUS	Trihelix	CGCCCATGGAATCTACATCT	GGAGATTTCCATGCAGAAGG	NA
SNP0934	Ca_TC15768	*Ca_Desi_Chr04*	[G/A]	40230993	CDS_NON_SYNONYMOUS	Sigma70-like	TGGGTCTCTGTTTCCGTTTT	TAAGGCCAAAGCGTAGGATG	NA
SNP0935	Ca_TC00983	*Ca_Desi_Chr04*	[A/T]	40578365	CDS_NON_SYNONYMOUS	Jumonji	ATCGATGAAAAGGCGAAAGA	TGGAAGAGGCAGAGCAATCT	NA
SNP0936	Ca_TC18510	*Ca_Desi_Chr04*	[T/A]	40933076	CDS_NON_SYNONYMOUS	bZIP	CAGCATCGCTATTGACTCCA	ATTCTCGCGCCTTCTTCAAT	NA
SNP0937	Ca_TC04841	*Ca_Desi_Chr04*	[G/C]	41866592	CDS_NON_SYNONYMOUS	LUG	TTTCATGCAGACTCCACAGC	ATTCCCCTTCACTCCCAGAT	NA
SNP0938	Ca_TC23107	*Ca_Desi_Chr04*	[T/C]	42354903	CDS_NON_SYNONYMOUS	bZIP	CCGGAATCCATTAACAAACG	CGGTGAATTTGTTGCAGTGT	NA
SNP0939	Ca_TC01983	*Ca_Desi_Chr04*	[G/A]	43891050	CDS_NON_SYNONYMOUS	HB	CATGAGCAAGCCTTCAATCA	CTCCAAACGTCTTCCGACTC	NA
SNP0940	Ca_TC14573	*Ca_Desi_Chr04*	[G/C]	44393302	CDS_NON_SYNONYMOUS	C2C2-CO-like	GCAATGCAATGCAATTATCAG	CTTTTCTCATTCATTCAAACAAAA	NA
SNP0941	Ca_TC14573	*Ca_Desi_Chr04*	[C/T]	44394420	CDS_NON_SYNONYMOUS	C2C2-CO-like	TTAGAAAAAGACAGAGGAAAAAGAA	CTTTTCTCATTCATTCAAACAAAA	NA
SNP0942	Ca_TC07900	*Ca_Desi_Chr04*	[T/C]	45416533	CDS_NON_SYNONYMOUS	HB	AGGGACCATCAGGCATGTAG	CTCCACCCTGTCAAGACCAT	NA
SNP0943	Ca_TC18421	*Ca_Desi_Chr04*	[T/C]	45736499	CDS_NON_SYNONYMOUS	AP2-EREBP	TTGAAGAAAAACATTGACAGAAGC	GGAGGAGGATAGCCACTGGT	NA
SNP0944	Ca_TC18961	*Ca_Desi_Chr04*	[T/C]	45835578	CDS_NON_SYNONYMOUS	LIM	TTGATCAAAATGATTGTCACG	TCCACACACTTGCAAACAAA	NA

SNP IDs	*Kabuli*/*desi* gene accession IDs	*Kabuli*/*desi* chromosomes	SNPs	Physical positions (bp)	Sequence components of genome	Putative functions	Forward primer sequences (5'-3')	Reverse primer sequences (5'-3')	Primers used in Figure 2A
SNP0945	Ca_TC10605	*Ca_Desi_Chr04*	[G/C]	46419887	CDS_NON_SYNONYMOUS	HB	AAGGTGCATCAGTTCACACG	AAACCCCTTCATCTGCCTTC	NA
SNP0946	Ca_TC17705	*Ca_Desi_Chr04*	[T/C]	46995447	CDS_NON_SYNONYMOUS	CCAAT	ACACTTGTATTCAGTTTCATCCAAA	TGAAAATTCAGATTAACAATTAACAAC	NA
SNP0947	Ca_TC16471	*Ca_Desi_Chr04*	[A/T]	47258388	CDS_NON_SYNONYMOUS	ABI3VP1	TTTTCTGCACGTGATGATGG	CCAACCTCGAATCTTCCTCA	NA
SNP0948	Ca_TC06172	*Ca_Desi_Chr04*	[G/A]	47445593	CDS_NON_SYNONYMOUS	LIM	CATCGACTTCAGGGGATGTT	AATCAGCACTGCATCAAAGC	NA
SNP0949	Ca_TC15885	*Ca_Desi_Chr04*	[G/A]	47884183	CDS_NON_SYNONYMOUS	HMG	CAGTGCCCTCTTAGGTGCAT	TGGCGTTGAGATCAAAGATG	NA
SNP0950	Ca_TC09314	*Ca_Desi_Chr04*	[G/A]	48124866	CDS_NON_SYNONYMOUS	HB	ATTTGCCACCTGGTTTCTTG	CTTGGGATTGCCACTTGTCT	NA
SNP0951	Ca_TC00756	*Ca_Desi_Chr04*	[G/T]	48276063	CDS_NON_SYNONYMOUS	SWI/SNF-SWI3	AATGTTTCCGTTGAGGGTCA	AGCTTTCAGCCCAGCAGATA	NA
**SNP0952**	**Ca_TC03708**	***Ca_Desi_Chr04***	**[C/T]**	**48384848**	**CDS_NON_SYNONYMOUS**	**ARF**	**GTATCAACTTTGCCATTTTGTG**	**CTTGCAAGGGAAGTCTTATAG**	**Pr2**
SNP0953	Ca_TC15083	*Ca_Desi_Chr05*	[A/G]	166360	CDS_NON_SYNONYMOUS	NAC	GCAATAGCAGCAGCAACAAC	GAGGAATAATGAGAGTGACAAGC	NA
SNP0954	Ca_TC21593	*Ca_Desi_Chr05*	[G/A]	7382449	CDS_NON_SYNONYMOUS	bHLH	CGCAATGAACGACAGTGTTT	TGAACTCCTTGGGGACCATA	NA
SNP0955	Ca_TC19883	*Ca_Desi_Chr05*	[G/A]	7681689	CDS_NON_SYNONYMOUS	MADS	TGGGATTCCAAATTTTTCCA	TTTTGGTCACTTGGGCTAATG	NA
SNP0956	Ca_TC10166	*Ca_Desi_Chr05*	[G/A]	9958884	CDS_NON_SYNONYMOUS	WRKY	GGTCCAAGAGGCATGTGATT	TCCATCTTCAGGCCAACTTC	NA
SNP0957	Ca_TC24111	*Ca_Desi_Chr05*	[T/C]	10989680	CDS_NON_SYNONYMOUS	FAR1	CATGCACCCTATGAACAACG	TGAATTCCCCCACAGTTGAT	NA
SNP0958	Ca_TC00525	*Ca_Desi_Chr05*	[G/A]	11384336	CDS_NON_SYNONYMOUS	C3H	TTCCCATAAAAACGCACACA	AGCCAAACATACGCTTCTGC	NA
SNP0959	Ca_TC00942	*Ca_Desi_Chr05*	[A/C]	14755276	CDS_NON_SYNONYMOUS	ABI3VP1	AGCATATAGTCTATAGTCTATA	TCACGACAAACGCGCGGTCGC	NA
SNP0960	Ca_TC15918	*Ca_Desi_Chr05*	[G/A]	16677409	CDS_NON_SYNONYMOUS	C3H	ATAATGATGGGGCCAGAGATAATAG	TCATACCAAATGTACTCAGAGACTGC	NA
SNP0961	Ca_TC18783	*Ca_Desi_Chr05*	[T/C]	19048521	CDS_NON_SYNONYMOUS	GNAT	GGTTCATAATTCGGCACCTC	TTTTGACATGAACCGCAAAC	NA
SNP0962	Ca_TC10952	*Ca_Desi_Chr05*	[G/T]	21801207	CDS_NON_SYNONYMOUS	FHA	TGAGCAGGATAGAGCGGATT	CATATCTCCTCCGCCAAGAG	NA
SNP0963	Ca_TC10952	*Ca_Desi_Chr05*	[C/G]	21801378	CDS_NON_SYNONYMOUS	FHA	CAGTCGGCAAGATTGTATTGT	CAGGACCACTCTCACAACCA	NA
SNP0964	Ca_TC16202	*Ca_Desi_Chr05*	[A/T]	24343946	CDS_NON_SYNONYMOUS	CCAAT	CCCATCAAACAAAGAGAGTTGG	GGAGAGGAATCGGATTAGCC	NA
SNP0965	Ca_TC15539	*Ca_Desi_Chr05*	[T/C]	24717637	CDS_NON_SYNONYMOUS	MYB	TTTTCCCTTTTGTTGTGTCTCA	CAACCAATCACCACCACATC	NA
SNP0966	Ca_TC16449	*Ca_Desi_Chr05*	[A/G]	26426790	CDS_NON_SYNONYMOUS	WRKY	ATCCTAAGCCAGCCCCTCTA	TGATCAAACAGGGTCAGGTG	NA
SNP0967	Ca_TC01938	*Ca_Desi_Chr05*	[G/A]	27042239	CDS_NON_SYNONYMOUS	MYB-related	CGCATTCTCCAAACTCAACA	TTACCAATTGGCCCTCACTC	NA
SNP0968	Ca_TC02970	*Ca_Desi_Chr05*	[G/C]	27150196	CDS_NON_SYNONYMOUS	ABI3VP1	ACGATCTGGTGGATTCGTTC	TGTTTGTCATGTCGCCATTT	NA
SNP0969	Ca_TC02631	*Ca_Desi_Chr05*	[T/C]	27749046	CDS_NON_SYNONYMOUS	ARF	GTCGCTAAGCCTTTGTCCTG	AACTCTCATTGGCGCTCTGT	NA
SNP0970	Ca_TC10514	*Ca_Desi_Chr05*	[T/C]	28253736	CDS_NON_SYNONYMOUS	LOB	TGAAATGAAAGGCATGAAAGG	CATTTTCCAAGAGTTCCTAGCTG	NA
SNP0971	Ca_TC16262	*Ca_Desi_Chr05*	[G/C]	29397487	CDS_NON_SYNONYMOUS	AP2-EREBP	CTTCTGTAACACCCCGCTTC	CATTGCAGTAGCCACCAAAC	NA

**SNP IDs**	***Kabuli*/*desi* gene accession IDs**	***Kabuli*/*desi* chromosomes**	**SNPs**	**Physical positions (bp)**	**Sequence components of genome**	**Putative functions**	**Forward primer sequences (5'-3')**	**Reverse primer sequences (5'-3')**	**Primers used in Figure 2A**
SNP0972	Ca_TC06871	*Ca_Desi_Chr05*	[T/C]	29790311	CDS_NON_SYNONYMOUS	FHA	TCCATTAGATGGACCCCTTG	TCAGAAGCAAAACCCAAACC	NA
SNP0973	Ca_TC04443	*Ca_Desi_Chr05*	[C/G]	30439023	CDS_NON_SYNONYMOUS	MYB-related	TGAGAAGCTGAAAGGGAAGC	CAATGAAAAGCTACCTGCACA	NA
SNP0974	Ca_TC03920	*Ca_Desi_Chr05*	[A/T]	31244017	CDS_NON_SYNONYMOUS	Bromodomain	CGATGAACGCAAACTCAAGA	CGTTTGAAGACCCGGTTAAG	NA
SNP0975	Ca_TC18361	*Ca_Desi_Chr05*	[G/A]	33879428	CDS_NON_SYNONYMOUS	bZIP	CCCTTTCTCCTTTCCTCTCC	CACGGCATATAAAGTTGGATCA	NA
SNP0976	Ca_TC14720	*Ca_Desi_Chr05*	[G/A]	34476592	CDS_NON_SYNONYMOUS	Tify	TGACATAAGCATTTCAGCCTCA	CGCCCATTAATATCAACACAAA	NA
SNP0977	Ca_TC05162	*Ca_Desi_Chr05*	[T/C]	34510155	CDS_NON_SYNONYMOUS	LUG	ACAGCAACAGCAACAACAGC	GAAATGGCAACAGGCAACTT	NA
SNP0978	Ca_TC12011	*Ca_Desi_Chr05*	[A/T]	34684944	CDS_NON_SYNONYMOUS	bZIP	TGGTACATTAACTACCCTTTTATCTCC	AACATGGTAGCGGGAACAAC	NA
SNP0979	Ca_TC15633	*Ca_Desi_Chr05*	[G/A]	35117329	CDS_NON_SYNONYMOUS	ARR-B	ACCAGCAACCGAGACAAAAC	GTGCCAAATGCCCAACTATC	NA
SNP0980	Ca_TC12603	*Ca_Desi_Chr05*	[T/C]	35122407	CDS_NON_SYNONYMOUS	PHD	AAACTGGGAGCGTGAATTTG	CAGCCTCAGCTTCATCAACA	NA
SNP0981	Ca_TC06000	*Ca_Desi_Chr05*	[A/T]	35389971	CDS_NON_SYNONYMOUS	C2C2-CO-like	GCCGTATCCAGCAACAGAAT	CCTGTCCCACCACACTAGGTA	NA
SNP0982	Ca_TC17175	*Ca_Desi_Chr05*	[A/T]	36046998	CDS_NON_SYNONYMOUS	HB	TGGTCTAGGCCTCCATGAAC	GCAAAAGATTGGGTGAACGA	NA
SNP0983	Ca_TC02754	*Ca_Desi_Chr05*	[T/C]	36211837	CDS_NON_SYNONYMOUS	BSD	GTCAACATGGCTTGCTCGT	GGCAAAAGAATGTTGGGAGA	NA
SNP0984	Ca_TC06070	*Ca_Desi_Chr05*	[A/T]	36283170	CDS_NON_SYNONYMOUS	LIM	GGTGAAAGATATTCGAGGGCTA	TGAGACTCAAACACCCCTAACA	NA
SNP0985	Ca_TC06893	*Ca_Desi_Chr05*	[T/C]	37147512	CDS_NON_SYNONYMOUS	zf-HD	CCATCCTCAAAATTTCTCATGC	GCATCCAAACCTTAAGCACA	NA
SNP0986	Ca_TC15434	*Ca_Desi_Chr05*	[A/C]	37485160	CDS_NON_SYNONYMOUS	C2C2-GATA	ACATACCTCGCTCGCCTTC	CCAGCTTTCTTTAAATGGGAGA	NA
SNP0987	Ca_TC03890	*Ca_Desi_Chr05*	[G/A]	37814550	CDS_NON_SYNONYMOUS	Jumonji	AACAGTTTCTTGCCCCACAC	CCGGTCATGCTTAATGGACT	NA
SNP0988	Ca_TC11222	*Ca_Desi_Chr05*	[T/C]	37985131	CDS_NON_SYNONYMOUS	MYB-related	CGATTTTGCGCAAGGATAAT	AATTTCAAGAACGCCGAAGA	NA
SNP0989	Ca_TC12007	*Ca_Desi_Chr05*	[T/C]	38149806	CDS_NON_SYNONYMOUS	bHLH	AACTGCCACCACAAGAATCG	TGAGTCCTTAGCACCACAACTG	NA
SNP0990	Ca_TC15723	*Ca_Desi_Chr05*	[G/A]	38467232	CDS_NON_SYNONYMOUS	GNAT	CGTGTACGAGCCATAGCAGA	TGTGTTCTTTGCTGGCAATC	NA
SNP0991	Ca_TC11405	*Ca_Desi_Chr05*	[T/C]	40243059	CDS_NON_SYNONYMOUS	FAR1	TTTTCCTGGCCTTCTCTCAA	TGAGGATGGGATTTCTGAGC	NA
SNP0992	Ca_TC06049	*Ca_Desi_Chr05*	[T/C]	40405468	CDS_NON_SYNONYMOUS	FHA	TGAGGGACCAGAAAGTTTGATCTT	CAAATGGAACTCCGCAAGACCTGGCA	NA
SNP0993	Ca_TC10289	*Ca_Desi_Chr05*	[A/C]	40619677	CDS_NON_SYNONYMOUS	HB	AAATTTTGCTCCTCCATTCTCA	GTGTGTGGGTGTTGAAACCA	NA
SNP0994	Ca_TC11566	*Ca_Desi_Chr05*	[T/C]	41313407	CDS_NON_SYNONYMOUS	SET	AATGAATGGCCTCATTTGAA	TGTTCACCAACACCACCAAT	NA
SNP0995	Ca_TC10983	*Ca_Desi_Chr05*	[T/C]	41478089	CDS_NON_SYNONYMOUS	SWI/SNF-SWI3	ATTGAAGAACACCGGCTTGT	CACAGCCTCTCATGCACATT	NA
SNP0996	Ca_TC01272	*Ca_Desi_Chr05*	[C/T]	42527654	CDS_NON_SYNONYMOUS	CAMTA	AACCTGCCCCTTCATCTTTT	ATGAACGGCTTAAGGTTGGA	NA
SNP0997	Ca_TC04623	*Ca_Desi_Chr05*	[A/C]	42984383	CDS_NON_SYNONYMOUS	ARR-B	TGCTTCACTTCCCCTAGACG	AGAAGTGGAAATGGGCTGTG	NA
SNP0998	Ca_TC12604	*Ca_Desi_Chr05*	[G/C]	43835527	CDS_NON_SYNONYMOUS	bZIP	TTCCCCATTTCCTCTTCCTT	CACTCCTGGCCAATGAAAAT	NA

**SNP IDs**	***Kabuli*/*desi* gene accession IDs**	***Kabuli*/*desi* chromosomes**	**SNPs**	**Physical positions (bp)**	**Sequence components of genome**	**Putative functions**	**Forward primer sequences (5'-3')**	**Reverse primer sequences (5'-3')**	**Primers used in Figure 2A**
SNP0999	Ca_TC01857	*Ca_Desi_Chr05*	[G/T]	44421018	CDS_NON_SYNONYMOUS	SET	GCAGGCTGATCAATTTCCAT	TTCCCTGTTGCACAGTTCAG	NA
SNP1000	Ca_TC02822	*Ca_Desi_Chr05*	[A/T]	44560496	CDS_NON_SYNONYMOUS	HB	TCACATGGGTTCCTGGTACA	CACCAGAAGTGGTCCCAAAT	NA
SNP1001	Ca_TC10914	*Ca_Desi_Chr05*	[G/C]	45076994	CDS_NON_SYNONYMOUS	Sigma70-like	GCGACGAAGATAATCCAACC	GCTGCTCGAACTCCACTAGG	NA
SNP1002	Ca_TC01071	*Ca_Desi_Chr05*	[A/C]	46175167	CDS_NON_SYNONYMOUS	NAC	TCCGATTCCGACCTACAGAC	AGCACGACTTTCTGCAGCTT	NA
SNP1003	Ca_TC11233	*Ca_Desi_Chr05*	[T/C]	46513987	CDS_NON_SYNONYMOUS	bZIP	GAGGAGACAACCGCAAGAAG	CAATGTCCAACCCCTGAAAC	NA
SNP1004	Ca_TC07991	*Ca_Desi_Chr05*	[G/A]	47223799	CDS_NON_SYNONYMOUS	C3H	GAGGAACGGAAACAGCAGAG	CCTGCTCCAAGGATATGAGC	NA
SNP1005	Ca_TC04499	*Ca_Desi_Chr05*	[A/G]	47734066	CDS_NON_SYNONYMOUS	NAC	GCAAAACACAACAACAACAAAAA	TGCATTCCATCTCTTTGAATTG	NA
SNP1006	Ca_TC10067	*Ca_Desi_Chr06*	[C/G]	789313	CDS_NON_SYNONYMOUS	GRAS	GACCTCCACTGGCTCAATGT	GCGGAATGGACGAGTTTTTA	NA
SNP1007	Ca_TC03126	*Ca_Desi_Chr06*	[A/C]	1609229	CDS_NON_SYNONYMOUS	LIM	GTGGTGGTCATCATTTGCAG	GAACACACCTTCCCCTCAGA	NA
SNP1008	Ca_TC01051	*Ca_Desi_Chr06*	[G/A]	1842423	CDS_NON_SYNONYMOUS	GeBP	AGCCACCACAAGCAACTTCT	CAATTCTGCATTGCTTGCAT	NA
SNP1009	Ca_TC04335	*Ca_Desi_Chr06*	[G/A]	2925948	CDS_NON_SYNONYMOUS	Tify	TCCATGTAACACAAAAGCCCTA	CACTCTCTTCCTTTTCCTTTGG	NA
SNP1010	Ca_TC08424	*Ca_Desi_Chr06*	[G/A]	5506963	CDS_NON_SYNONYMOUS	bHLH	CAAGATTGGCCAAGGGTTAC	CTTTTTGGAGGGCTTGTTTG	NA
SNP1011	Ca_TC13769	*Ca_Desi_Chr06*	[T/C]	5688576	CDS_NON_SYNONYMOUS	LIM	TGCAAGAAAAACCTCCCAAA	AACCCAACAGAAATGCAAGG	NA
SNP1012	Ca_TC05745	*Ca_Desi_Chr06*	[G/A]	5789222	CDS_NON_SYNONYMOUS	SBP	CGGTGGAGGAACATTCATGCGCGGA	CTGGATGCAGACCCACTAGACTAA	Pr7
SNP1013	Ca_TC26643	*Ca_Desi_Chr06*	[T/C]	6015415	CDS_NON_SYNONYMOUS	NAC	TGGGATTTACCAGGAGGTTG	TTGATGATCTGGTGCATTTGA	NA
SNP1014	Ca_TC16024	*Ca_Desi_Chr06*	[G/A]	6142063	CDS_NON_SYNONYMOUS	ARF	ACTATGGCATGCTTGTGCTG	AGACCAGGTGGCATAACAGG	NA
SNP1015	Ca_TC09243	*Ca_Desi_Chr06*	[G/A]	6295375	CDS_NON_SYNONYMOUS	C3H	CAAAACTGTTTCCCATGCT	TCGGTGGAAATGTCAAAT	NA
SNP1016	Ca_TC23737	*Ca_Desi_Chr06*	[T/C]	6913579	CDS_NON_SYNONYMOUS	SNF2	CGTCAACAATTCGCTCATGT	AAAGGGGTCCCGATATTTTG	NA
SNP1017	Ca_TC09845	*Ca_Desi_Chr06*	[A/T]	7275013	CDS_NON_SYNONYMOUS	MYB	TTAGACGAAGACGCGAAACC	CGCACGAGTTACGTGTTCAT	NA
SNP1018	Ca_TC02597	*Ca_Desi_Chr06*	[A/C]	10032665	CDS_NON_SYNONYMOUS	zf-HD	TGCCATGGCTCTCACTACAG	TGAGGTGCTGATGGGTATGA	NA
SNP1019	Ca_TC00090	*Ca_Desi_Chr06*	[C/T]	10748091	CDS_NON_SYNONYMOUS	mTERF	ACGTTTTCGCCACCTTACAC	AAACACTCGATCCTCGATGC	NA
SNP1020	Ca_TC05646	*Ca_Desi_Chr06*	[G/A]	10864322	CDS_NON_SYNONYMOUS	CAMTA	AGACCATGATTTCCCTGACG	TCGTTTGCTCTCCGTTTTCT	NA
SNP1021	Ca_TC15791	*Ca_Desi_Chr06*	[G/C]	13242976	CDS_NON_SYNONYMOUS	C3H	AACAGCCGCAACTTCAGATT	CAGAAAACCCGCTTCTGTCT	NA
SNP1022	Ca_TC16835	*Ca_Desi_Chr06*	[G/A]	13996302	CDS_NON_SYNONYMOUS	HMG	CCGAGTTGCAAGAGAAAAGAG	AAGCCCACAGCTGAAATTCT	NA
SNP1023	Ca_TC11344	*Ca_Desi_Chr06*	[G/A]	14815110	CDS_NON_SYNONYMOUS	CAMTA	ACCAAGAGCAGCAACCAAAT	TTCATGCTTTTCATGGGTCA	NA
SNP1024	Ca_TC10606	*Ca_Desi_Chr06*	[C/T]	15273685	CDS_NON_SYNONYMOUS	Bromodomain	CGTCTTGCATCGGAACTTTT	CAATTAGCCACTCCCACGTT	NA
SNP1025	Ca_TC02723	*Ca_Desi_Chr06*	[T/C]	15848050	CDS_NON_SYNONYMOUS	Jumonji	ATCTGCATGCTCCTTCCAGT	TGTGCTGACAATTTGGTGGT	NA

SNP IDs	*Kabuli* /*desi* gene accession IDs	*Kabuli* /*desi* chromosomes	SNPs	Physical positions (bp)	Sequence components of genome	Putative functions	Forward primer sequences (5'-3')	Reverse primer sequences (5'-3')	Primers used in Figure 2A
SNP1026	Ca_TC16337	*Ca_Desi_Chr06*	[A/C]	18443828	CDS_NON_SYNONYMOUS	bZIP	GGCAAGCGAGTCTCTCCTTA	AATGCCTTCCTTCCTACAAGC	NA
SNP1027	Ca_TC10915	*Ca_Desi_Chr06*	[A/T]	18949559	CDS_NON_SYNONYMOUS	mTERF	GGGTTGCAAGGGAGACAAT	TTGTATCCAAATGCCCCAAA	NA
SNP1028	Ca_TC15258	*Ca_Desi_Chr06*	[C/T]	20519235	CDS_NON_SYNONYMOUS	NAC	TCCCTTCTCATGTGTTGTCA	TTGTCAACAAGGCATCGAAA	NA
SNP1029	Ca_TC08439	*Ca_Desi_Chr06*	[A/T]	21437289	CDS_NON_SYNONYMOUS	Bromodomain	AAGCAATTGGAGGAGCAAAA	CTGCAAATTCCTTGGGTGAT	NA
SNP1030	Ca_TC03453	*Ca_Desi_Chr06*	[G/A]	21497337	CDS_NON_SYNONYMOUS	bZIP	CTCCCTCCTTACATGTATTTATAA	AACTTTGAGTGGTCCCCTTG	NA
SNP1031	Ca_TC00789	*Ca_Desi_Chr06*	[G/A]	21675697	CDS_NON_SYNONYMOUS	HB	GAGATGGAGAGGCAAGAACG	TTGACGCTAAACCCTCATCC	NA
SNP1032	Ca_TC08915	*Ca_Desi_Chr06*	[G/C]	21716470	CDS_NON_SYNONYMOUS	G2-like	GCACATGAAGAGGGTGGAAT	TGTTGCCAGATTTGGAGATG	NA
SNP1033	Ca_TC07431	*Ca_Desi_Chr06*	[T/C]	21777225	CDS_NON_SYNONYMOUS	AP2-EREBP	CTCTCTTGCGAAACACACCA	ACATCCATTGGTGGTGCTAA	NA
SNP1034	Ca_TC00539	*Ca_Desi_Chr06*	[T/C]	22228859	CDS_NON_SYNONYMOUS	E2F-DP	TTTAGGAGCGAAACGAAACG	TGGGCTAGTACAACCGAGTTC	NA
SNP1035	Ca_TC02346	*Ca_Desi_Chr06*	[G/A]	22711105	CDS_NON_SYNONYMOUS	ARF	TGCCTTCAGTTTCATTGCAG	GTACCGTCTCCACCGTTCAT	NA
SNP1036	Ca_TC18399	*Ca_Desi_Chr06*	[T/C]	23284891	CDS_NON_SYNONYMOUS	NAC	AAAAACCCAGTAACACACTGCAT	AGTCCAGTTTTGCAGCCAAG	NA
SNP1037	Ca_TC11438	*Ca_Desi_Chr06*	[A/G]	24586525	CDS_NON_SYNONYMOUS	bZIP	ATGATAGCGCAGCAGAGGTC	CGGTTTGGTAGTTTCGGTGT	NA
SNP1038	Ca_TC06796	*Ca_Desi_Chr06*	[A/T]	26357644	CDS_NON_SYNONYMOUS	GRAS	CAGGTCACAAGTTTCCGTGA	ACAGATTGTGCACACCGAAC	NA
SNP1039	Ca_TC02132	*Ca_Desi_Chr06*	[G/T]	27861705	CDS_NON_SYNONYMOUS	C3H	TCATCCCTCTCAGGATCACC	TCAGGATTCAACCCTCGAAC	NA
SNP1040	Ca_TC08004	*Ca_Desi_Chr06*	[G/A]	28103045	CDS_NON_SYNONYMOUS	HB	CTTGATTCGACCGTGTGATG	TGCAATCGCTTGTTCGTAAG	NA
SNP1041	Ca_TC27287	*Ca_Desi_Chr06*	[A/C]	30942481	CDS_NON_SYNONYMOUS	bZIP	TTGATTGGCAGTATCATCGAAC	TGGTAAGTCACATAATCCACTAGACC	NA
SNP1042	Ca_TC12166	*Ca_Desi_Chr06*	[T/C]	31477570	CDS_NON_SYNONYMOUS	E2F-DP	ATGCAATCCATGAAACGACA	AGGACAAGCTTCGCAGAGAG	NA
SNP1043	Ca_TC12166	*Ca_Desi_Chr06*	[G/T]	31478512	CDS_NON_SYNONYMOUS	E2F-DP	CGCTTTTTGTTTTCCGACTT	AACCATCAGGAGGGTTGGAT	NA
SNP1044	Ca_TC06924	*Ca_Desi_Chr06*	[T/C]	32082602	CDS_NON_SYNONYMOUS	C2C2-YABBY	ATGGACATGATGGCAACAGA	TTGTCTCCAATCTTTGACTAAGGA	NA
SNP1045	Ca_TC17223	*Ca_Desi_Chr06*	[G/A]	32326867	CDS_NON_SYNONYMOUS	WRKY	TGTTTTGTTGAACACCACCAA	GGATCTTGGATTGGCTGCTA	NA
SNP1046	Ca_TC02231	*Ca_Desi_Chr06*	[G/T]	37388155	CDS_NON_SYNONYMOUS	OFP	TTGCAAGACTACCGGAGAGG	TGATTCCGAATGCTTGGTTT	NA
SNP1047	Ca_TC15677	*Ca_Desi_Chr06*	[G/A]	45709378	CDS_NON_SYNONYMOUS	WRKY	ATTCTTCCGATGTTCGTTGG	TTTCTTGAATCCCCAGTGCT	NA
SNP1048	Ca_TC12430	*Ca_Desi_Chr06*	[T/A]	45713690	CDS_NON_SYNONYMOUS	GRAS	TCAACACAACGACAACCTTTG	CACGTGGTGAAGCATAATGG	NA
SNP1049	Ca_TC06153	*Ca_Desi_Chr06*	[T/A]	53173286	CDS_NON_SYNONYMOUS	WRKY	TTGGGGATGACCAAGTTTTC	AGTCGGCTTTATGGTGCAGT	NA
SNP1050	Ca_TC19359	*Ca_Desi_Chr06*	[T/C]	53654154	CDS_NON_SYNONYMOUS	bHLH	AAAGAACAAGAAATTTGTCAAGAACA	TCACCACGGAAAAAGTAGGC	NA
SNP1051	Ca_TC10558	*Ca_Desi_Chr06*	[G/A]	54359260	CDS_NON_SYNONYMOUS	GRAS	CTGCTCCCTCACAAGCTACC	TGAGACTCCAAGCTGCTGAA	NA
SNP1052	Ca_TC04801	*Ca_Desi_Chr06*	[G/A]	55333022	CDS_NON_SYNONYMOUS	HB	GATGCGTGGAGAAGGAAAAA	CAGCTAAACCAATGCCATGA	NA

SNP IDs	*Kabuli* /*desi* gene accession IDs	*Kabuli* /*desi* chromosomes	SNPs	Physical positions (bp)	Sequence components of genome	Putative functions	Forward primer sequences (5'-3')	Reverse primer sequences (5'-3')	Primers used in Figure 2A
SNP1053	Ca_TC11343	*Ca_Desi_Chr06*	[C/A]	55637657	CDS_NON_SYNONYMOUS	ULT	TGAGTCAGCCCATAGGAACA	ATTCATTCAACCCCATCACC	NA
SNP1054	Ca_TC18101	*Ca_Desi_Chr06*	[T/C]	57024461	CDS_NON_SYNONYMOUS	MADS	TCTTTCACAAATTTGGTACTGACC	TGCAGCCAAAGAAAAACTGA	NA
SNP1055	Ca_TC14643	*Ca_Desi_Chr06*	[G/A]	57532005	CDS_NON_SYNONYMOUS	FAR1	CGTCATCATCTCCATTGTGG	TGAGACCGATCCATCACAAA	NA
SNP1056	Ca_TC04361	*Ca_Desi_Chr06*	[T/C]	57720386	CDS_NON_SYNONYMOUS	ABI3VP1	GGGACATCCATCAAATGCTT	GCAAACCGACAACATGCTAA	NA
SNP1057	Ca_TC20893	*Ca_Desi_Chr06*	[G/A]	58112367	CDS_NON_SYNONYMOUS	MYB-related	CTTGAGGATTGCCAGAAAGC	CTGAGTCTGGGCTTGACGAT	NA
SNP1058	Ca_TC16566	*Ca_Desi_Chr06*	[A/T]	58242882	CDS_NON_SYNONYMOUS	mTERF	AGCGAAAACCACACTTCACC	GGATGATTCGACCCTCAAGA	NA
SNP1059	Ca_TC07261	*Ca_Desi_Chr06*	[G/T]	58555767	CDS_NON_SYNONYMOUS	bZIP	CGCAGGACCCATTTAGTTCA	CCACCACCTAACAACCCTTG	NA
SNP1060	Ca_TC10295	*Ca_Desi_Chr06*	[C/A]	58810441	CDS_NON_SYNONYMOUS	C2C2-GATA	GACCGGTTCGTTGACAAAAT	CCCCTCTTTGTTCTTCACCA	NA
SNP1061	Ca_TC17073	*Ca_Desi_Chr07*	[T/C]	82458	CDS_NON_SYNONYMOUS	CCAAT	GGTGGACAAGGTTCTTCAGG	GGCCATGATTAAAAACATGAAA	NA
SNP1062	Ca_TC10725	*Ca_Desi_Chr07*	[A/C]	217223	CDS_NON_SYNONYMOUS	GRAS	TCCAGATGCAAACGTCTCAA	AACGCGCATTGTCTAAATCC	NA
SNP1063	Ca_TC05104	*Ca_Desi_Chr07*	[G/A]	888424	CDS_NON_SYNONYMOUS	WRKY	AGGGCTGAGTGATGGTTGTT	TGCTCAAGAAAAGCGTCTGA	NA
SNP1064	Ca_TC12210	*Ca_Desi_Chr07*	[G/A]	1270983	CDS_NON_SYNONYMOUS	bHLH	CATGAAGTTGGCATCTTTGG	CTTCATGCACCCTTGTAGCA	NA
SNP1065	Ca_TC18204	*Ca_Desi_Chr07*	[G/A]	1799554	CDS_NON_SYNONYMOUS	GNAT	GATGCAGATGTAGCCACGAA	CAGCCGCCAGATTTTCTAAC	NA
SNP1066	Ca_TC26550	*Ca_Desi_Chr07*	[G/T]	2712407	CDS_NON_SYNONYMOUS	WRKY	CAACAGAAACCCACAAGCAA	CTTCTGCTGGGTTGAAAAGC	NA
SNP1067	Ca_TC10471	*Ca_Desi_Chr07*	[G/A]	3328637	CDS_NON_SYNONYMOUS	Tify	ATAAACCGTTGATGGCGACT	CTTGCGTTGATGTAGCACCT	NA
SNP1068	Ca_TC04651	*Ca_Desi_Chr07*	[T/C]	3353684	CDS_NON_SYNONYMOUS	ARF	TATTCCGGTTCTGTTCTTATGC	GGCTGATATTACATCGACATACAC	Pr3
SNP1069	Ca_TC03534	*Ca_Desi_Chr07*	[C/T]	3794570	CDS_NON_SYNONYMOUS	SNF2	GAAATGGGACTGGGGAAAAC	GCCTTCTCATGGGGTGAGTA	NA
SNP1070	Ca_TC06361	*Ca_Desi_Chr07*	[T/C]	4598134	CDS_NON_SYNONYMOUS	C2C2-GATA	CGCGTGTGTCTTGTGTTTTAG	CCAAAATTGGACTTACAATCATCA	NA
SNP1071	Ca_TC02595	*Ca_Desi_Chr07*	[T/C]	4624953	CDS_NON_SYNONYMOUS	ARR-B	CGAAGGCTGAGTGGAGTTTC	GCCTTGCCTTGTGCATATTT	NA
SNP1072	Ca_TC03877	*Ca_Desi_Chr07*	[T/C]	5280205	CDS_NON_SYNONYMOUS	mTERF	TTCCCTAATCGGTCGTTTCA	TGAAGACCTGTTTTCTCATTCG	NA
SNP1073	Ca_TC18473	*Ca_Desi_Chr07*	[T/C]	6812937	CDS_NON_SYNONYMOUS	C2C2-YABBY	CACAAACCAAGTAAAGGTTTGGA	GAAGGGAAAGTAGCATTGTTGC	NA
SNP1074	Ca_TC06201	*Ca_Desi_Chr07*	[T/C]	7863835	CDS_NON_SYNONYMOUS	bHLH	GCGCATTGACATTGGTACAG	ACAAGATCCAAGGCCATCTG	NA
SNP1075	Ca_TC10269	*Ca_Desi_Chr07*	[G/A]	8553174	CDS_NON_SYNONYMOUS	zf-HD	GGACAAGCATTCCCTGGTTA	CAGAAATTGAGCAATCTGTGTCT	NA
SNP1076	Ca_TC09491	*Ca_Desi_Chr07*	[G/C]	10016133	CDS_NON_SYNONYMOUS	MYB-related	TCATGTCTCGGATCCTCTGA	TCGAAGAGAATTCATGACCAAA	NA
SNP1077	Ca_TC08990	*Ca_Desi_Chr07*	[G/T]	10114849	CDS_NON_SYNONYMOUS	AP2-EREBP	TCCTCCACGTTCAAAATCAA	TTAACCACCGGTTTCTCCTC	NA
SNP1078	Ca_TC01257	*Ca_Desi_Chr07*	[G/A]	10251662	CDS_NON_SYNONYMOUS	C2C2-GATA	CACACCACTTTGGCGTAATG	TTTAGCGGTATCAACCACAGG	NA
SNP1079	Ca_TC11857	*Ca_Desi_Chr07*	[G/A]	10485720	CDS_NON_SYNONYMOUS	SRS	CCAGCTTCACGCTTCTAGG	TTTTACATAGCATTTTCCTTGAACA	NA

SNP IDs	*Kabuli* /*desi* gene accession IDs	*Kabuli* /*desi* chromosomes	SNPs	Physical positions (bp)	Sequence components of genome	Putative functions	Forward primer sequences (5'-3')	Reverse primer sequences (5'-3')	Primers used in Figure 2A
SNP1080	Ca_TC08009	*Ca_Desi_Chr07*	[T/C]	10686471	CDS_NON_SYNONYMOUS	ARR-B	AAAAAGAAAGCGGGGAACAT	CTTTGTTTTGGCATGTGGTG	NA
SNP1081	Ca_TC15323	*Ca_Desi_Chr07*	[G/A]	11192772	CDS_NON_SYNONYMOUS	HB	TCAAACAAAACACCATCACCA	AAAGGTTGTGTGGGTGGAAC	NA
SNP1082	Ca_TC16765	*Ca_Desi_Chr07*	[A/T]	11324293	CDS_NON_SYNONYMOUS	bZIP	AGGTGGTTTATTCTGCGATGA	TTTTCCCTTTCTCCCTCCTC	NA
SNP1083	Ca_TC06556	*Ca_Desi_Chr07*	[A/G]	11546194	CDS_NON_SYNONYMOUS	LOB	TTGTTCCCAAACCACACCTT	AAACAAATCACCGACGAACC	NA
SNP1084	Ca_TC00252	*Ca_Desi_Chr07*	[A/G]	14470793	CDS_NON_SYNONYMOUS	FHA	GTTGGAAACCACAAAAACATAAT	TGAAGTCGAATCGTCTGCTG	NA
SNP1085	Ca_TC08380	*Ca_Desi_Chr07*	[G/A]	17264124	CDS_NON_SYNONYMOUS	MYB-related	GATAGAGTAGTGATGAGAAGATGAAGG	GTATCATGGGTCTCTGACATATTG	NA
SNP1086	Ca_TC08333	*Ca_Desi_Chr07*	[T/C]	17317735	CDS_NON_SYNONYMOUS	SET	GGAAAGAAGATTGGCTGCTG	CTCATCGGAGGGATGTTCTC	NA
SNP1087	Ca_TC01462	*Ca_Desi_Chr07*	[G/A]	17780711	CDS_NON_SYNONYMOUS	bZIP	GTACCCCATTTTGAGGAGCA	ACTGGTTCCTGACCAAATGC	NA
SNP1088	Ca_TC00629	*Ca_Desi_Chr07*	[G/A]	18389220	CDS_NON_SYNONYMOUS	Jumonji	ATGCTGGAGAAGGCTGAAGA	TCACCGATCGATCCCTCTAC	NA
SNP1089	Ca_TC08611	*Ca_Desi_Chr07*	[C/T]	20367403	CDS_NON_SYNONYMOUS	bHLH	TATGGCATTGGATTGGAGGT	GCACACTGAACAAGCTTGAAA	NA
SNP1090	Ca_TC05041	*Ca_Desi_Chr07*	[G/A]	22826866	CDS_NON_SYNONYMOUS	bHLH	TGTTTCACAAACAAACACAAATCTC	TGGTCAGTTGAAGATTATACTACTA	NA
SNP1091	Ca_TC18227	*Ca_Desi_Chr07*	[C/A]	27161185	CDS_NON_SYNONYMOUS	Alfin-like	CGAAGCGTCACAGTCACAAA	GCACGTGAGCGTGTAATGTT	NA
SNP1092	Ca_TC17994	*Ca_Desi_Chr07*	[C/T]	33299885	CDS_NON_SYNONYMOUS	Tify	TGATCCGAAGTGTTGGACCT	TCATTTCACACCCTCTAACCAA	NA
SNP1093	Ca_TC16069	*Ca_Desi_Chr07*	[G/C]	39769428	CDS_NON_SYNONYMOUS	C3H	TCAAGGTTCGTCCTTGTTCC	GTAGCCAAGCCTCCTCCTTT	NA
SNP1094	Ca_TC03228	*Ca_Desi_Chr07*	[T/A]	42243517	CDS_NON_SYNONYMOUS	RB	CATGCGTCTGAATACCATCG	CACTAGCACATTGCGTTGCT	NA
SNP1095	Ca_TC03228	*Ca_Desi_Chr07*	[T/C]	42245921	CDS_NON_SYNONYMOUS	RB	TTTTCTCAATTTCGCAGCAA	CATCGCTTGTGACATCCTTG	NA
SNP1096	Ca_TC03228	*Ca_Desi_Chr07*	[T/C]	42246393	CDS_NON_SYNONYMOUS	RB	CATGCGTCTGAATACCATCG	CACTAGCACATTGCGTTGCT	NA
SNP1097	Ca_TC15016	*Ca_Desi_Chr07*	[G/T]	43044483	CDS_NON_SYNONYMOUS	CCAAT	TCCGTAACCGACGTGTAACTC	CCATGCAGGATCATTACACC	NA
SNP1098	Ca_TC11984	*Ca_Desi_Chr07*	[G/A]	47237682	CDS_NON_SYNONYMOUS	HB	TCCAAATAGGCACCAACACA	AACCATGGCCATAATCCAGA	NA
SNP1099	Ca_TC02570	*Ca_Desi_Chr08*	[T/C]	156866	CDS_NON_SYNONYMOUS	SNF2	GTCATTCAGGCAGTTGACCA	CTCTCTGCTCGCAAGAGGTT	NA
SNP1100	Ca_TC33713	*Ca_Desi_Chr08*	[G/A]	498219	CDS_NON_SYNONYMOUS	FHA	TTCTGAAGCGAGAAGAGGAGA	GCCCAATACCATGTTGCCTA	NA
SNP1101	Ca_TC00233	*Ca_Desi_Chr08*	[G/A]	510120	CDS_NON_SYNONYMOUS	SNF2	TGCAGGAATTGTGGTCTCTG	AAACCACTTACGCCGACATC	NA
SNP1102	Ca_TC02881	*Ca_Desi_Chr08*	[G/A]	666619	CDS_NON_SYNONYMOUS	GNAT	TGGGATCGTTGTCAAAAACA	AAGGTATAGCAAACGGGCATT	NA
SNP1103	Ca_TC12027	*Ca_Desi_Chr08*	[G/A]	891777	CDS_NON_SYNONYMOUS	GNAT	GAGCGAAGGCATTCATTGAT	GTACACTCGACCGCCGTATT	NA
SNP1104	Ca_TC02610	*Ca_Desi_Chr08*	[T/C]	1261904	CDS_NON_SYNONYMOUS	C3H	AATGGTGAGGATTTGGTGGA	TAAAGGGAAGCCATGTCACC	NA
SNP1105	Ca_TC03581	*Ca_Desi_Chr08*	[C/T]	1434072	CDS_NON_SYNONYMOUS	AP2-EREBP	AAGCAAAGCTGAATTGACAGC	CCTCTGTTTACATTCGCCAAA	NA
SNP1106	Ca_TC00629	*Ca_Desi_Chr08*	[T/C]	1753840	CDS_NON_SYNONYMOUS	Jumonji	TTCAATTTCAATCTCTGAAACAGTG	TCACATTCTCCATCTTCAAACG	NA

SNP IDs	*Kabuli* /*desi* gene accession IDs	*Kabuli* /*desi* chromosomes	SNPs	Physical positions (bp)	Sequence components of genome	Putative functions	Forward primer sequences (5'-3')	Reverse primer sequences (5'-3')	Primers used in Figure 2A
SNP1107	Ca_TC18250	*Ca_Desi_Chr08*	[A/T]	2011749	CDS_NON_SYNONYMOUS	HB	TGGTCCTCAACCTCAACCTC	CCAAATATTCCATTTCCAACCA	NA
SNP1108	Ca_TC06727	*Ca_Desi_Chr08*	[A/C]	2580625	CDS_NON_SYNONYMOUS	AP2-EREBP	CACAAATCCGAACAAAACCA	TACGAATCCGATTTGGCTCT	NA
SNP1109	Ca_TC15360	*Ca_Desi_Chr08*	[G/C]	2664948	CDS_NON_SYNONYMOUS	C2C2-GATA	GTGAGTATGCTGCCGAGCTA	GGTGGAGGACAAAAGGAACA	NA
SNP1110	Ca_TC18784	*Ca_Desi_Chr08*	[A/C]	2704215	CDS_NON_SYNONYMOUS	Tify	CAACAACATGAATCATCATCACA	TTGATGGAGGAATTAGTATGAC	NA
SNP1111	Ca_TC15831	*Ca_Desi_Chr08*	[C/T]	2974401	CDS_NON_SYNONYMOUS	ARR-B	AATGCAAAGGACTTGCCAAC	TCCAGTCTACATGGCATTGG	NA
SNP1112	Ca_TC12117	*Ca_Desi_Chr08*	[G/T]	3154021	CDS_NON_SYNONYMOUS	bHLH	CATCGTCCAACGAATTAGCA	CAACCCCCATACCAAACAAG	NA
SNP1113	Ca_TC16659	*Ca_Desi_Chr08*	[T/C]	3396641	CDS_NON_SYNONYMOUS	BSD	ATCCACTCAACCCGATTCAA	CCCAGTGGAGACTGGCTTTA	NA
SNP1114	Ca_TC16559	*Ca_Desi_Chr08*	[A/T]	3456812	CDS_NON_SYNONYMOUS	GNAT	CACCTTGGAATTCGGTTTGT	AATCACCCTTCCCTCCTTGT	NA
SNP1115	Ca_TC06892	*Ca_Desi_Chr08*	[G/A]	3521637	CDS_NON_SYNONYMOUS	GRAS	CCATCACCGTTAAAATCACG	CGGCATCACTACACTGCATC	NA
SNP1116	Ca_TC15081	*Ca_Desi_Chr08*	[A/T]	3682384	CDS_NON_SYNONYMOUS	LOB	ATCCTGTGCCGGTGTCTTAC	CCTCCTCTTCCCAATTTTCC	NA
SNP1117	Ca_TC21141	*Ca_Desi_Chr08*	[G/T]	3998501	CDS_NON_SYNONYMOUS	AP2-EREBP	ACAATGCCAAAATTTCTTCTGT	CGTGTAAGCTCACCCAGATG	NA
SNP1118	Ca_TC03617	*Ca_Desi_Chr08*	[C/T]	4056195	CDS_NON_SYNONYMOUS	SET	AGACTGCGCCCTCTATGTGT	GCAGCCAAAGTGAAAGTGCT	NA
SNP1119	Ca_TC16950	*Ca_Desi_Chr08*	[G/C]	4176714	CDS_NON_SYNONYMOUS	FHA	TTGGAGAGGATCTTGTCACCTT	TGGGAGAGTTGGAGGAATGT	NA
SNP1120	Ca_TC07096	*Ca_Desi_Chr08*	[G/A]	4421071	CDS_NON_SYNONYMOUS	LOB	AGTGCCTTTTCATCTTCTCCTAC	AACATGGGTATTACGGTTACAGG	NA
SNP1121	Ca_TC07629	*Ca_Desi_Chr08*	[G/A]	4623320	CDS_NON_SYNONYMOUS	HB	CACCAGAAGTGGACCCAAAT	TCTCCTCCAACTCCACATCC	NA
SNP1122	Ca_TC04279	*Ca_Desi_Chr08*	[A/T]	5413114	CDS_NON_SYNONYMOUS	SRS	TGTTTTTGTTTGTGTGATGCAA	TTTGATTGGGGTTTGATGAAT	NA
SNP1123	Ca_TC08560	*Ca_Desi_Chr08*	[G/T]	5508790	CDS_NON_SYNONYMOUS	C2C2-GATA	TCCCTCAAACAACACAACCA	AGCCTAACCTAGCGCACTCA	NA
SNP1124	Ca_TC08666	*Ca_Desi_Chr08*	[T/C]	5564404	CDS_NON_SYNONYMOUS	C3H	TGCCCTTTTTCACTTGCTCT	GCAGAAGAAGGACGATGAGG	NA
SNP1125	Ca_TC19323	*Ca_Desi_Chr08*	[G/T]	6618347	CDS_NON_SYNONYMOUS	HMG	CGGCTGAACCCAAAATTAAG	CCAAAAACCCTCACTCTTCG	NA
SNP1126	Ca_TC05032	*Ca_Desi_Chr08*	[G/A]	7520805	CDS_NON_SYNONYMOUS	Trihelix	CTGGAGAGGGATTTCACTGC	TCCCTGGACTTAATGCCAAA	NA
SNP1127	Ca_TC00538	*Ca_Desi_Chr08*	[A/T]	7772710	CDS_NON_SYNONYMOUS	E2F-DP	GCAATTTAGGAGCCAAAACG	TTTCCCAGTCCAGCCTACAC	NA
SNP1128	Ca_TC02177	*Ca_Desi_Chr08*	[G/A]	8362560	CDS_NON_SYNONYMOUS	HB	TGCAAACAATGCCTTTTCAA	CGGGAGAAGGTATCACTCCA	NA
SNP1129	Ca_TC05885	*Ca_Desi_Chr08*	[T/C]	11675561	CDS_NON_SYNONYMOUS	Tify	CCCTTTCTTTTCGCTTCTCC	GGAGAGGGAATTCCTTGGAT	NA
SNP1130	Ca_TC08051	*Ca_Desi_Chr08*	[G/A]	12525828	CDS_NON_SYNONYMOUS	MYB-related	AGCGCCGAAAGTTCTATTCA	GCGCTGGTATGTGTCCCTAT	NA
SNP1131	Ca_TC14771	*Ca_Desi_Chr08*	[T/C]	13906178	CDS_NON_SYNONYMOUS	C2C2-CO-like	CTGGGAAATAATTGAAGGGTTA	AAGGTGATGCAGATTCAGGTG	NA
SNP1132	Ca_TC03533	*Ca_Desi_Chr08*	[A/G]	14719923	CDS_NON_SYNONYMOUS	SWI/SNF-SWI3	GAACCAAAATCCCAAAACGA	TCAAGTCCTCAGCCAAGTGA	NA
SNP1133	Ca_TC12255	*Ca_Desi_Chr08*	[T/A]	16090020	CDS_NON_SYNONYMOUS	C3H	AATCTCCGAGAAGGTGATTGA	CGGTGACATCCAAAACTCCT	NA

Eight seed weight-associated genes with SNPs are highlighted
