# Supplementary material for: EcoTILLING-Based Association Mapping Efficiently Delineates Functionally Relevant Natural Allelic Variants of Candidate Genes Governing Agronomic Traits in Chickpea
Source: Front Plant Sci. 2016 Apr 19;7:450. doi: 10.3389/fpls.2016.00450 (PMC4835497; doi:10.3389/fpls.2016.00450)
Supplement: Supplementary file 3 [file Image1.PDF]

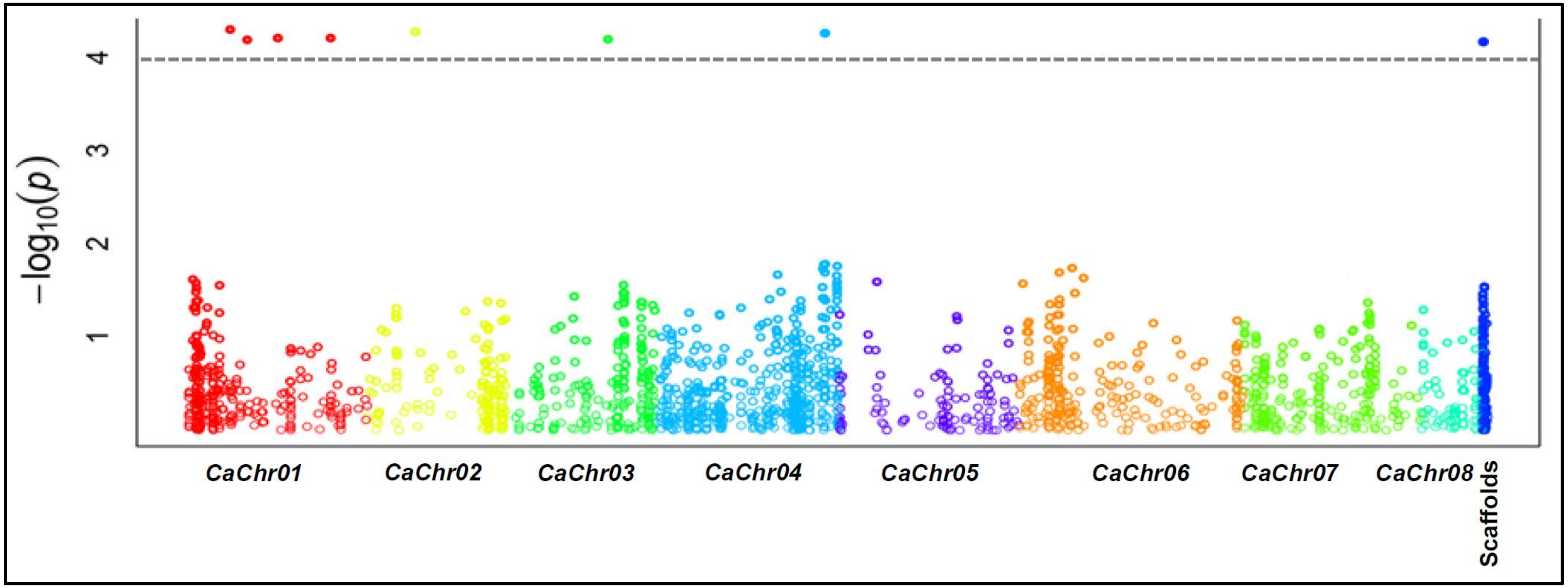

Figure S1. GWAS-based Manhattan plot depicting significant P-values associated with 100-seed weight using 1133 transcription factor gene-derived SNPs in chickpea. The relative density of SNPs physically mapped on eight chromosomes and scaffolds of *kabuli* genome are represented by the x-axis. The y-axis indicates the $-\log_{10}$ (P)-value for significant association of eight SNP loci with seed weight. The SNPs exhibiting significant association with seed weight at a cut-off P value $\leq 1 \times 10^{-4}$ are demarcated with a dotted line.
